# Isoxazole–Thiazole Hybrids: Synthesis, Structural Characterisation, Carbonic Anhydrase Inhibition, and Molecular Docking Studies

**DOI:** 10.3390/molecules31111824

**Published:** 2026-05-25

**Authors:** Nurcan Berber, Özge Nur Türkeri, Faika Başoğlu, Kubra Çıkrıkcı, Adem Ergün, Nahit Gencer

**Affiliations:** 1 Department of Pharmacy Services, Vocational School of Health Services, Çanakkale 18 Mart University, Çanakkale 17000, Türkiye; nberber@comu.edu.tr (N.B.); ozgenur.turkeri@comu.edu.tr (Ö.N.T.); 2 Pharmaceutical Chemistry, Faculty of Pharmacy, Near East University, North Cyprus TR-10, Mersin 99138, Türkiye; fabasoglu@eul.edu.tr; 3 DESAM Institute, Near East University, North Cyprus Via TR-10, Mersin 99138, Türkiye; 4 Department of Chemistry, Faculty of Arts and Sciences, Balikesir University, Balikesir 10000, Türkiye; kcikrikci10@gmail.com (K.Ç.); ademergun@balikesir.edu.tr (A.E.)

**Keywords:** isoquinoline, thiazole, thiourea, carbonic anhydrase, enzyme inhibitor

## Abstract

A new series of isoxazole-fused thiazole–oxazole derivatives (**11a–n**) was rationally designed and synthesised with the aim of developing potent carbonic anhydrase (CA) I and II inhibitors. The synthesis was achieved in five steps starting from 4-bromoacetophenone, involving key intermediates such as hydroxylamine hydrochloride, hydrazine hydrate, thioisocyanate, and various phenacyl bromide derivatives, using ethanol, triethylamine, tetrahydrofuran (THF), and dimethylformamide (DMF) as solvents. The synthetic route included the formation of a β-ketoester, isoxazole ester, hydrazine adduct, thiourea derivative, and, ultimately, a thiazole ring. The structures of the final compounds were confirmed by ^1^H-NMR, ^13^C-NMR, IR spectroscopy, and elemental analysis. All compounds were examined as inhibitors of human carbonic anhydrase (hCA) I and II, and all of them inhibited hCA I and hCA II. Kinetic investigation results revealed that these compounds inhibited hCA I and hCA II in a non-competitive manner. To further explore the molecular basis of their inhibitory activity, in silico studies, including molecular docking and 300 ns molecular dynamics (MD) simulations, were carried out against both CA I and CA II isoforms. These simulations provided detailed insights into the dynamic behaviour, stability, and key binding interactions of the compounds within the enzyme active sites, supporting their potential as promising carbonic anhydrase inhibitors.

## 1. Introduction

**Figure 1:**
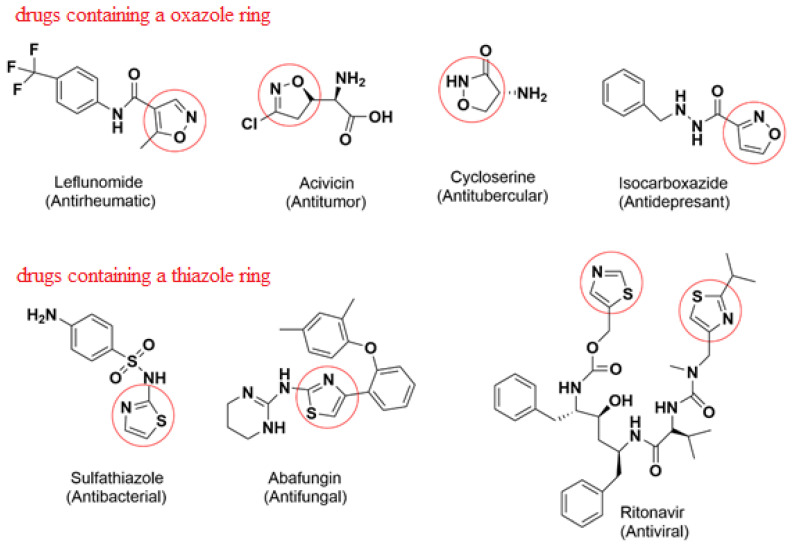
Commercially marketed drugs containing thiazole and isoxazole structures.

Five-membered heterocyclic rings containing nitrogen (N), oxygen (O), or sulfur (S) are ubiquitous in vitamins, hormones, alkaloids, and a variety of other natural products, as well as in pharmaceutical molecules and synthetic organic compounds [1,2,3]. The classes of five-membered aromatic heterocycles that have undergone the most thorough investigation are isoxazoles and thiazoles [4]. They represent a highly effective and useful group of heterocyclic compounds. Isoxazoles are widely used in the pharmaceutical industry. They are well known to exhibit a wide range of biological activities, such as anticancer, antitumor, antibacterial, antitubercular, antidepressant, antimicrobial, antiviral, antirheumatic, and antidiabetic activities [5,6,7,8,9,10,11,12], and are frequently encountered as intermediates in the synthesis of natural heterocyclic structures [13]. They are also used in agriculture as insecticides to manage *Callosobruchus chinensis*, as well as photochromic compounds in dye-sensitised solar cells, high-energy materials, and liquid crystals in materials science [14]. Compounds containing thiazole moiety have gained significant attention in drug discovery due to their diverse biologically active properties, including anticancer [15], antifungal [16], antibacterial [17], anti-inflammatory [18], antidiabetic [19], anticonvulsant [20], and antioxidant properties [21]. The structures of commercially marketed drugs that are currently in use and contain thiazole and oxazole structures are shown in Figure 1.

In drug design and development, a proper structure–activity relationship analysis must be carried out to identify multitarget compounds with balanced pharmacological activities. The molecular hybridisation method is a very suitable method for this purpose. This method enables the rational design of multi-target ligands by combining the pharmacophore properties of different templates. It is also an important approach in terms of increasing therapeutic efficacy and providing the right target combination [22,23,24,25].

Carbonic anhydrases (CAs, EC 4.2.1.1) are a family of metalloenzymes, most of which contain a zinc ion in their active site. They play a vital role in maintaining physiological pH by catalysing the reversible hydration of carbon dioxide into bicarbonate and a proton. It is well known that disturbances in CA activity are associated with several diseases, including glaucoma, epilepsy, obesity, and, as more recently identified, cancer. Therefore, inhibiting carbonic anhydrases is considered an important therapeutic strategy in the treatment of these diseases. Sulfonamides represent the primary class of carbonic anhydrase inhibitors (CAIs) and have been employed clinically for over five decades, particularly in the treatment of glaucoma. Despite their efficacy, many conventional CAIs often exhibit off-target effects due to their lack of selectivity across multiple human CA isoforms. In addition to sulfonamides, other notable CAIs include phenolic compounds, coumarins, and, more recently, carboxylic acid derivatives. Supuran and collaborators demonstrated that various chemical scaffolds, such as sulfonamides, coumarin–sulfonamide hybrids, imidazoles, and saccharin-based molecules, can effectively inhibit the transmembrane cancer-related isoform [26,27,28,29].

However, despite the significant biological activities reported for isoxazole [5,6,7,8,9,10,11,12] and thiazole derivatives [15,16,17,18,19,20,21], their combined use within a single hybrid scaffold has been scarcely explored, particularly for selective carbonic anhydrase inhibition. This structural gap prompted us to design novel hybrid molecules with the aim of achieving enhanced inhibitory potency (low IC_50_ values) against hCA isoforms.

This work focuses on the design and synthesis of novel isoxazole-fused thiazole–oxazole hybrids. We utilised diethyl oxalate, hydroxylamine hydrochloride, thioisocyanate, and phenacyl bromide derivatives in the preparation of these scaffolds. The newly synthesised compounds were investigated in vitro for their inhibitory activity against hCA isoforms I and II using both hydratase and esterase assays. Moreover, the intermolecular interactions and docking scores were thoroughly investigated to understand the binding mechanisms of these compounds.

## 2. Materials and Methods

### 2.1. Chemical Studies

All starting materials and reagents were purchased from commercial suppliers. Reactions were monitored by TLC; the plates were visualised with short-wave UV fluorescence (k = 254 nm). Melting points were taken on a JHX-4 melting point apparatus and were uncorrected. IR spectra (ν, cm^−1^) were measured on a SHIMADZU Prestige-21 (200 VCE) spectrometer (Shimadzu, Kyoto, Japan). ^1^H NMR and ^13^C NMR spectra were recorded on a Bruker AV-400 MHz instrument (Bruker, Billerica, MA, USA) and JEOL instruments (JEOL LNM-GSX 400a and JEOL JMN-ECP 400; JEOL, Tokyo, Japan) in DMSO-d_6_ using TMS as the internal standard. Elemental analysis was carried out using a Leco instrument (Leco Corporation, St. Joseph, MI, USA). All chemicals were purchased from Merck (Darmstadt, Germany) and Sigma-Aldrich (Taufkirchen, Germany).

#### 2.1.1. Synthesis of β-Ketoester (**3**)

An ethanolic solution of sodium ethoxide was obtained by slowly adding freshly cut metallic sodium (1.20mmol) to absolute ethanol at low temperatures. After approximately 45 min, 4-bromo acetophenone (1mmol) and diethyl oxalate (1.10 mmol) dissolved in 1 mL DMF were added to the system and stirred at room temperature overnight. At the end of the period, the mixture was poured into cold water and acidified with 1N hydrochloric acid to afford a solid, which was filtered, washed with water, and dried to give the corresponding β-ketoester derivative (**3**). Product (**3**) was filtered, washed with water, and dried, resulting in a 78% yield of brown solids [25].

#### 2.1.2. Synthesis of Isoxazole Ester (**5**)

To the solution of β-ketoester (**3**) (1 mmol) in ethanol, NH_2_OH.HCl (1.25 mmol) was added, and the resulting mixture was first stirred for 2 h at room temperature and then refluxed for 5h at 80 °C. After the completion of the reaction, the reaction mixture was cooled and poured into cold water acidified with 1N hydrochloric acid to afford a solid, which was filtered, washed with water, and dried to give the corresponding isoxazole ester (**5**); this was collected by filtration and dried, resulting in a 100% yield of white solid precipitate.

#### 2.1.3. Synthesis of 5-(4-bromophenyl)isoxazole-3-carbohydrazide (**7**)

The compound (**5**) (1 mmol) was dissolved in ethanol, and then hydrazine hydrate (1.5 mmol) was added. The reaction mixture was refluxed for 24 h at 80 °C. After the completion of the reaction, the mixture was cooled and formed the solid product (**7**); this was collected by filtration and dried, resulting in a 94% yield of white solid precipitate [30].

#### 2.1.4. General Synthesis of Thiourea Derivatives (**9a–e**)

A mixture of the compound (**7**) (1.0 mmol) and thioisocyanate derivatives (1.0 mmol), with the addition of three drops of triethylamine, was stirred and refluxed at 70 °C for 24 h in tetrahydrofuran (THF). After completion of the reaction, the mixture was cooled to room temperature and then poured into cold water. Product (**9a**–**e**) was filtered, washed with water, and dried, resulting in a 93–98% yield of beige solid precipitate [31].

#### 2.1.5. General Synthesis of Thiazole Derivatives (**11a–n**)

**Scheme 1:**
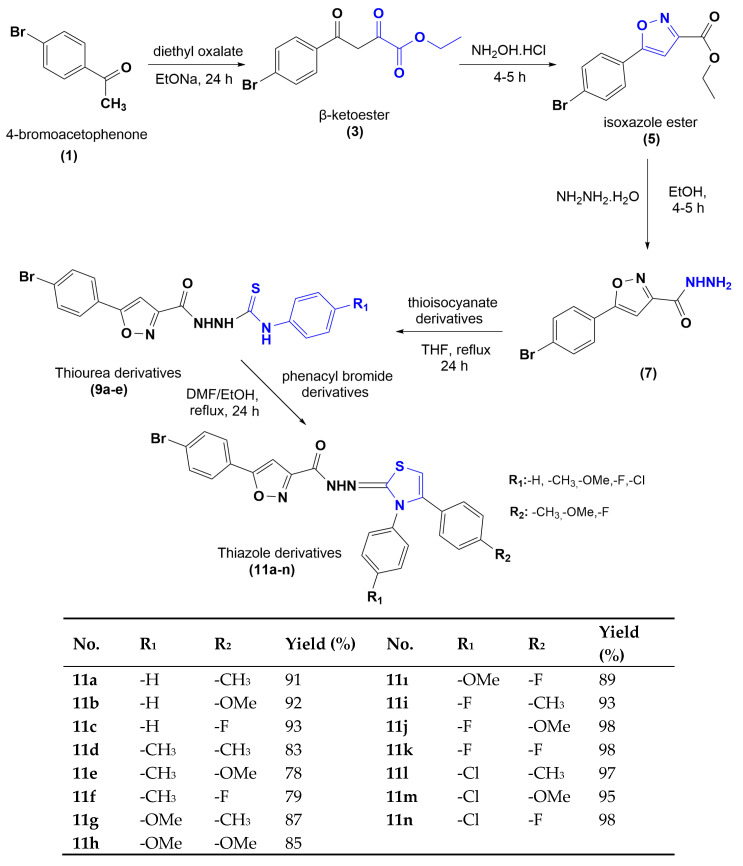
General synthesis procedure for target compounds. The blue color was used to clearly indicate the added groups and the formed structures after the reaction.

Thiourea derivatives (**9a**–**e**) (1 mmol) and phenacyl bromide derivatives (1 mmol) in EtOH/DMF (5:5 *v*/*v*) were stirred and refluxed at 60 °C for 24 h. After completion of the reaction, the mixture was allowed to cool to room temperature and poured into cold water. The product (**11a–n**) was filtered, washed with water, and dried, resulting in a 78–98% yield of beige solid precipitate [32]. All compounds were synthesised as shown in Scheme 1.

##### 5-(4-bromophenyl)-N-(3-phenyl-4-p-tolylthiazol-2 (3H)-ylidene)isoxazole-3-carbohydrazide (**11a**)

Beige solid; yield: 91%; m.p.: 124.5–126.5 °C; IR (KBr, cm^−1^): 3121, 2919, 2838, 1693, 1585, 1488, 1440, 1227; ^1^H NMR (300 MHz, DMSO) δ: 11.08 (1H, -NH), 7.89–7.05 (13H, Ar-H), 6.50 (1H, oxazole ring proton), 6.38 (1H, thiazole ring proton), 2.27–2.21 (3H, -CH_3_ proton);^13^C NMR (75 MHz, DMSO) δ: 169.8, 160.4, 160.0, 158.4, 158.1, 147.3, 138.4, 133.0, 131.6, 130.4, 129.7, 128.5, 125.7, 125.1, 123.1, 123.0, 120.6, 118.2, 117.0, 116.7, 114.6, 114.4, 112.9, 101.3, 101.1, 93.9, 27.0; Anal. Calcd for C_26_H_19_BrN_4_O_2_S: C, 58.76; H, 3.60; Br, 15.04; N, 10.54; S, 6.03; Found: C, 58.79; H, 3.57; Br, 15.00; N, 10.57; S, 6.00%.

##### 5-(4-bromophenyl)-N’-(4-(4-methoxyphenyl)-3-phenylthiazol-2(3H)-ylidene)isoxazole-3-carbohydrazide (**11b**)

Beige solid; yield: 95%; m.p.: 116.8–118.8 °C; IR (KBr, cm^−1^): 3115, 2933, 2838, 1669, 1607, 1507, 1444, 1250; ^1^H NMR (300 MHz, DMSO) δ: 11.06 (1H, -NH), 7.88–6.94 (13H, Ar-H), 6.44 (1H, oxazole ring proton), 6.32 (1H, thiazole ring proton), 3.72–3.68 (3H, -OMe); ^13^C NMR (75 MHz, DMSO) δ:169.8, 163.3, 160.4, 160.0, 158.4, 158.1, 147.3, 138.5, 137.6, 137.2, 133.0, 131.6, 130.4, 129.7, 128.5, 125.7, 123.1, 123.0, 122.6, 122.5, 122.4, 117.0, 116.7, 114.6, 114.4, 101.3, 55.8, 55.7; Anal. Calcd for C_26_H_19_BrN_4_O_3_S: C, 57.05; H, 3.50; Br, 14.60; N, 10.23; S, 5.86; Found: C, 57.08; H, 3.53; Br, 14.62; N, 10.25; S, 5.88%.

##### 5-(4-bromophenyl)-N’-(4-(4-fluorophenyl)-3-phenylthiazol-2(3H)-ylidene)isoxazole-3-carbohydrazide (**11c**)

Beige solid; yield: 93%; m.p.: 113.5–115.5 °C; IR (KBr, cm^−1^): 3115, 2977, 2895, 1659, 1585, 1505, 1444, 1226; ^1^H NMR (300 MHz, DMSO) δ: 11.09 (1H, -NH), 7.88–6.95 (13H, Ar-H), 6.56 (1H, oxazole ring proton), 6.45 (1H, thiazole ring proton); ^13^C NMR (75 MHz, DMSO) δ: 170.7, 169.8, 169.5, 159.7, 158.5, 158.0, 157.5, 154.9, 139.0, 138.5, 133.0, 131.5, 131.4, 130.6, 130.5, 128.5, 128.4, 126.1, 125.7, 125.3, 125.0, 123.1, 116.7, 116.2, 95.5; Anal. Calcd for C_25_H_16_BrFN_4_O_2_S: C, 56.09; H, 3.01; Br, 14.92; F, 3.55; N, 10.46; S, 5.99; Found: C, 57.00; H, 3.03; Br, 14.94; F, 3.51; N, 10.48; S, 5.95%.

##### 5-(4-bromophenyl)-N’-(3,4-dip-tolylthiazol-2(3H)-ylidene)isoxazole-3-carbohydrazide (**11d**)

Beige solid; yield: 83%; m.p.: 122.5–124.5 °C; IR (KBr, cm^−1^): 3121, 2919, 2838, 1694, 1604, 1509, 1442, 1228; ^1^H NMR (300 MHz, DMSO) δ: 11.05 (1H, -NH), 7.89–7.04 (12H, Ar-H), 6.87 (1H, oxazole ring proton), 6.49 (1H, thiazole ring proton), 2.27 (3H, -CH_3_), 2.26 (3H, -CH_3_); ^13^C NMR (75 MHz, DMSO) δ: 169.8, 158.4, 140.4, 138.7, 135.5, 133.0, 130.7, 130.1, 129.9, 129.6, 129.1, 128.8, 128.5, 128.2, 121.6, 104.6, 21.5, 21.4; Anal. Calcd for C_27_H_21_BrN_4_O_2_S: C, 59.45; H, 3.88; Br, 14.65; N, 10.27; S, 5.88; Found: C, 59.48; H, 3.90; Br, 14.67; N, 10.30; S, 6.00%.

##### 5-(4-bromophenyl)-N’-(4-(4-methoxyphenyl)-3-p-tolylthiazol-2(3H)-ylidene)isoxazole-3-carbohydrazide (**11e**)

Beige solid; yield: 78%; m.p.: 113.4–115.4 °C; IR (KBr, cm^−1^): 3122, 2921, 2919, 1694, 1603, 1508, 1443, 1250; ^1^H NMR (300 MHz, DMSO) δ: 11.02 (1H, -NH), 7.89–6.77 (12H, Ar-H), 6.43 (1H, oxazole ring proton), 6.32 (1H, thiazole ring proton), 3.73–3.68 (3H, -OMe), 2.25 (3H, -CH_3_); ^13^C NMR (75 MHz, DMSO) δ: 170.6, 169.8, 160.5, 159.9, 158.5, 158.2, 154.9, 133.0, 130.70, 130.4, 130.1, 129.7, 129.1, 128.5, 128.4, 121.5, 114.6, 114.4, 101.4, 101.2, 98.5, 55.9, 21.3, 21.1; Anal. Calcd for C_27_H_21_BrN_4_O_3_S: C, 57.76; H, 3.77; Br, 14.23; N, 9.98; S, 5.71; Found: C, 57.78; H, 3.79; Br, 14.25; N, 10.01; S, 5.73%.

##### 5-(4-bromophenyl)-N’-(4-(4-fluorophenyl)-3-p-tolylthiazol-2(3H)-ylidene)isoxazole-3-carbohydrazide (**11f**)

Beige solid; yield: 79%; m.p.: 110.3–112.30 °C; IR (KBr, cm^−1^): 3117, 2976, 2921, 1688, 1601, 1505, 1444, 1229; ^1^H NMR (300 MHz, DMSO) δ: 11.05 (1H, -NH), 7.89–6.82 (12H, Ar-H), 6.55 (1H, oxazole ring proton), 6.44 (1H, thiazole ring proton), 2.25–2.12 (3H, -CH_3_); ^13^C NMR (75 MHz, DMSO) δ: 169.8, 167.6, 167.1, 162.2, 159.9, 156.7, 154.9, 148.0, 143.70, 139.2, 138.0, 133.0, 131.3, 130.7, 130.1, 129.1, 128.5, 128.4, 125.8, 121.4, 116.2, 115.9, 101.3, 99.9, 21.4; Anal. Calcd for C_26_H_18_BrFN_4_O_2_S: C, 56.84; H, 3.30; Br, 14.54; F, 3.46; N, 10.20; S, 5.84; Found: C, 56.87; H, 3.32; Br, 14.55; F, 3.48; N, 10.23; S, 5.83%.

##### 5-(4-bromophenyl)-N’-(3-(4-methoxyphenyl)-4-p-tolylthiazol-2(3H)-ylidene)isoxazole-3-carbohydrazide (**11g**)

Beige solid; yield: 87%; m.p.: 127.2–129.2 °C; IR (KBr, cm^−1^): 3122, 2920, 2837, 1697, 1607, 1502, 1444, 1229; ^1^H NMR (300 MHz, DMSO) δ: 10.98 (1H, -NH), 7.88–6.88 (12H, Ar-H), 6.45 (1H, oxazole ring proton), 6.37 (1H, thiazole ring proton), 3.72 (3H, -OMe), 2.27–2.21 (3H, -CH_3_); ^13^C NMR (75 MHz, DMSO) δ: 170.7, 169.4, 164.2, 158.4, 158.1, 157.8, 157.3, 149.7, 148.4, 146.6, 139.7, 139.5, 133.0, 131.2, 130.1, 129.8, 128.8, 128.5, 128.1, 127.6, 127.3, 125.3, 123.4, 101.4, 94.9, 49.8, 21.5; Anal. Calcd for C_27_H_21_BrN_4_O_3_S: C, 57.76; H, 3.77; Br, 14.23; N, 9.98; S, 5.71; Found: C, 57.78; H, 3.79; Br, 14.21; N, 10.01; S, 5.73%.

##### N’-(3,4-bis(4-methoxyphenyl)thiazol-2(3H)-ylidene)-5-(4-bromophenyl)isoxazole-3-carbohydrazide (**11h**)

Beige solid; yield: 85%; m.p.: 112.5–114.5 °C; IR (KBr, cm^−1^): 3117, 2935, 2837, 1687, 1608, 1507, 1443, 1249; ^1^H NMR (300 MHz, DMSO) δ: 11.04 (1H, -NH), 7.94–6.83 (12H, Ar-H), 6.46 (1H, oxazole ring proton), 6.36 (1H, thiazole ring proton), 3.69–3.35 (3H, -OMe), 2.87-2.71 (3H, -CH_3_); ^13^C NMR (75 MHz, DMSO) δ: 170.7, 169.4, 165.8, 164.1, 160.6, 158.4, 158.1, 157.7, 149.7, 133.0, 130.5, 130.1, 129.8, 129.6, 128.5, 128.4, 127.6, 123.4, 122.5, 114.7, 114.5, 101.4, 94.1, 55.9, 55.5; Anal. Calcd for C_27_H_21_BrN_4_O_4_S: C, 56.16; H, 3.67; Br, 13.84; N, 9.70; S, 5.55; Found: C, 56.19; H, 3.69; Br, 13.87; N, 9.72; S, 5.52%.

##### 5-(4-bromophenyl)-N’-(4-(4-fluorophenyl)-3-(4-methoxyphenyl)thiazol-2(3H)-ylidene)isoxazole-3-carbohydrazide (**11ı**):

Beige solid; yield: 89%; m.p.: 123.2–125.2 °C; IR (KBr, cm^−1^): 3222, 2935, 2837, 1677, 1605, 1508, 1443, 1248; ^1^H NMR (300 MHz, DMSO) δ: 11.07 (1H, -NH), 7.89–6.94 (12H, Ar-H), 6.58 (1H, oxazole ring proton), 6.49 (1H, thiazole ring proton), 3.71–3.63 (3H, -OMe); ^13^C NMR (75 MHz, DMSO) δ: 170.6, 169.8, 160.5, 160.0, 158.5, 158.1, 156.2, 147.4, 145.7, 140.5, 139.5, 138.5, 137.2, 133.0, 131.6, 130.5, 129.7, 128.5, 123.1, 123.0, 117.0, 116.7, 114.6, 114.4, 101.3, 55.7; Anal. Calcd for C_26_H_18_BrFN_4_O_3_S: C, 55.23; H, 3.21; Br, 14.13; F, 3.36; N, 9.91; S, 5.67; Found: C, 55.25; H, 3.23; Br, 14.15; F, 3.38; N, 9.93; S, 5.69%.

##### 5-(4-bromophenyl)-N’-(3-(4-fluorophenyl)-4-p-tolylthiazol-2(3H)-ylidene)isoxazole-3-carbohydrazide (**11i**)

Beige solid; yield: 93%; m.p.: 120.3–122.3 °C; IR (KBr, cm^−1^): 3121, 2921, 1696, 1607, 1502, 1443, 1228; ^1^H NMR (300 MHz, DMSO) δ: 11.06 (1H, -NH), 7.87–6.94 (12H, Ar-H), 6.52 (1H, oxazole ring proton), 6.42 (1H, thiazole ring proton), 2.27–2.22 (3H, -CH_3_); ^13^C NMR (75 MHz, DMSO) δ: 170.0, 168.9, 159.2, 154.9, 151.8, 146.3, 143.6, 143.1, 140.6, 133.0, 130.7, 130.5, 129.8, 129.6, 128.8, 128.4, 128.1, 126.2, 122.7, 118.5, 115.4, 114.8, 110.0, 101.2, 21.4; Anal. Calcd for C_26_H_18_BrFN_4_O_2_S: C, 56.84; H, 3.30; Br, 14.54; F, 3.46; N, 10.20; S, 5.84; Found: C, 56.86; H, 3.33; Br, 14.51; F, 3.48; N, 10.23; S, 5.82%.

##### 5-(4-bromophenyl)-N’-(3-(4-fluorophenyl)-4-(4-methoxyphenyl)thiazol-2(3H)-ylidene)isoxazole-3-carbohydrazide (**11j**)

Beige solid; yield: 98%; m.p.: 118.5–120.5 °C; IR (KBr, cm^−1^): 3121, 2973, 2895, 1676, 1606, 1506, 1445, 1251; ^1^H NMR (300 MHz, DMSO) δ: 10.97 (1H, -NH), 7.88–6.81 (12H, Ar-H), 6.41 (1H, oxazole ring proton), 6.32 (1H, thiazole ring proton), 3.73–3.68 (3H, -OMe); ^13^C NMR (75 MHz, DMSO) δ:171.8, 162.4, 143.5, 133.0, 130.6, 130.4, 129.73, 128.5, 115.4, 114.8, 114.7, 107.7, 56.3; Anal. Calcd for C_26_H_18_BrFN_4_O_3_S: C, 55.23; H, 3.21; Br, 14.13; F, 3.36; N, 9.91; S, 5.67; Found: C, 55.25; H, 3.23; Br, 14.15; F, 3.38; N, 9.93; S, 5.69%.

##### 5-(3,4-bis(4-fluorophenyl)thiazol-2(3H)-ylidene)-5-(4-bromophenyl)isoxazole-3-carbohydrazide (**11k**)

Beige solid; yield: 98%; m.p.: 105.3–107.3 °C; IR (KBr, cm−^1^): 3117, 2921, 2838, 1676, 1604, 1503, 1444, 1225; ^1^H NMR (300 MHz, DMSO) δ: 10.97 (1H, -NH), 7.88–6.78 (12H, Ar-H), 6.41 (1H, oxazole ring proton), 6.32 (1H, thiazole ring proton);^13^C NMR (75 MHz, DMSO) δ: 169.8, 169.4, 166.4, 163.8, 161.8, 161.3, 159.8, 159.3, 159.2, 154.9, 153.1, 150.6, 147.6, 139.1, 133.0, 131.4, 130.6, 130.5, 128.5, 116.2, 115.9, 114.9; Anal. Calcd for C_25_H_15_BrF_2_N_4_O_2_S: C, 54.26; H, 2.73; Br, 14.44; F, 6.87; N, 10.12; S, 5.79; Found: C, 54.28; H, 2.75; Br, 14.46; F, 6.88; N, 10.15; S, 5.81%.

##### 5-(4-bromophenyl)-N’-(3-(4-chlorophenyl)-4-p-tolylthiazol-2(3H)-ylidene)isoxazole-3-carbohydrazide (**11l**)

Beige solid; yield: 97%; m.p.: 127.3–129.3 °C; IR (KBr, cm^−1^): 3122, 2934, 2920, 1693, 1608, 1486, 1445, 1227; ^1^H NMR (300 MHz, DMSO) δ: 10.97 (1H, -NH), 7.76–6.93 (12H, Ar-H), 6.49 (1H, oxazole ring proton), 6.37 (1H, thiazole ring proton), 2.27–2.20 (3H, -CH_3_); ^13^C NMR (75 MHz, DMSO) δ:170.0, 168.9, 159.1, 154.9, 151.8, 146.3, 143.6, 143.1, 140.6, 133.0, 130.7, 130.5, 129.8, 129.6, 128.8, 128.4, 128.1, 126.2, 122.7, 118.5, 115.4, 114.8, 110.0, 101.2, 21.4; Anal. Calcd for C_26_H_18_BrClN_4_O_2_S: C, 55.19; H, 3.21; Br, 14.12; Cl, 6.27; N, 9.90; S, 5.67; Found: C, 55.21; H, 3.23; Br, 14.14; Cl, 6.25; N, 9.93; S, 5.70%.

##### 5-(4-bromophenyl)-N’-(3-(4-chlorophenyl)-4-(4-methoxyphenyl)thiazol-2(3H)-ylidene)isoxazole-3-carbohydrazide (**11m**)

Beige solid; yield: 95%; m.p.: 120.5–122.5 °C; IR (KBr, cm^−1^): 3116, 2935, 2835,1671, 1606, 1508, 1446, 1251; ^1^H NMR (300 MHz, DMSO) δ: 11.00 (1H, -NH), 7.87–6.76 (12H, Ar-H), 6.43 (1H, oxazole ring proton), 6.30 (1H, thiazole ring proton), 3.73–3.67 (3H, -OMe); ^13^C NMR (75 MHz, DMSO) δ: 170.7, 161.7, 159.2, 158.1, 150.8, 137.9, 133.5, 133.0, 131.2, 130.6, 130.4, 130.2, 129.7, 129.4, 128.5, 128.4, 121.5, 116.4, 116.1, 101.3, 50.4; Anal. Calcd for C_26_H_18_BrClN_4_O_3_S: C, 53.67; H, 3.12; Br, 13.73; Cl, 6.09; N, 9.63; S, 5.51; Found: C, 53.69; H, 3.14; Br, 13.75; Cl, 6.10; N, 9.65; S, 5.54%.

##### 5-(4-bromophenyl)-N’-(3-(4-chlorophenyl)-4-(4-fluorophenyl)thiazol-2(3H)-ylidene)isoxazole-3-carbohydrazide (**11n**)

Beige solid; yield: 98%; m.p.: 128.3–130.3 °C; IR (KBr, cm^−1^): 3117, 2917, 1689, 1605, 1505, 1445, 1227; ^1^H NMR (300 MHz, DMSO) δ: 11.04 (1H, -NH), 7.88–6.93 (12H, Ar-H), 6.56 (1H, oxazole ring proton), 6.44 (1H, thiazole ring proton); ^13^C NMR (75 MHz, DMSO) δ: 170.0, 169.3, 167.4, 162.4, 160.0, 159.6, 159.1, 158.6, 147.7, 142.3, 139.1, 136.8, 133.1, 130.4, 129.7, 128.5, 125.0, 123.5, 122.7, 121.5, 121.3, 115.4, 114.7; Anal. Calcd for C_25_H_15_BrClFN_4_O_2_S: C, 52.69; H, 2.65; Br, 14.02; Cl, 6.22; F, 3.33; N, 9.83; S, 5.63; Found: C, 52.71; H, 2.67; Br, 14.05; Cl, 6.25; F, 3.34; N, 9.85; S, 5.65%.

### 2.2. Biologic Activity Studies

#### 2.2.1. Purification of Carbonic Anhydrase I and II from Human Blood Red Cells

The purification process began with the collection of 25 mL blood samples from a single healthy adult volunteer. Written informed consent was obtained prior to participation. The samples were centrifuged at 1000× *g* for 20 min at 4°C, after which the supernatant was removed. The packed erythrocytes were then subjected to three washes with 0.9% NaCl and subsequently haemolysed in cold water. Adjustment of the haemolysate’s pH to 8.5 was accomplished with a solid Tris-base. Additionally, 25 mL of the haemolysate was applied to an affinity column that contained Sepharose 4B-ethylene diamine-4-isothiocyanate-benzenesulfonamide [33]. The recoveries of hCA I and II were achieved through elution with 0.1 M NaCl/25 mM Na_2_HPO_4_ (pH 6.3) and 0.1 M CH_3_COONa/0.5 M NaClO_4_ (pH 5.6), respectively.

#### 2.2.2. Esterase and Hydratase Activity Assay

To determine the enzymatic activity of carbonic anhydrase (CA), two different catalytic functions were evaluated. The enzyme’s esterase activity was quantified by observing the breakdown of p-nitrophenyl acetate. The rate of 4-nitrophenylate ion formation was monitored by measuring the absorbance at 348 nm for three minutes at 25 °C using a BioTek automated recording spectrophotometer. To ensure reproducibility, all assays were performed in triplicate [34]. The enzyme’s CO_2_-hydratase activity, a more physiologically relevant function, was calculated using the formula (t0 − tc/tc). This equation compares the time it takes for a pH change to occur without the enzyme (t0) versus with the enzyme (tc), yielding a value in enzyme units (EU) [35]. To verify that the observed activity was specific to carbonic anhydrase, acetazolamide (AAZ), a non-selective inhibitor of CA, was used as a standard.

#### 2.2.3. In Vitro Inhibition Studies

This study investigated how effectively 15 newly synthesised isoxazole-fused thiazole–oxazole derivatives (labelled **11a**–**n**) could inhibit two human carbonic anhydrase isozymes, hCA I and hCA II. The IC50 value—the concentration of an inhibitor that causes a 50% loss of enzyme activity—was obtained by plotting the inhibitor concentration against the remaining enzyme activity. To this end, the impact of increasing concentrations of compounds 11a-n on both the hydratase and esterase functions of the enzymes was measured. The inhibitory effects of the compounds on enzyme activities were tested under in vitro conditions; the Ki values given in Table 1 were calculated from Lineweaver–Burk graphs by using five substrate concentrations ranging from 0.5 to 1.4 mM as a control and two different inhibitor concentrations indicated in the graphs.

### 2.3. Molecular Docking Studies

#### 2.3.1. Computational Methods

The crystal structures of carbonic anhydrase I (CA I, PDB ID: 1BZM) [32,36,37] and carbonic anhydrase II (CA II, PDB ID: 4FU5), co-crystallised with sulfonamide-based native ligands MZM and N-[(2Z)-1,3-oxazolidin-2-ylidene] sulfuric diamide (0VX), respectively, were retrieved from the Protein Data Bank. Both protein–ligand complexes were prepared for in silico calculations using the Flare™ v9.0.0 drug discovery software suite (Cresset, UK). Default settings were employed for protein preparation. The bound native ligands (MZM for CA I and 0VX for CA II) were used to define the centre of the binding site for each enzyme. Molecular docking was performed using the “very accurate but slow” protocol provided in Flare™ to ensure high-quality binding mode predictions.

Validation of the docking protocol was performed by redocking the co-crystallised ligands (MZM for CA I and 0VX for CA II) into their respective active sites. The root-mean-square deviation (RMSD) values between the docked and crystallographic poses were calculated as 0.570 Å for CA I (PDB ID: 1BZM) and 1.429 Å for CA II (PDB ID: 4FU5), both of which are below the commonly accepted threshold of 2.0 Å, confirming the reliability of the docking procedure. The superimposition of the docked and native ligand poses is provided in the Supplementary Data (Supplementary S1–S3).

Furthermore, the MM/GBSA protocol was carried out in the same package programme using the default setting [38,39,40].

#### 2.3.2. Molecular Dynamics (MD) Simulations

Molecular dynamics (MD) simulations were performed using Flare™ v9.0.0 software (Cresset, UK), which incorporates the OpenMM engine. An orthorhombic solvent box was constructed around each protein–ligand complex using an explicit water model. For the CA I system, the three-point extended simple point charge (SPC/E) water model was employed, whereas the CA II system was solvated using the TIP4P/2005 water model. In both cases, the ionic strength was adjusted to 0.15 M NaCl to mimic physiological conditions. The AMBER force field was used to parameterise the systems. Periodic boundary conditions were applied, and long-range electrostatic interactions were treated using the Particle Mesh Ewald (PME) method. The reporting interval was set to every 20,000 time steps. Each system was simulated for 300 ns under an isothermal–isobaric ensemble (NPT), maintaining constant pressure and temperature throughout the production phase.

## 3. Results and Discussion

### 3.1. Chemistry

**Scheme 2:**
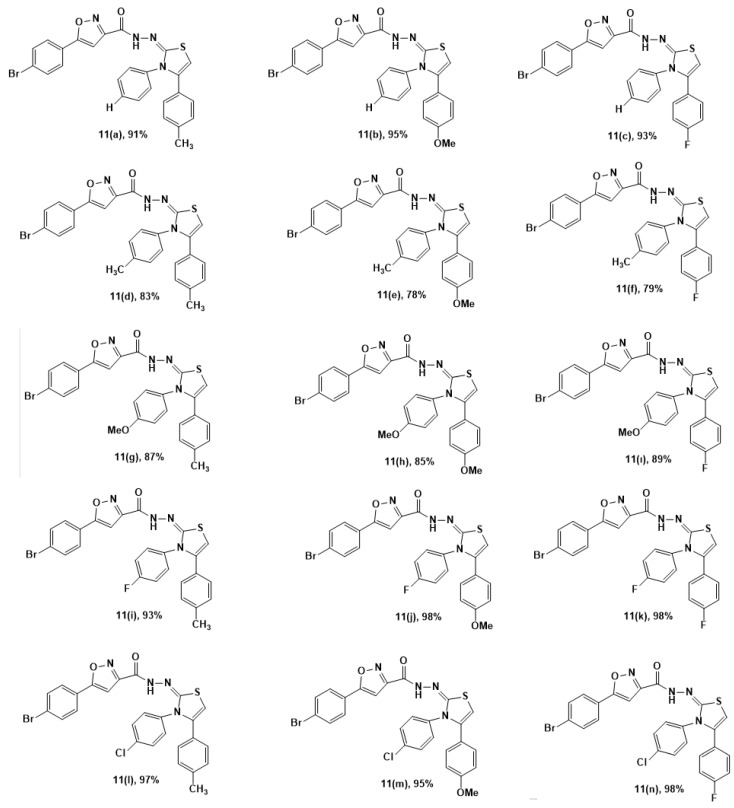
The structures and yields of all synthesised compounds.

The synthesis of thiazole derivatives (**11a–n**) was carried out in five steps. In the first step, 4-bromoacetophenone (1mmol) and diethyl oxalate (1mmol) were treated in an ethanolic solution of sodium ethoxide for 24 h, giving β-ketoester (**3**) [25]. In the second step, hydroxyl ammonium chloride (1.25 mmol) was reacted, and it was first stirred for 2 h at room temperature with β-ketoester (**3**) (1 mmol) and then refluxed for 5 h in ethanol to obtain isoxazole ester (**5**). In the third step, hydrazine hydrate (1.5 mmol) was reacted and refluxed with isoxazole ester (**5**) (1 mmol) for 24 h in ethanol to obtain 5-(4-bromophenyl) isoxazole-3-carbohydrazide (**7**) [30]. In the fourth step, various substituted isothiocyanate compounds (1mmol) were treated with 5-(4-bromophenyl) isoxazole-3-carbohydrazide (**7**) (1mmol) in THF in the presence of Et_3_N for 24 h to obtain thiourea derivatives (**9a**–**e**) [31]. Finally, three phenacyl bromide derivatives (1 mmol) were treated with thiourea derivatives (**9a**–**e**) (1 mmol) in EtOH/DMF (5:5 *v*/*v*) for 24 h to obtain thiazole derivatives (**11a**–**n**). The structures and yields of all synthesised compounds are shown in Scheme 2. IR, NMR (^1^H and ^13^C), and elemental analysis were carried out to identify the prepared compounds.

The structural framework of the target compounds **11**(**a–n**) was confirmed through a detailed spectroscopic analysis. In the ^1^H NMR spectra, the emergence of characteristic N–H proton resonances at 11.00 ppm provided primary evidence for the successful formation of the amide linkage. The presence of isoxazole and thiazole rings was identified by their specific proton signals at approximately 6.50 ppm and 6.40 ppm, respectively. Furthermore, ^13^C NMR data supported the backbone integrity, with distinctive carbonyl (C=N) and thiazole carbon signals appearing at 160 ppm and 100 ppm. The results, complemented by FT-IR stretching frequencies for (N–H) and (C=N) groups, are in full agreement with the proposed molecular structures. The structural framework of the target compounds **11**(**a**–**n**) was confirmed through a detailed spectroscopic analysis. In the ^1^H NMR spectra, the N–H proton resonance at around 11.00 ppm and the –CH proton peaks on the aromatic ring were between 7.94 and 6.77 ppm; the isoxazole and thiazole ring protons produced peaks around 6.50 ppm and 6.40 ppm, respectively. Also, it was observed that the proton peaks of -OMe and -CH_3_ in the target compounds **11**(**a**–**n**) occurred at 3.73–3.35 ppm and 2.27–2.12 ppm, respectively [32]. The carbonyl group signals (C=N) and (=C) carbon atoms of the thiazole ring were observed in the ^13^C NMR spectra at about 160 and 100 ppm [41]. It is known that, in the infrared spectra of the target compounds **11**(**a**–**n**), (N–H) (NH–C=O), (C═N), and (C–O/N–O isoxazole ring) peaks occur around 3087, 1608, 1504, and 1227 ppm, respectively [42]. The synthesised compounds’ structures are supported by all of the spectra and elemental analyses.

### 3.2. CA Activity Assay

The inhibitory activities of the newly synthesised compounds against human carbonic anhydrase I (hCA I) and II (hCA II) were investigated. hCA I and hCA II isozymes were purified from human erythrocytes by affinity chromatography. The inhibitor concentrations causing 50% inhibition (IC50 values) were determined from regression analysis graphs for the novel derivatives. The IC50 values of the compounds are presented in Table 1.

The hCA I hydratase and esterase activities were inhibited by the novel synthesised thiazole–oxazole derivatives, with IC_50_ values ranging from 0.039 to 0.233 mM and 0.125 to 0.865 mM, respectively. Also, all studied compounds showed inhibitory activity against hCA II hydratase and esterase, with IC_50_ values ranging from 0.032 to 0.179 mM and 0.106 to 0.763 mM, respectively.

Among them, compound **11d** demonstrated the strongest inhibition for all isoenzymes, with IC50 values of 0.039 mM (hCA I) and 0.032 mM (hCA II) for hydratase activity and 0.125 mM (hCA I) and 0.106 mM (hCA II) for esterase activity. For both activities, the weakest inhibitor was **11b**, with IC50 values of 0.23 mM (hCA I) and 0.179 mM (hCA II), respectively. According to esterase activity, the weakest inhibitor was the **11j** compound with an IC_50_ value of 0.865 mM for hCA I, while the weakest inhibitor was the **11a** compound with an IC_50_ value of 0.763 mM for hCA II.

Among the synthesised compounds, compound **11d** may have shown a stronger inhibitory effect due to the two methyl groups that it contains. The methyl group (–CH_3_) fits well with the hydrophobic pockets in the binding region due to its small and hydrophobic structure. At the same time, it does not prevent the formation of π–π stacking interactions and can even support them. The methoxy group in **11j** is a bulkier and polar group, which may make it difficult for it to fit into the binding pockets of the enzyme due to steric or electrostatic reasons. Since **11a** contains H, its electrostatic effect may be low, therefore causing it to remain neutral in terms of interactions and possibly preventing strong binding modes.

### 3.3. Computational Studies

#### 3.3.1. Molecular Docking and ADME Predictions

In drug discovery, ADME prediction is crucial for understanding the pharmacokinetics and pharmacodynamics. Therefore, initially, the ADME was predicted and evaluated for all synthesised compounds (**11a**–**n**).

According to the ADME profile, as shown in Table 2, the synthesised compounds exhibit relatively low polar surface areas (TPSA < 80 Å^2^), together with pronounced lipophilicity (SlogP = 6.2–7.3). This physicochemical profile is generally associated with enhanced passive membrane permeability, which is a critical factor for intracellular targets such as human carbonic anhydrase I and II (hCA I and hCA II), both of which are cytosolic enzymes [43,44]. In this context, increased lipophilicity may facilitate cellular uptake and improve access to the active site of these isoforms, supporting the likelihood that the synthesised compounds can effectively reach CA I and CA II at the cellular level.

From a central nervous system (CNS) perspective, the relatively low TPSA values are also consistent with properties commonly associated with blood–brain barrier (BBB) permeability [45]. Moreover, carbonic anhydrase isoforms, particularly CA II, are widely expressed in the brain and have been implicated in several neurodegenerative and neurological conditions, including epilepsy, cerebral ischemia, and neuroinflammation, making BBB penetration a desirable feature for CA-targeting agents [46]. However, despite favourable polarity, the relatively high molecular weights (530–581 Da) and elevated lipophilicity (logP > 6) of the compounds exceed the typical optimal ranges for CNS drugs, which may limit their efficient brain penetration. Therefore, while the compounds appear suitable for reaching intracellular CA I and CA II, their BBB permeability cannot be conclusively predicted based solely on these parameters and should be further validated using dedicated experimental or computational models.

Although molecular docking and MM-GBSA calculations are widely employed to estimate ligand–protein binding affinity, it is well established that these approaches are not sufficiently reliable for directly ranking compounds based on biological activity or for distinguishing active from inactive molecules with high accuracy. Docking scores, in particular, are primarily useful for predicting plausible binding poses and identifying key interactions within the active site, rather than quantitatively correlating with experimental IC_50_ or K_i_ values [47,48]. Similarly, although MM-GBSA provides a more refined estimation of binding free energy, it still involves approximations that limit its predictive power for precise activity ranking, especially in structurally similar compound series [49].

In line with this understanding, the present study did not rely on docking scores or MM-GBSA values to establish an activity order among the synthesised compounds. Instead, the computational analysis was focused on identifying key ligand–protein interactions, including hydrogen bonding, π–π stacking, and hydrophobic contacts within the catalytic site of hCA I and hCA II. Following the identification of the most plausible binding conformations through docking, molecular dynamics (MD) simulations were performed to evaluate the stability of the ligand–protein complexes under dynamic conditions. This approach enables the investigation of time-dependent interaction patterns and provides a more realistic representation of binding behaviour than static docking models.

#### 3.3.2. MD Simulations

The in vitro enzyme inhibition assays included both hydratase and esterase activities. However, our interpretation focused primarily on hydratase activity, which is more physiologically relevant and widely accepted as a more accurate reflection of CA function [50]. This choice is also supported by literature reports suggesting that esterase-based assays may yield false positives. Additionally, the 300 ns duration of the simulations was chosen to explore whether a Zn^2+^ coordination interaction might emerge over a longer timeframe. Nevertheless, no such coordination was observed in either CA I or CA II simulations, which aligns with the experimental finding that the compound exhibits stronger activity than acetazolamide despite lacking direct metal coordination.

To ensure methodological consistency with our previous study [32] and to rely on a well-validated structural model, we selected the crystal structure with PDB ID: 1BZM for CA I. This structure has been widely used in the literature for CA I–ligand interaction studies and provides a reliable framework for molecular docking and dynamics simulations.

**Figure 2:**
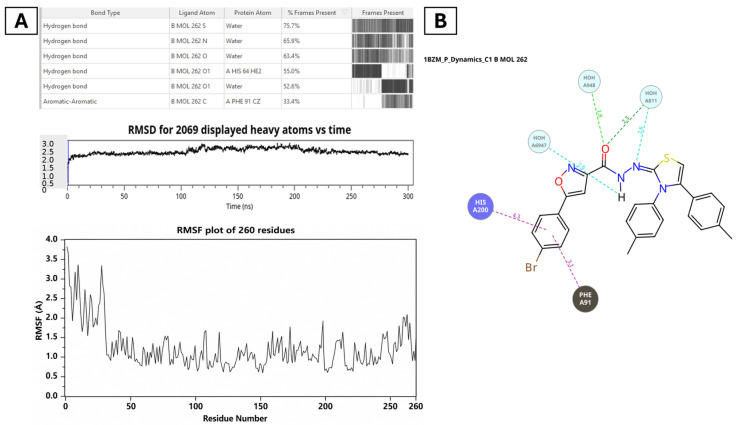
Key molecular interactions and dynamic stability of the most active compound in the CAI active site (PDB ID: 1BZM). (**A**) Clustering table illustrating the interactions, RMSD plot showing complex stability over 300 ns, and RMSF profile indicating localised flexibility near the binding site. No Zn^2+^ coordination was observed during the simulation. (**B**) 2D ligand–residue interactions.

The RMSD plot of the CAI (PDB ID: 1BZM) backbone throughout the 300 ns simulation indicated overall structural stability of the complex, with deviations remaining below 3 Å for the majority of the simulation, as can be seen in Figure 2. A slight increase in flexibility was observed between 100 and 200 ns, which coincided with the temporary loss of a hydrogen bond between the compound and His64, a residue known for its role in internal proton shuttling. However, this fluctuation did not cause any significant destabilisation, and the system regained equilibrium after 200 ns.

The RMSF analysis further supported this observation, revealing increased flexibility in the loop regions near the binding site, which is typical in such simulations, as shown in Figure 2. Importantly, there were no major conformational changes that could compromise ligand binding, suggesting that the binding mode remained conserved throughout the trajectory.

A detailed interaction analysis of the most active compound **11d** revealed T-shaped π–π stacking interactions with Phe91 and His200 [32,51] through its 4-bromophenyl moiety (Figure 2). These residues have previously been reported as critical for CAI inhibition [36]. Additionally, a water-mediated hydrogen bond was observed between the ligand and Tyr204, further stabilising the complex. These interaction patterns are consistent with the experimental inhibition results, where compound **11d** exhibited the lowest IC_50_ values among the series.

Interestingly, although no direct interaction with the catalytic Zn^2+^ ion was observed, an interaction generally associated with enhanced inhibitory activity, the compound exhibited approximately five-fold stronger activity than acetazolamide (AAZ) in vitro. This result suggests that strong interactions with key residues, such as Phe91, His200, and Tyr204, may compensate for the absence of direct Zn coordination. This interpretation is consistent with the experimental findings, where compound 11d exhibited the lowest IC_50_ values within the series. However, it should be noted that the IC_50_ values remained in the millimolar range, whereas compounds that directly coordinate with Zn^2+^ typically display higher potency in the micromolar range. Taken together, these findings indicate that, while Zn^2+^ coordination is not strictly required for activity, it likely contributes significantly to enhanced inhibitory potency.

These findings provide valuable insight into the structural and dynamic behaviour of the compound within the CAI active site and underline the importance of both canonical Zn^2+^ coordination and alternative stabilising interactions in modulating inhibitor activity.

For the MD simulation studies of CAII, we selected the protein structure with PDB ID 4FU5, originally resolved from *E. coli* and published in 2012 [37]. This choice was based on its improved crystallographic resolution and lower R-values compared to previously used structures such as 1AVM. Our group previously employed PDB ID 4FU5 in a related study, and, to maintain methodological consistency, we adopted the same structure here. Docking was performed using the same parameters as in that study, with the grid box centred on the native ligand (0VX) to define the active binding site.

**Figure 3:**
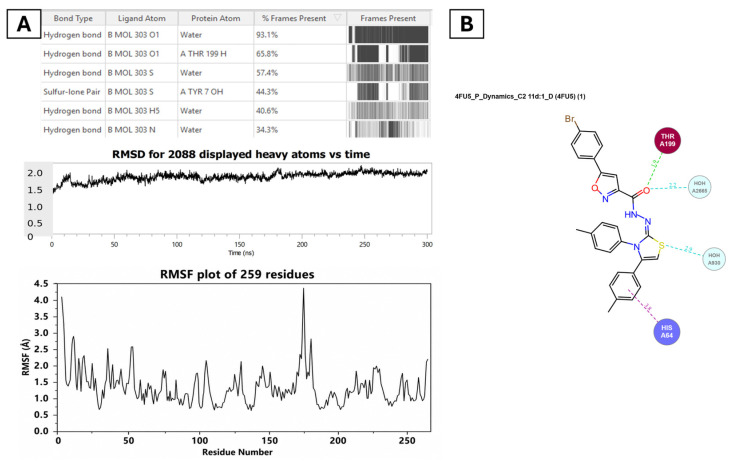
Key molecular interactions and dynamic stability of the most active compound in the CAII active site (PDB ID: 4FU5). (**A**) Clustering table illustrating the interactions, RMSD plot showing complex stability over 300 ns, and RMSF profile indicating localised flexibility near the binding site. (**B**) 2D ligand–residue interactions.

Subsequently, a 300 ns MD simulation was conducted using the explicit TIP4P/2005 water model to assess the binding dynamics of compound **11d**. As illustrated in Figure 3, compound **11d** is well positioned within the binding cavity, which comprises TRP5, TYR7, VAL121, LEU141, VAL143, LEU198, PRO201, PRO202, VAL207, and TRP209 [50], establishing key interactions with amino acids previously reported to be critical for CA II inhibition. The binding mode involves hydrogen bonds with THR199 and THR200, a T-shaped π-π interaction with HIS64, and a sulfur lone pair interaction with TYR7, as can be seen in Figure 3.

**Figure 4:**
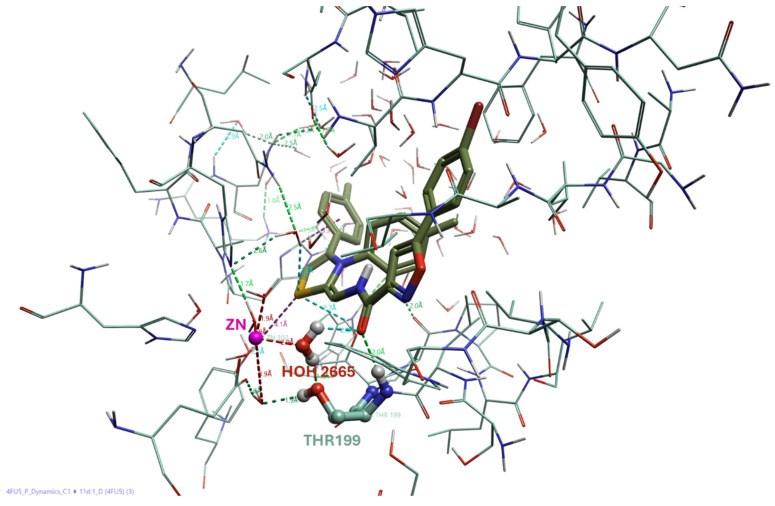
Water-mediated interaction between compound **11d** and the catalytic Zn^2+^ ion within the CAII (PDB ID: 4FU5) active site.

Moreover, water-mediated hydrogen bonds were observed with ASN67 and HIS94, as well as an indirect interaction with the Zn^2+^ ion via HOH2665. This indirect Zn^2+^ interaction is particularly significant, as previous studies have emphasised the critical role of zinc coordination in potent CA II inhibition (Figure 4) [37,52,53].

The enhanced inhibitory potency of compound **11d** against CAII—compared to its activity against CAI—is believed to stem from its ability to interact, even indirectly, with the catalytic Zn^2+^ ion through water-mediated hydrogen bonding. This interaction, particularly observed via HOH2665, may significantly contribute to its stronger inhibition profile, as can be seen in Figure 4. Previous studies have consistently emphasised the importance of direct or water-bridged interactions with the Zn^2+^ ion in strengthening carbonic anhydrase inhibition [53]. Thus, the interaction observed in Figure 4 aligns well with the in vitro findings, where compound **11d** exhibited greater CAII inhibitory activity than acetazolamide.

While the observed Zn^2+^ interaction occurs via water mediation, the absence of direct coordination suggests a potential area for optimisation. Future design efforts may focus on introducing functional groups capable of establishing stronger or direct interactions with the Zn^2+^ ion, such as sulfonamide or hydroxamate moieties, within the isoxazole-containing thiazole scaffold, to further improve the inhibitory efficacy.

### 3.4. Structure–Activity Relationship (SAR) Analysis of hCAI and hCAII Inhibitors

The combined evaluation of the in vitro and in silico results reveals a consistent activity trend across both hCAI and hCAII, with compound **11d** identified as the most potent inhibitor, whereas **11a** and **11b** can be considered weakly active or nearly inactive. Compounds such as **11e**, **11l**, and **11m** exhibit moderate activity, indicating a well-defined structure–activity pattern within the series (Table 1).

The SAR analysis can be summarised as follows:

- Para-methyl substitution on the phenyl ring attached to the thiazole nitrogen significantly enhances activity. This modification, as observed in compound **11d**, leads to a sharp increase in inhibitory potency against both CA I and CA II.
- Replacement of the para-methyl group with a methoxy substituent on the second phenyl ring (attached to the C4 position of the thiazole) results in a slight decrease in activity. This suggests that, while electron-donating groups are generally favourable, subtle differences in steric and electronic properties influence binding efficiency.
- Removal of the para-methyl group or its substitution with halogens on the N-linked phenyl ring drastically reduces activity. This indicates that both the presence and the electronic nature of the substituent at this position are critical for maintaining inhibitory potency.
- The hydrazide linker plays a crucial role in activity by stabilising ligand binding through hydrogen bonding and conformational control. This moiety appears to contribute to maintaining an optimal binding orientation within the active site, indirectly enhancing inhibitory potency.
- The thiazole sulfur contributes to binding via water-mediated interaction with the catalytic Zn^2+^ ion. Although not directly coordinating the metal centre, this interaction may support binding affinity, and bioisosteric replacement of sulfur (S→O or N) could modulate metal interaction and influence activity.

Interestingly, molecular dynamics (MD) simulations reveal that these substituents do not directly participate in stable interactions with the active site residues of either hCA I or hCA II. Despite this, their impact on biological activity is pronounced. This apparent discrepancy suggests that these groups may influence activity through indirect effects, such as modulating ligand conformation, optimising the overall binding orientation, or enhancing hydrophobic complementarity within the active site rather than forming direct interactions. Such behaviour is not uncommon in enzyme–ligand systems, where peripheral substituents contribute to binding affinity by stabilising the ligand in a favourable conformation.

**Figure 5:**
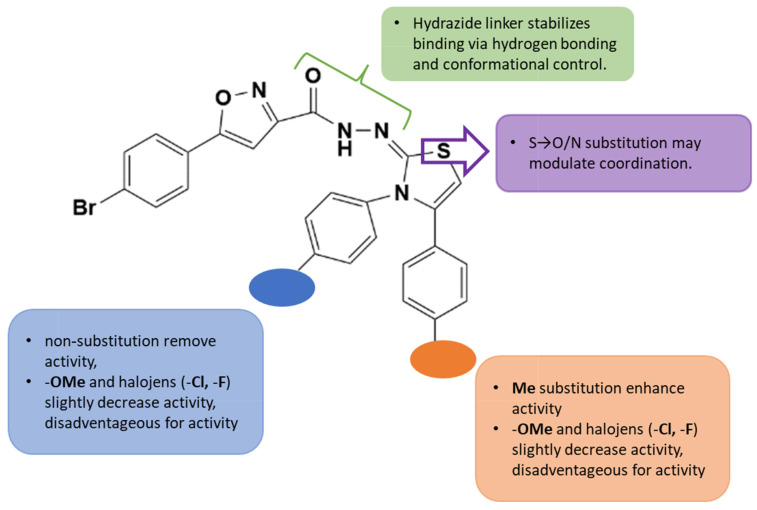
Summary of the structure–activity relationship (SAR) of the synthesised isoxazole–thiazole derivatives against hCA I and hCA II.

These observations are schematically illustrated in Figure 5, highlighting the key substituent effects governing the activity profile of the series.

## Figures and Tables

**Table 1:** IC50 and Ki values of compounds for hCA I and hCA II.

Compounds	hCA I			hCA II		
	IC_50_(mM) (Hydratase)	IC_50_(mM) (Esterase)	Ki(mM) (Esterase)	IC_50_(mM) (Hydratase)	IC_50_(mM) (Esterase)	Ki(mM) (Esterase)
**11a**	0.199	0.641	0.179	0.153	0.763	0.117
**11b**	0.233	0.552	0.134	0.179	0.374	0.175
**11c**	0.114	0.266	0.074	0.056	0.191	0.053
**11d**	0.039	0.125	0.033	0.032	0.106	0.029
**11e**	0.107	0.234	0.076	0.041	0.497	0.033
**11f**	0.110	0.258	0.071	0.068	0.308	0.065
**11g**	0.152	0.634	0.132	0.079	0.379	0.051
**11h**	0.160	0.491	0.104	0.078	0.385	0.062
**11ı**	0.139	0.292	0.136	0.051	0.216	0.050
**11i**	0.133	0.829	0.095	0.066	0.272	0.063
**11j**	0.197	0.865	0.120	0.088	0.422	0.081
**11k**	0.159	0.425	0.126	0.091	0.383	0.078
**11l**	0.106	0.254	0.060	0.041	0.168	0.033
**11m**	0.098	0.240	0.069	0.051	0.222	0.031
**11n**	0.116	0.447	0.109	0.079	0.402	0.065
Acetazolamide	0.018	0.260	0.130	0.010	0.220	0.110

**Table 2:** Predicted ADME properties, molecular docking scores, and MM-GBSA binding free energy values of the synthesised compounds against hCA I (PDB ID: 1BZM) and hCA II (PDB ID: 4FU5). MW: molecular weight (g/mol) (recommended value <500), PSA: polar surface area (Å) (recommended value ≤140 Å), LogP: logarithm of the octanol/water ratio coefficient of the compound (recommended value <5). LF VSscore: docking score (kcal/mol), MM/GBSA dG binding energy (kcal/mol).

Title	Protein	MW	SlogP	TPSA	Flex.	#RB	HBA	HBD	Rof5	LF VSscore	MM/GBSA dG	
											1BZM	4FU5
**11a**	1BZM_P	530.4	6.5	58.4	0.8	5	5	1	2	−9.546	−23.66	−27.36
**11b**	1BZM_P	546.4	6.2	67.6	1.3	6	6	1	2	−10.592	−31.09	−27.71
**11c**	1BZM_P	534.4	6.6	58.4	0.8	5	5	1	2	−9.522	−27.55	−27.69
**11d**	1BZM_P	544.5	6.9	58.4	0.8	5	5	1	2	−9.352	−26.73	−29.00
**11e**	1BZM_P	560.5	6.6	67.6	1.3	6	6	1	2	−9.107	−26.59	−28.69
**11f**	1BZM_P	548.4	7.0	58.4	0.8	5	5	1	2	−9.401	−25.72	−25.92
**11g**	1BZM_P	560.5	6.6	67.6	1.3	6	6	1	2	−9.410	−28.74	−30.19
**11h**	1BZM_P	576.5	6.3	76.8	1.8	7	7	1	2	−11.328	−35.62	−29.21
**11i**	1BZM_P	564.4	6.7	67.6	1.3	6	6	1	2	−11.029	−32.19	−28.27
**11ı**	1BZM_P	548.4	7.0	58.4	0.8	5	5	1	2	−10.090	−26.60	−27.73
**11j**	1BZM_P	564.4	6.7	67.6	1.3	6	6	1	2	−11.405	−34.25	−30.30
**11k**	1BZM_P	552.4	7.1	58.4	0.8	5	5	1	2	−9.926	−30.36	−25.16
**11l**	1BZM_P	564.9	7.2	58.4	0.8	5	5	1	2	−9.832	−22.37	−28.76
**11m**	1BZM_P	580.9	6.9	67.6	1.3	6	6	1	2	−11.540	−32.58	−29.41
**11n**	1BZM_P	568.8	7.3	58.4	0.8	5	5	1	2	−9.942	−30.45	−26.53

## Data Availability

The data are contained within the article and Supplementary Materials.

## Supplementary Materials

The following supporting information can be downloaded at: https://www.mdpi.com/article/10.3390/molecules31111824/s1, Supplementary S1: NMR spectra; Supplementary S2: IR spectra; Supplementary S3: Inhibition graphics.
