# Supplementary material for: Isoxazole–Thiazole Hybrids: Synthesis, Structural Characterisation, Carbonic Anhydrase Inhibition, and Molecular Docking Studies
Source: Molecules. 2026 May 25;31(11):1824. doi: 10.3390/molecules31111824 (PMC13258222; doi:10.3390/molecules31111824)
Supplement: Supplementary file 1 [file molecules-31-01824-s001.zip › molecules-4277983-supplementary.pdf]

Scheme 1: NMR spectra

11a) 5-(4-bromofenil)-(3-(4-florofenil)-4-(p-tolil)tiazol-2(3H)-iliden)izoksazol-3-karbohidrazit

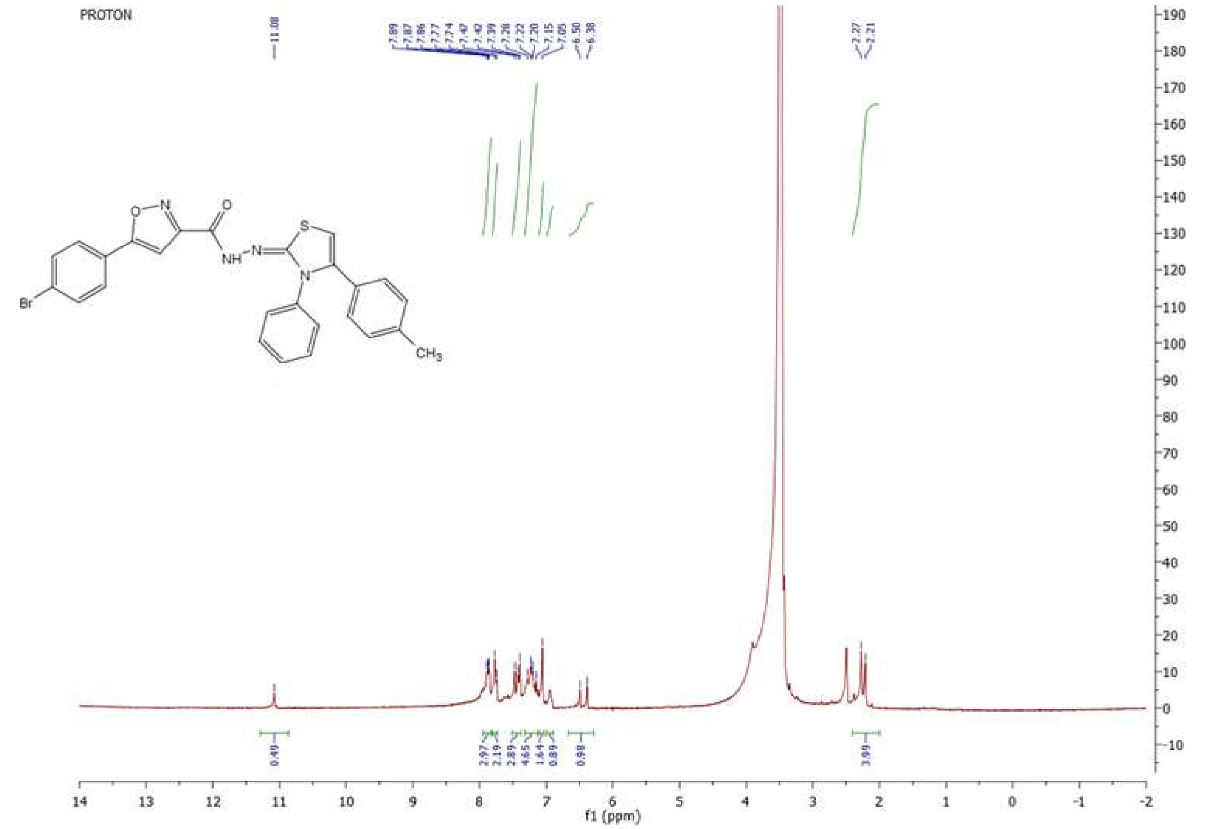

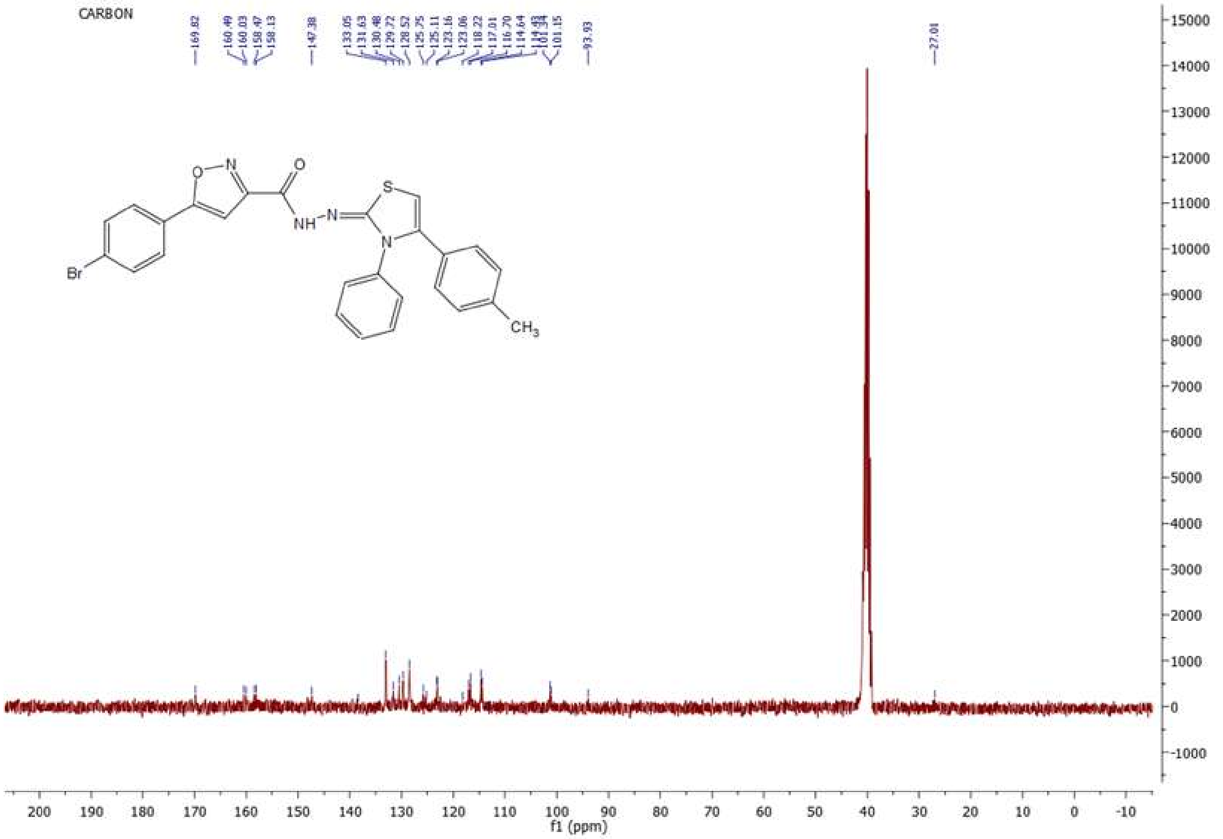

11b) 5-(4-bromofenil)-N'-(4-(4-metoksifenil)-3-feniltiazol-2(3H)-iliden)izoksazol-3-karbohidrazid

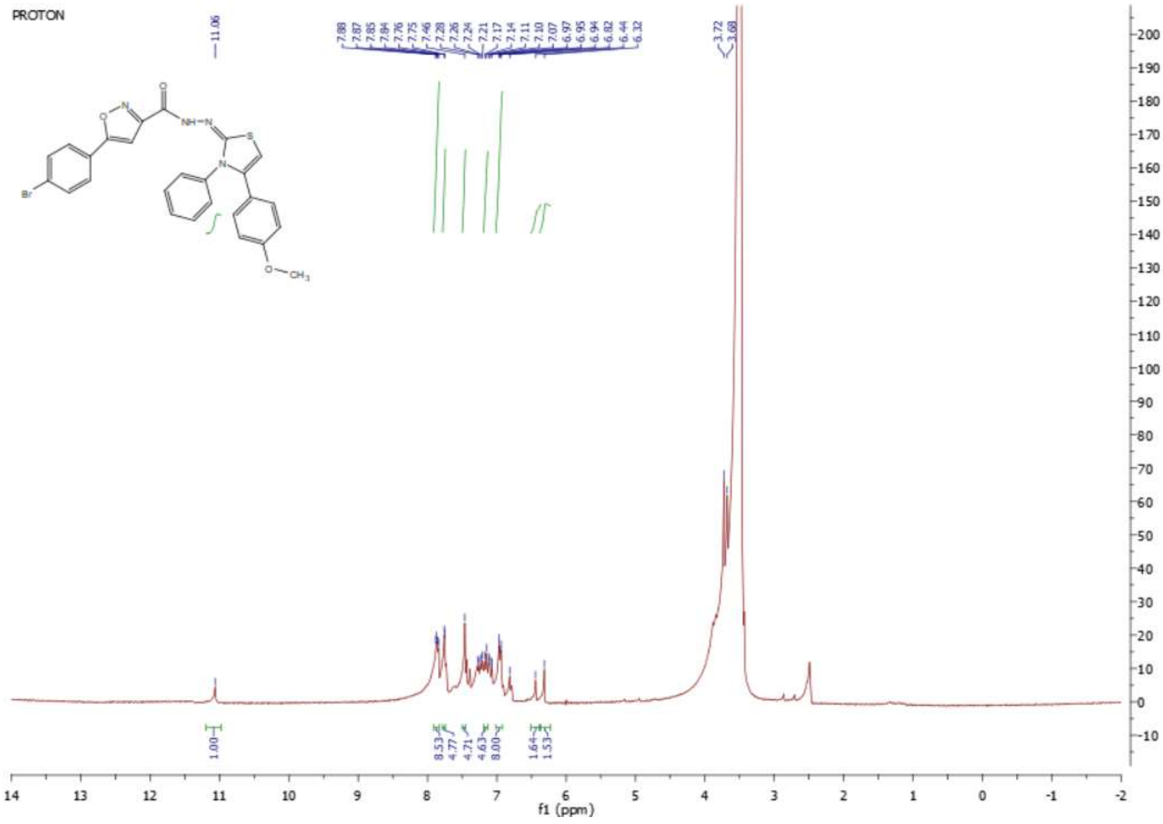

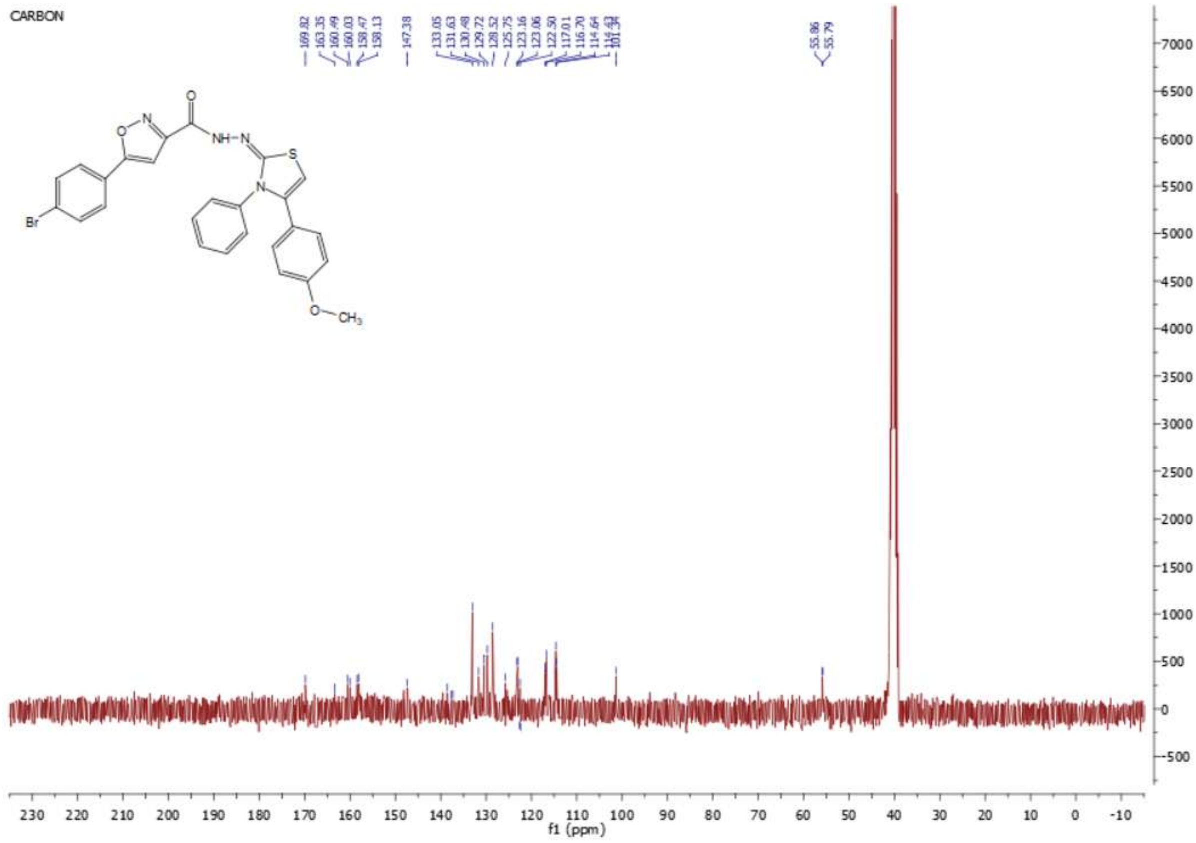

11c) 5-(4-bromofenil)-N'-(4-(4-florofenil)-3-feniltiazol-2(3H)-iliden)izoksazol-3-karbohidrazit:

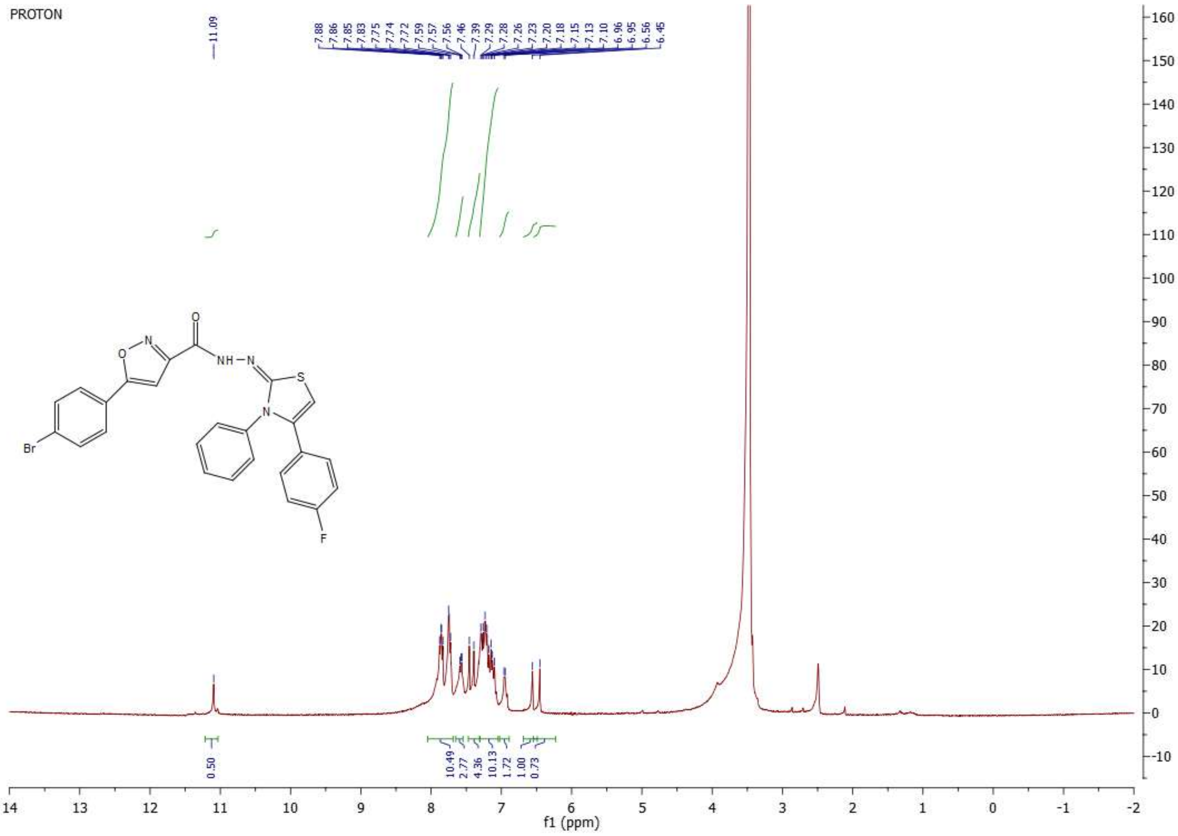

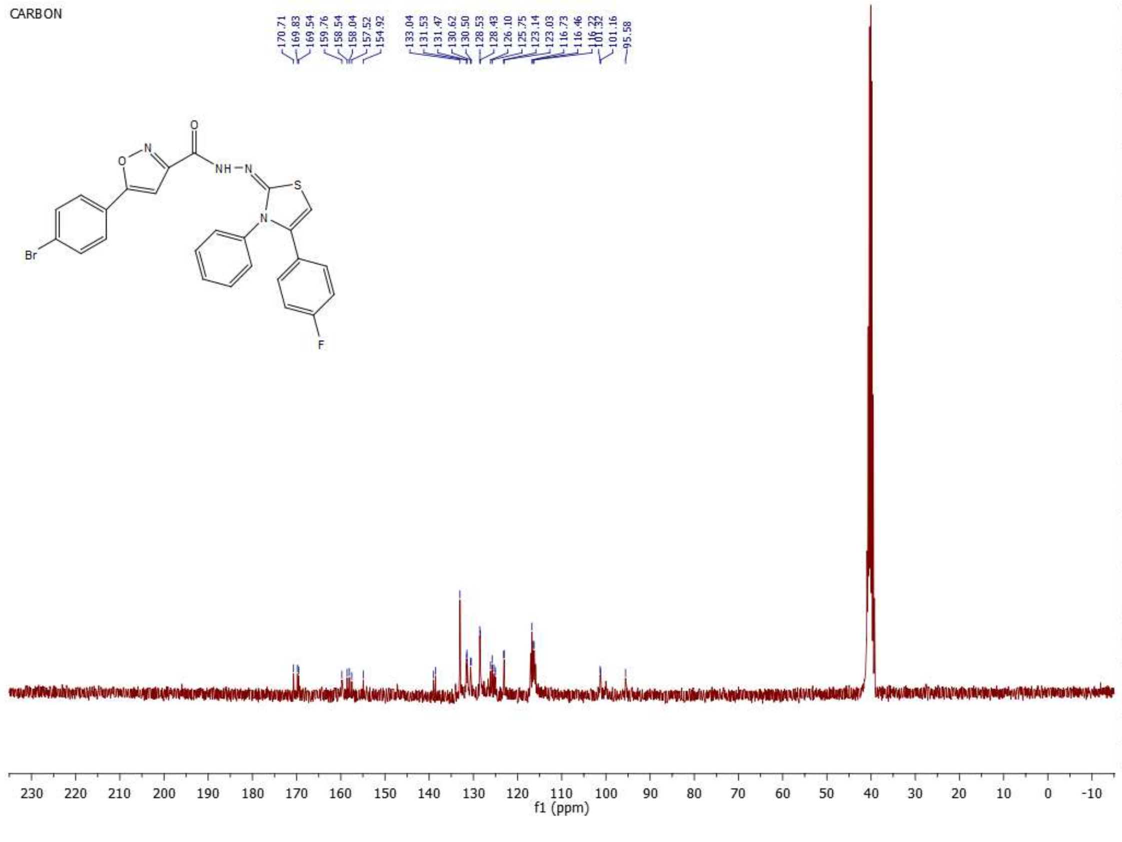

11d) 5-(4-bromofenil)-N'-(3,4-di-p-toliltiazol-2(3H)-iliden)izoksazol-3-karbohidrazid:

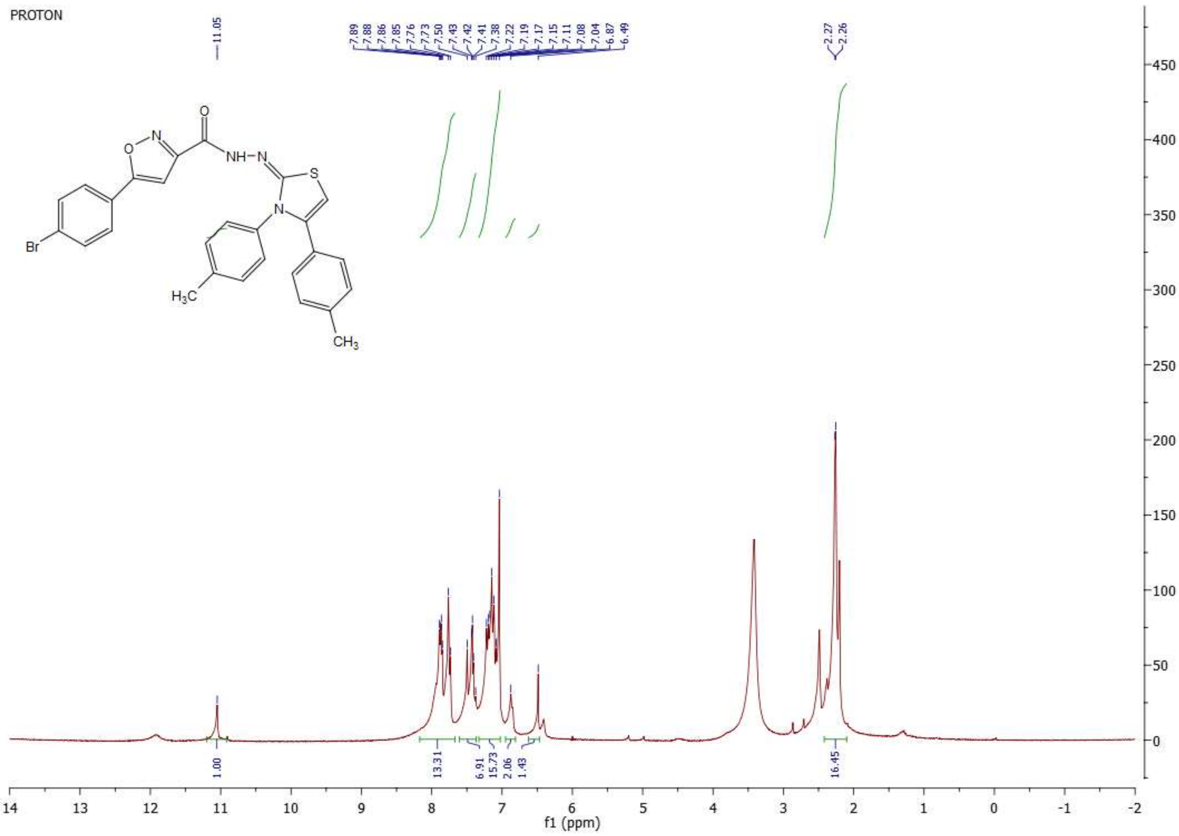

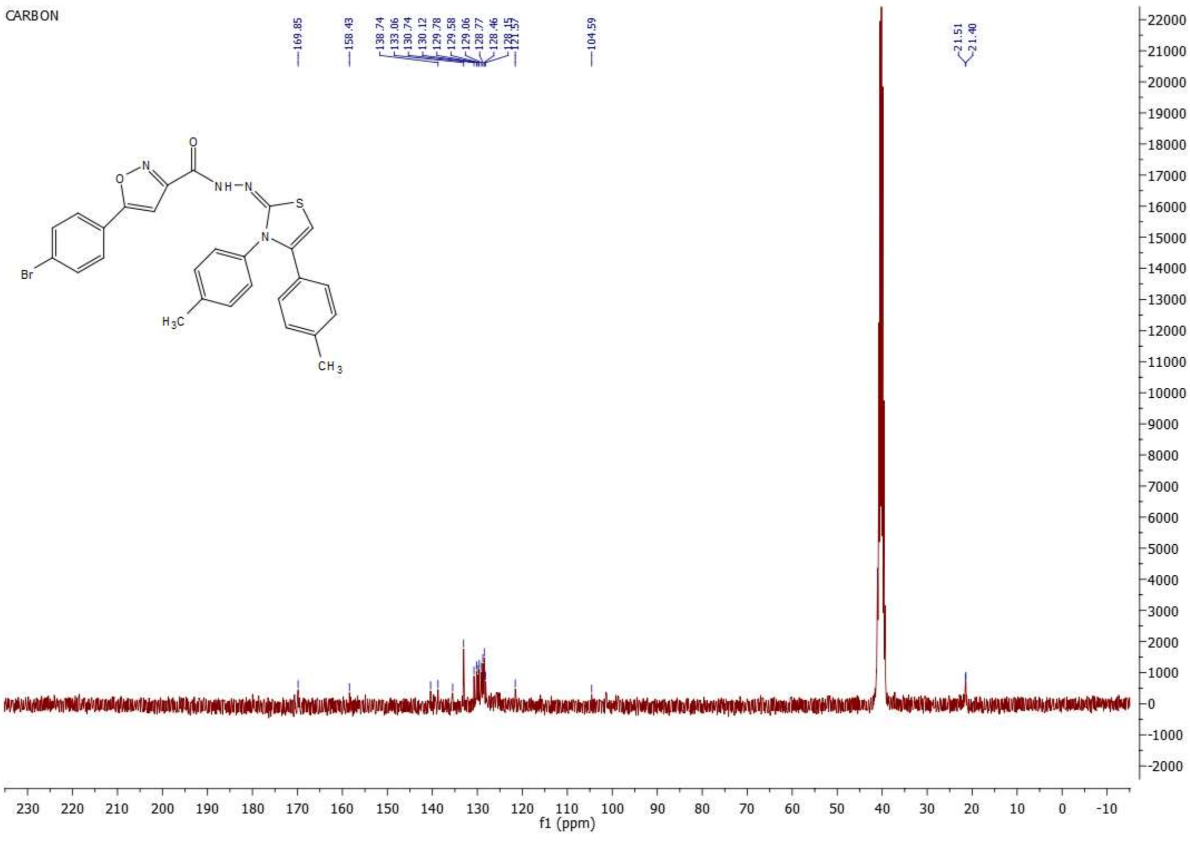

11e) 5-(4-bromofenil)-N'-(4-(4-metoksifenil)-3-(p-tolil)tiazol-2(3H)-iliden)izoksazol-3-karbohidrazit:

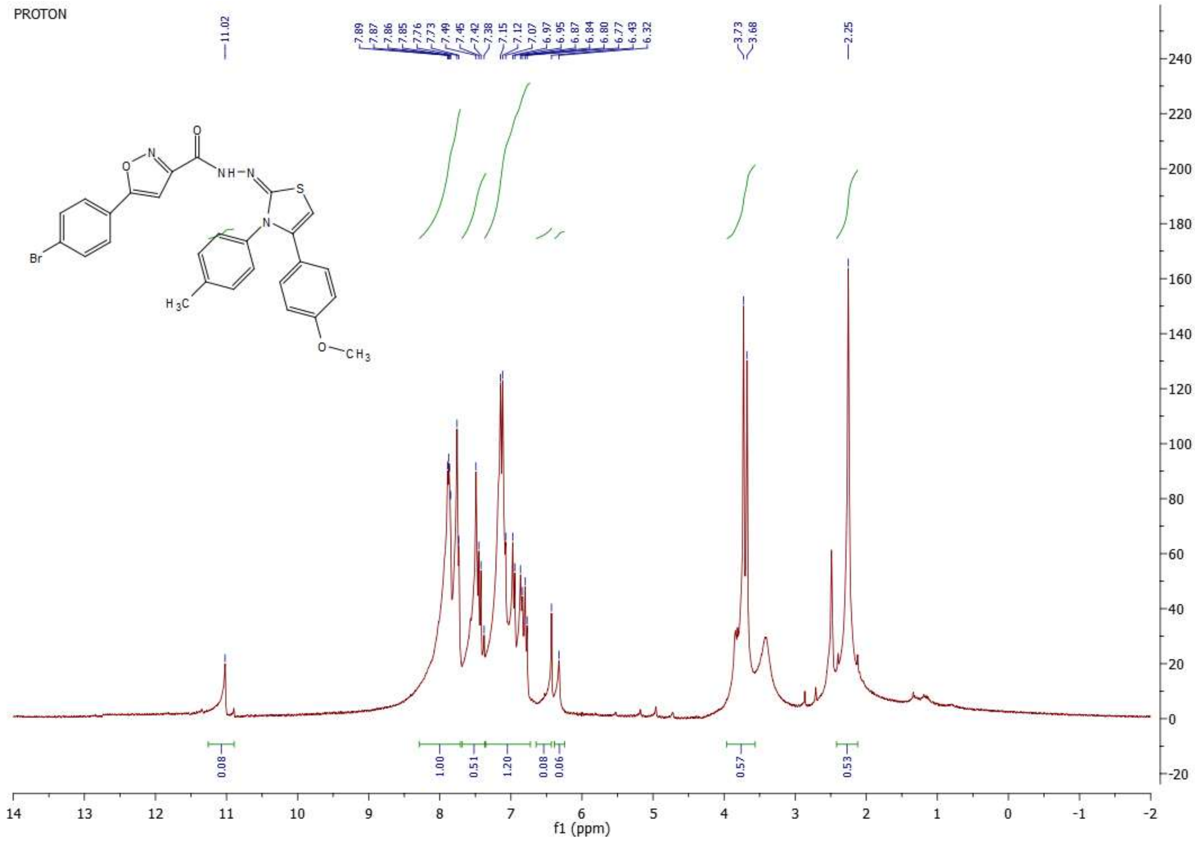

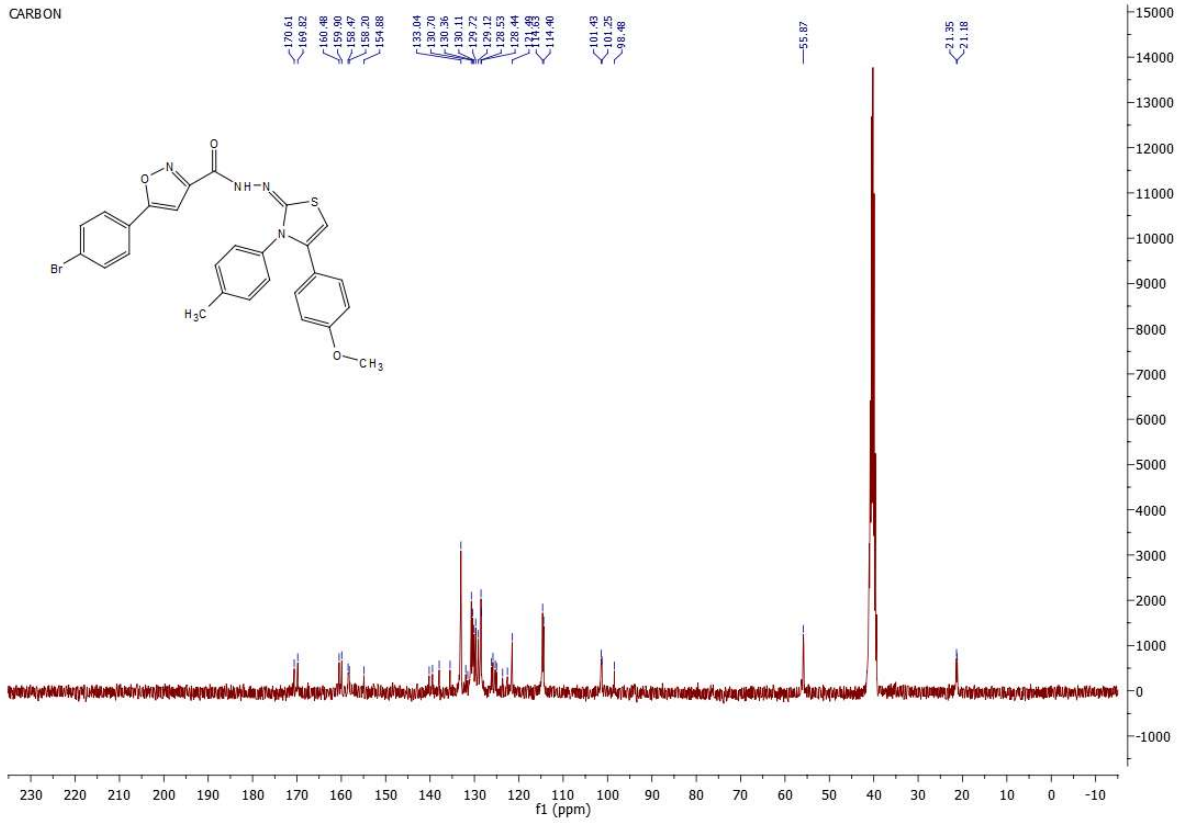

11f) 5-(4-bromofenil)-N'-(4-(4-florofenil)-3-(p-tolil)tiazol-2(3H)-iliden)izoksazol-3-karbohidrazit:

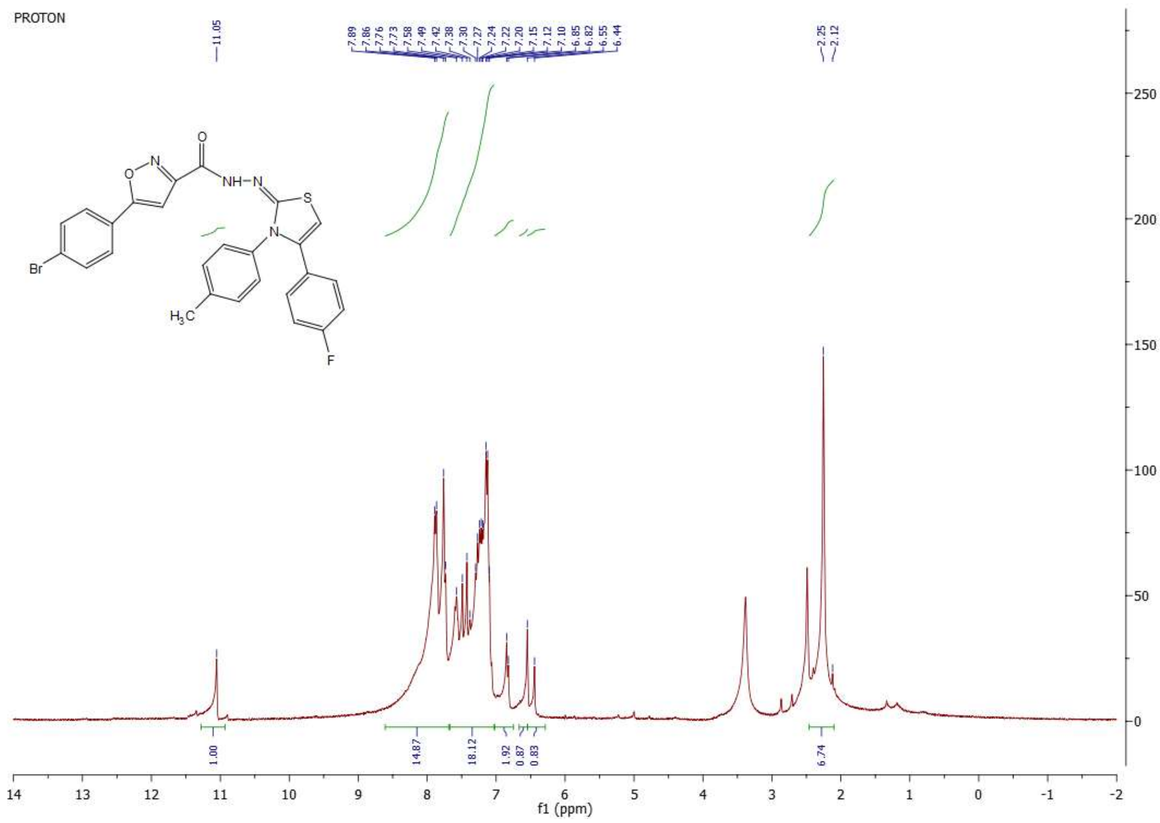

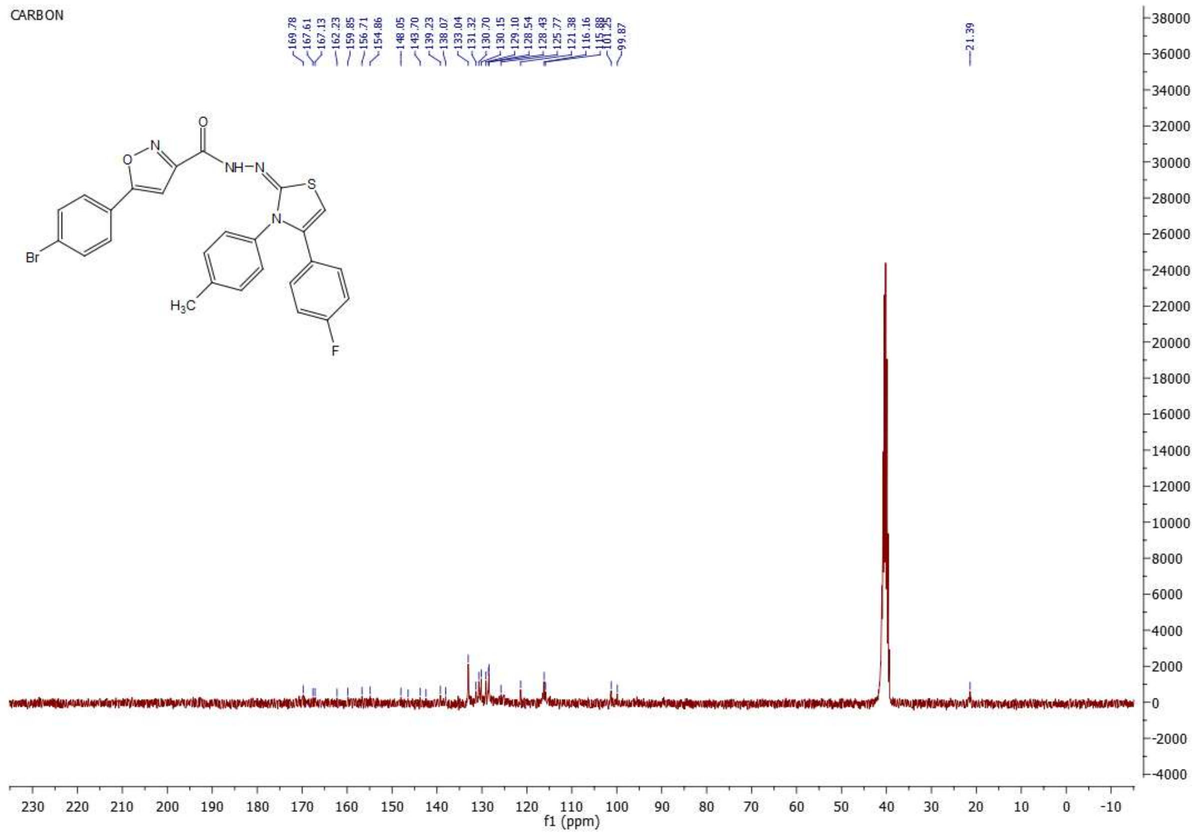

11g) 5-(4-bromofenil)-N'-(3-(4-metoksifenil)-4-(p-tolil)tiazol-2(3H)-iliden)izoksazol-3-karbohidrazit:

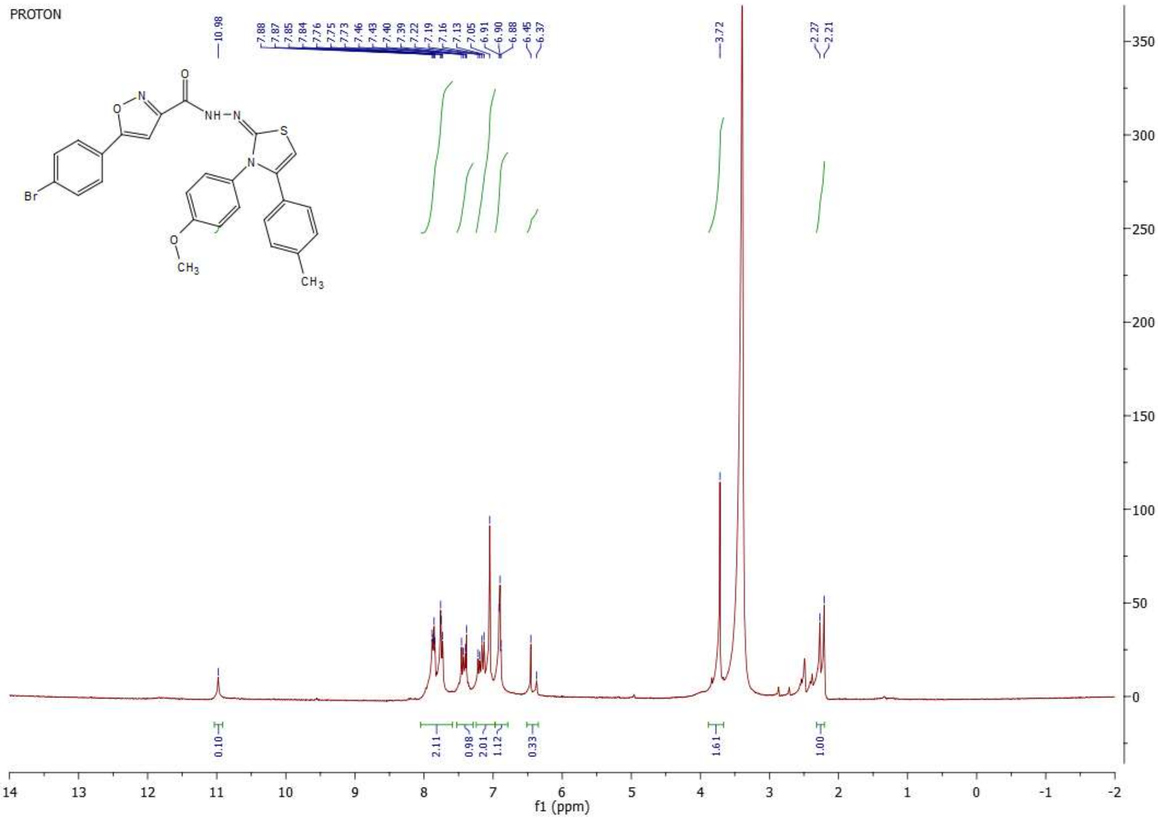

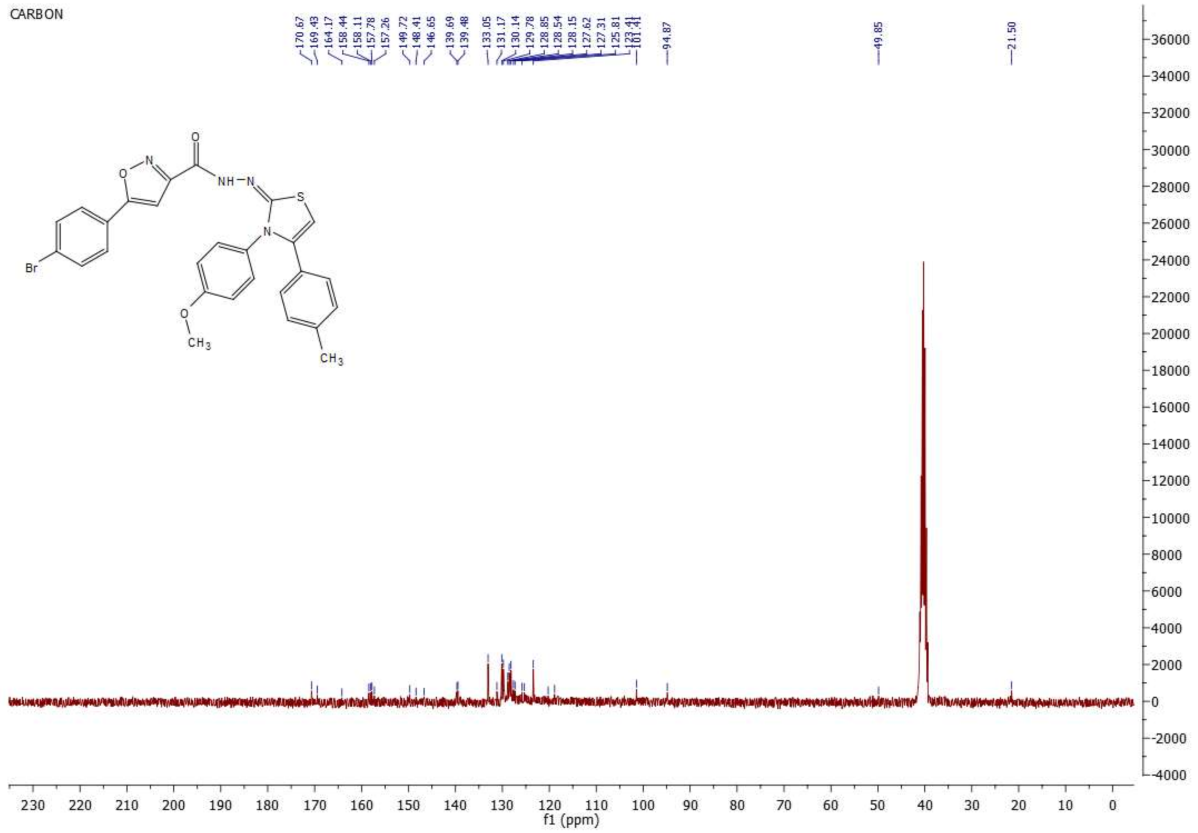

11h) N'-(3,4-bis(4-metoksifenil)tiyazol-2(3H)-iliden)-5-(4-bromofenil)izoksazol-3-karbohidrazit:

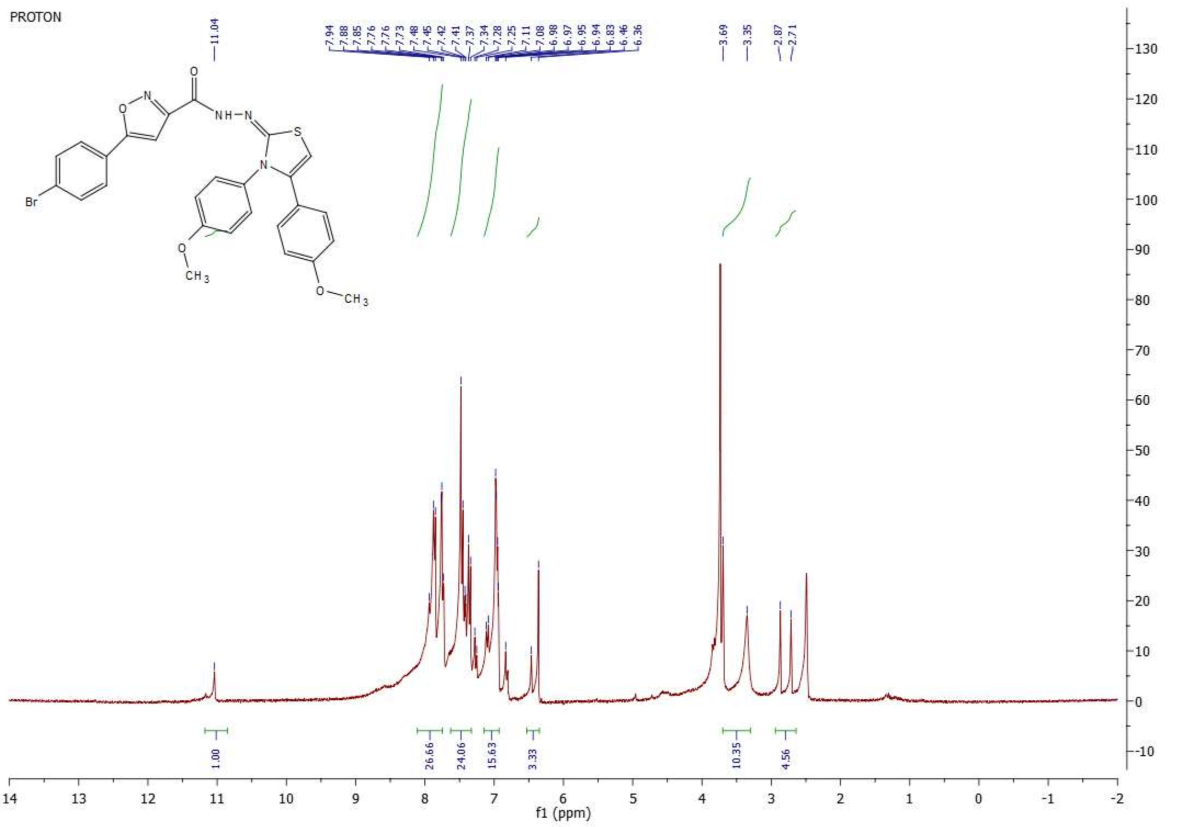

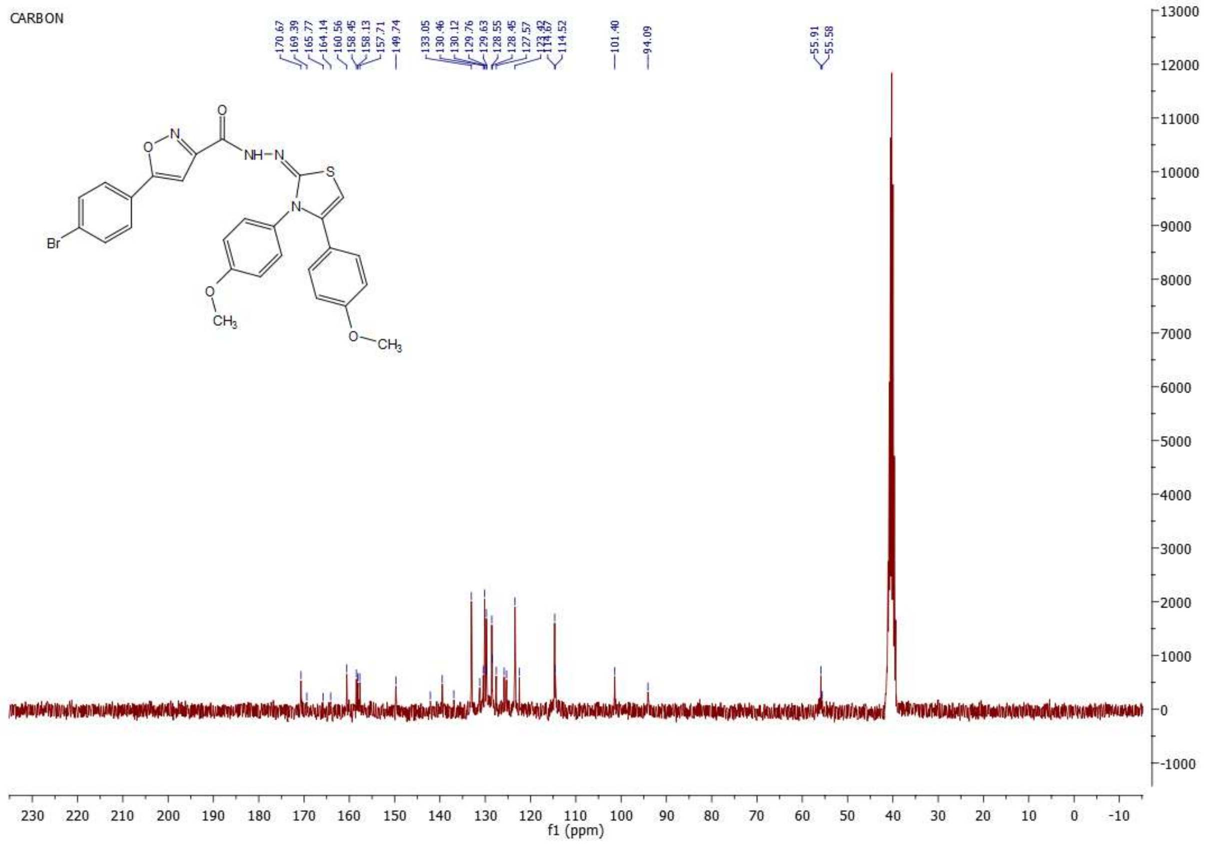

11ı) 5-(4-bromofenil)-N'-(4-(4-florofenil)-3-(4-metoksifenil)tiazol-2(3H)-iliden)izoksazol-3-karbohidrazit:

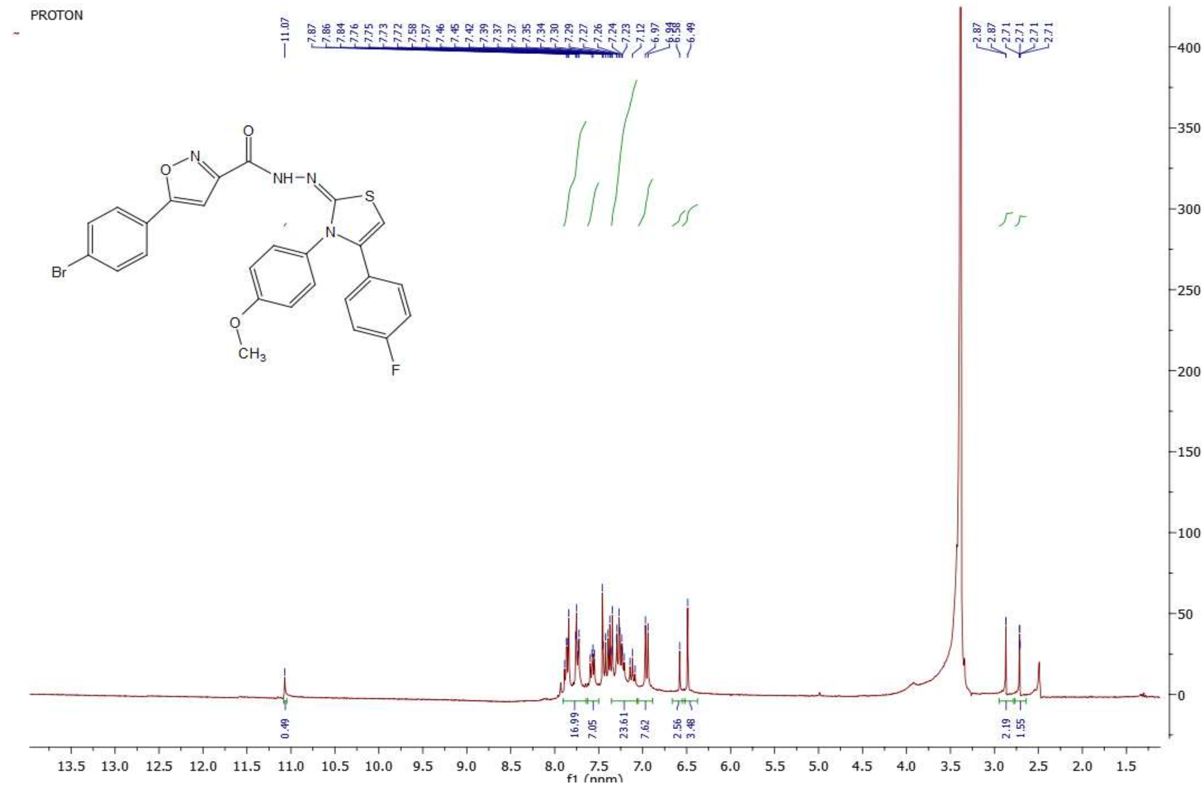

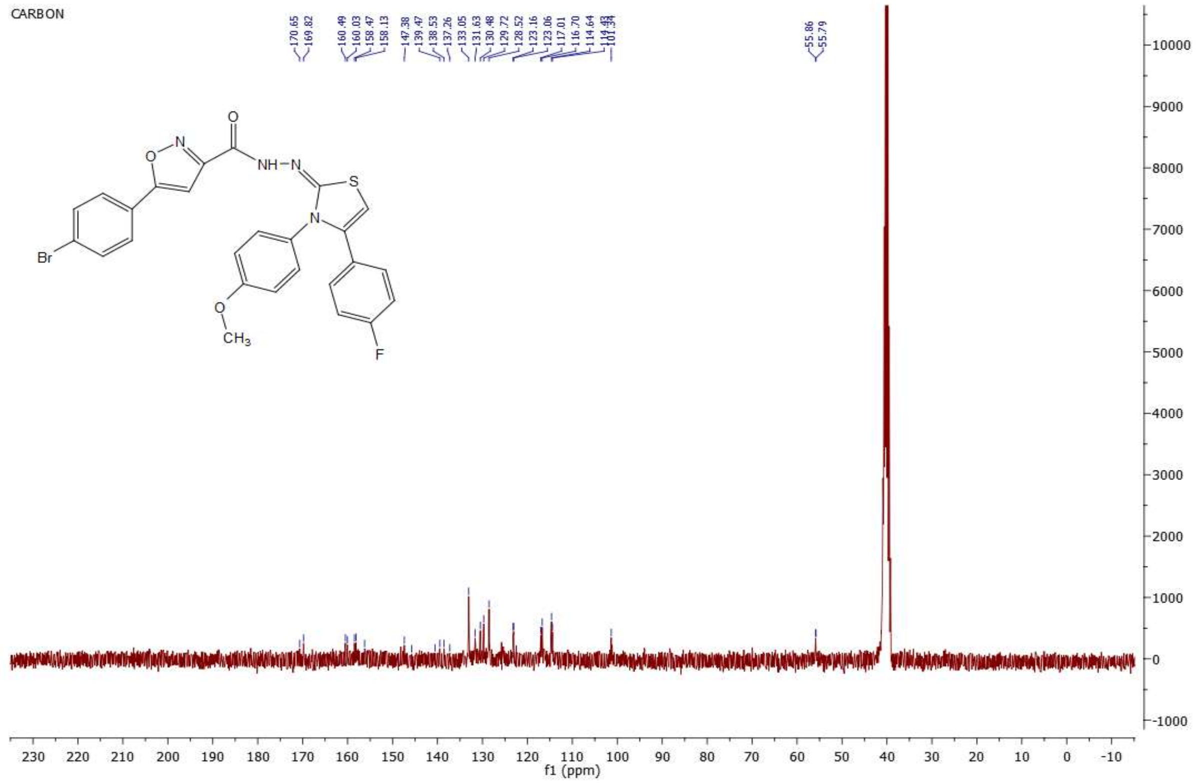

11i) 5-(4-bromophenyl)-N'-(3-(4-fluorophenyl)-4-(p-tolyl)thiazol-2(3H)-ylidene)isoxazole-3-carbohydrazide:

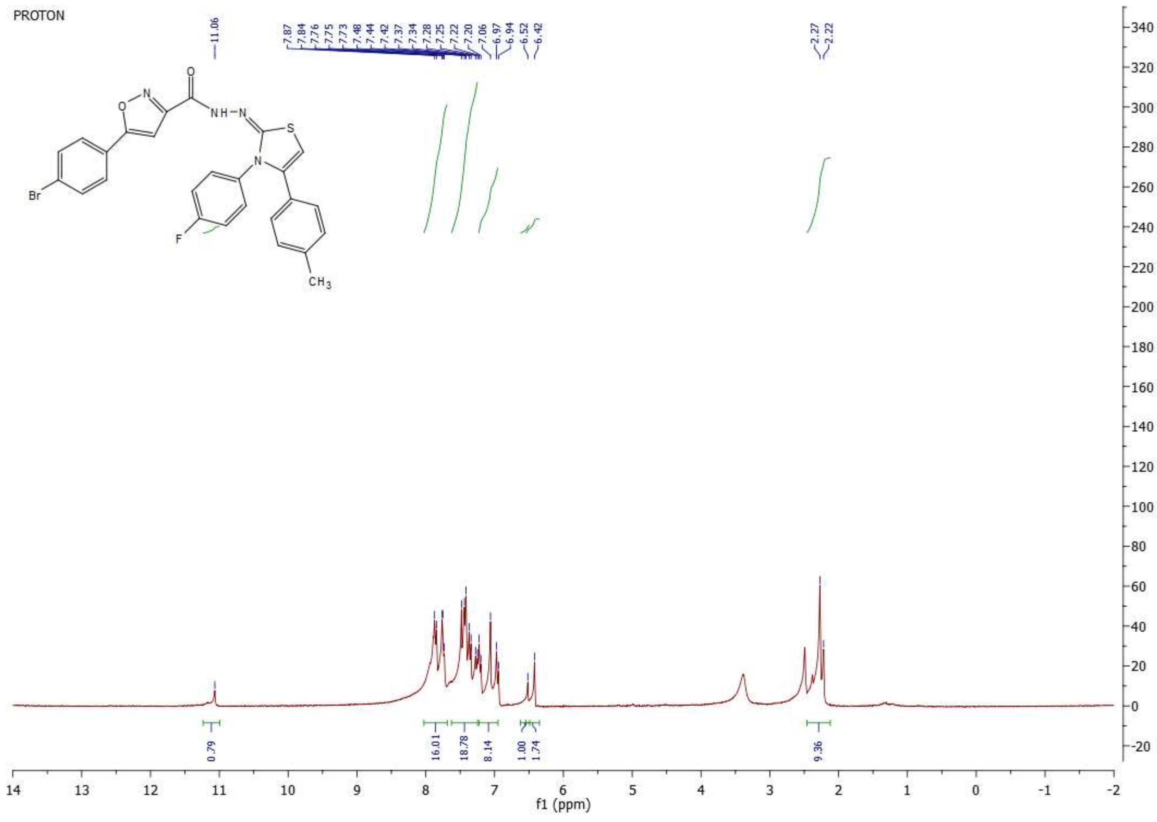

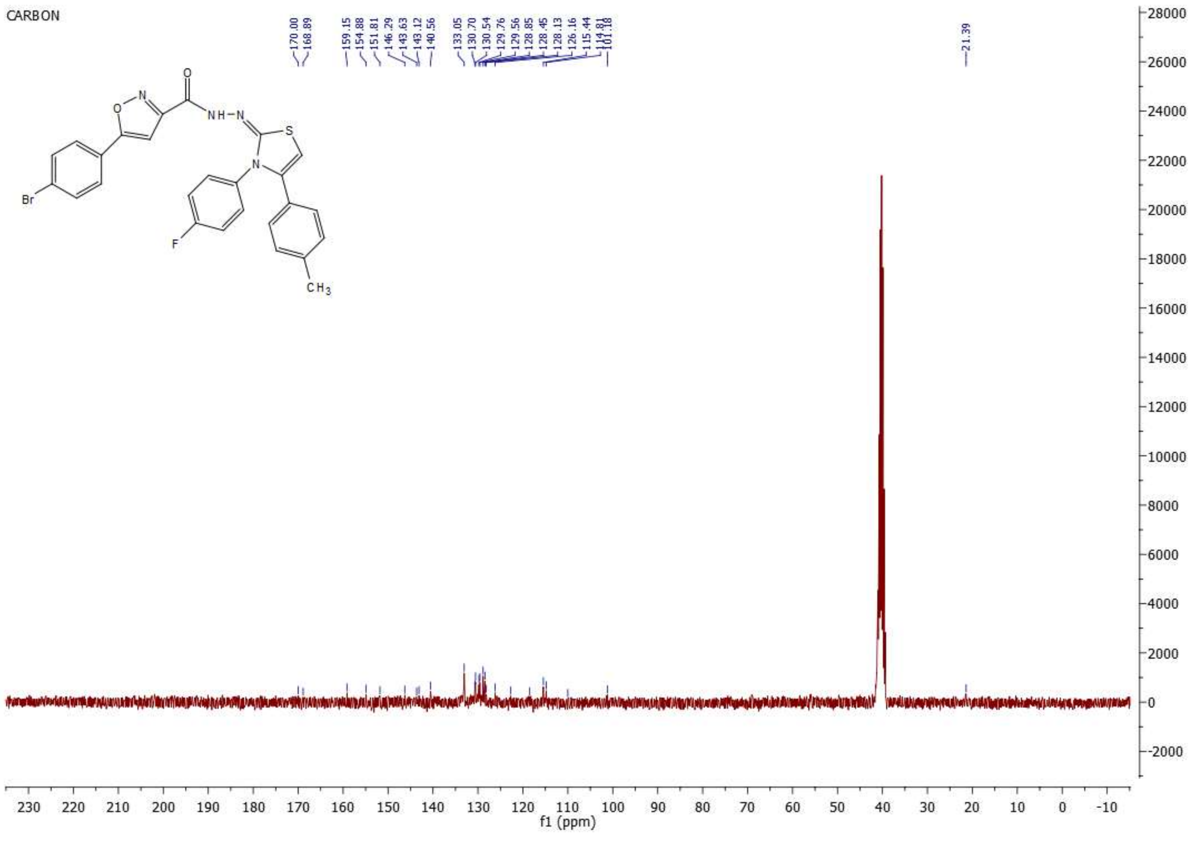

11j)5-(4-bromofenil)-N'-(3-(4-florofenil)-4-(4-metoksifenil)tiazol-2(3H)-iliden)izoksazol-3-karbohidrazit:

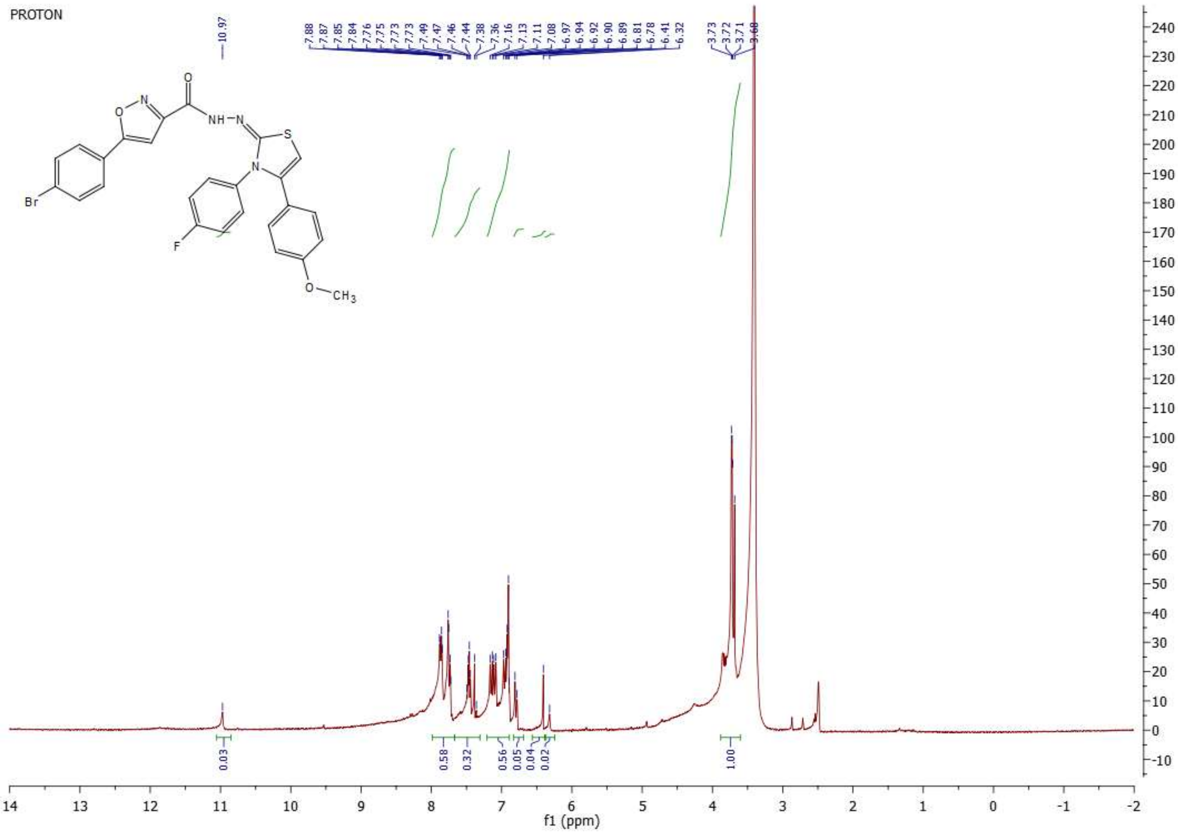

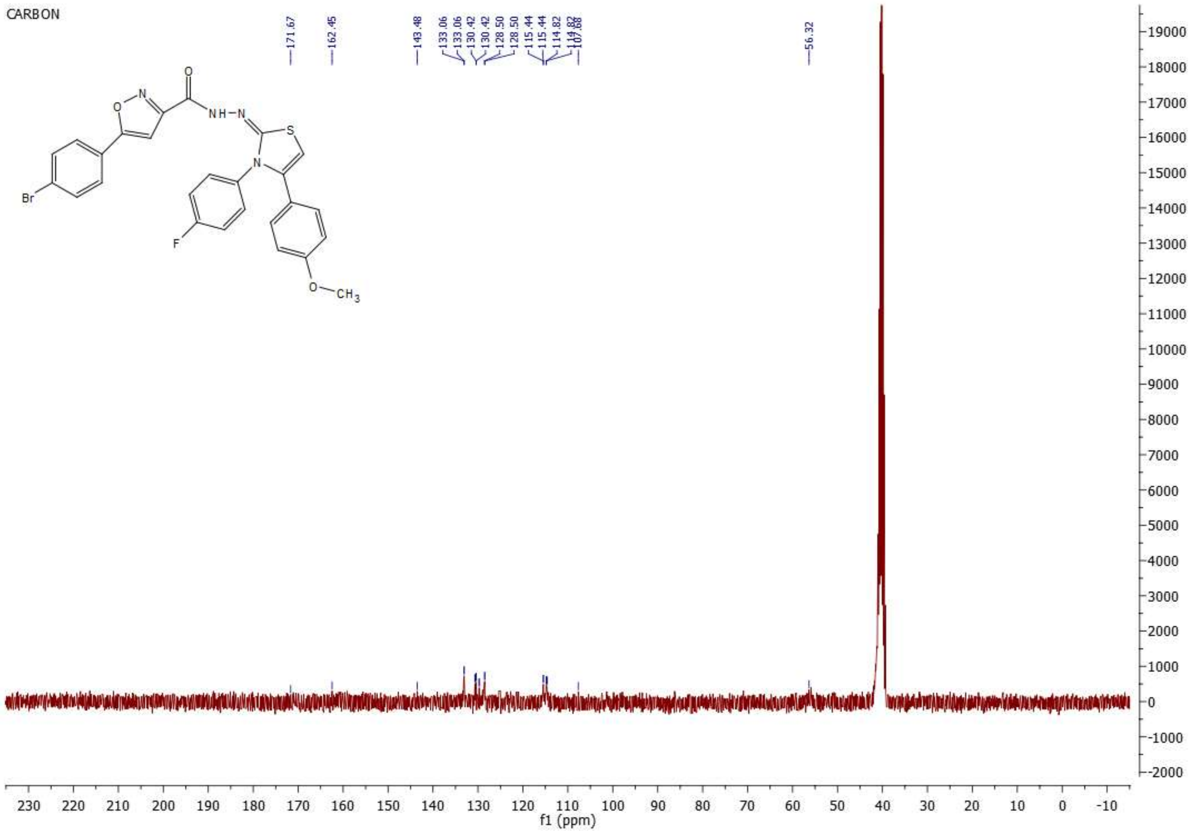

11k) N'-(3,4-bis(4-florofenil)tiazol-2(3H)-iliden)-5-(4-bromofenil)izoksazol-3-karbohidrazit:

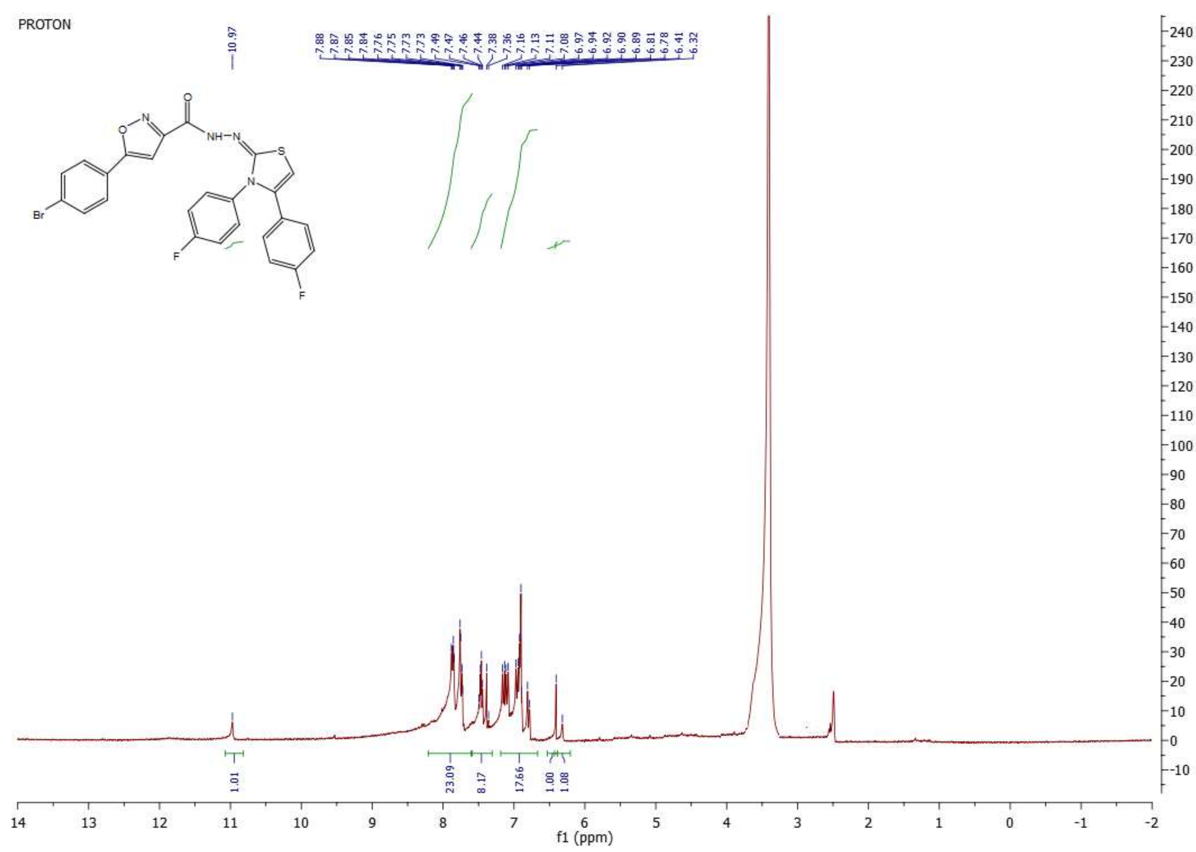

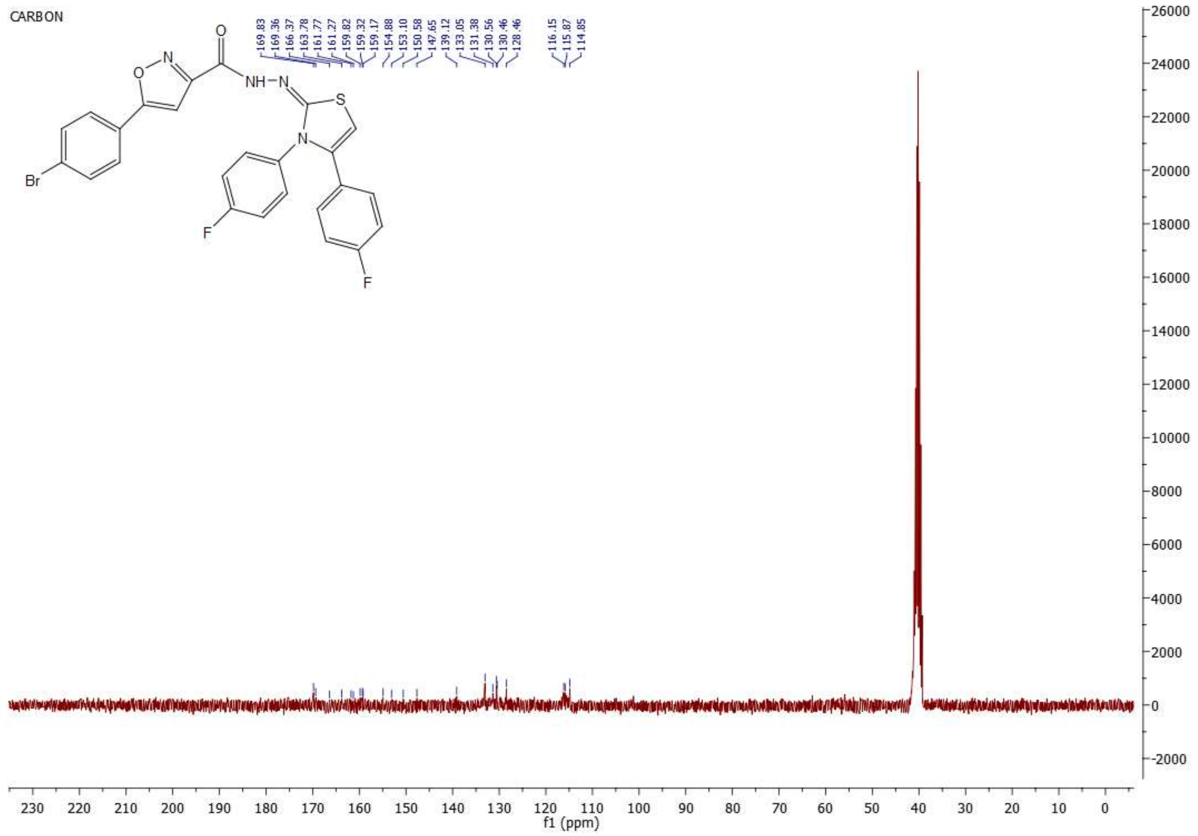

11l) 5-(4-bromofenil)-N'-(3-(4-klorofenil)-4-(p-tolil)tiazol-2(3H)-iliden)izoksazol-3-karbohidrazid:

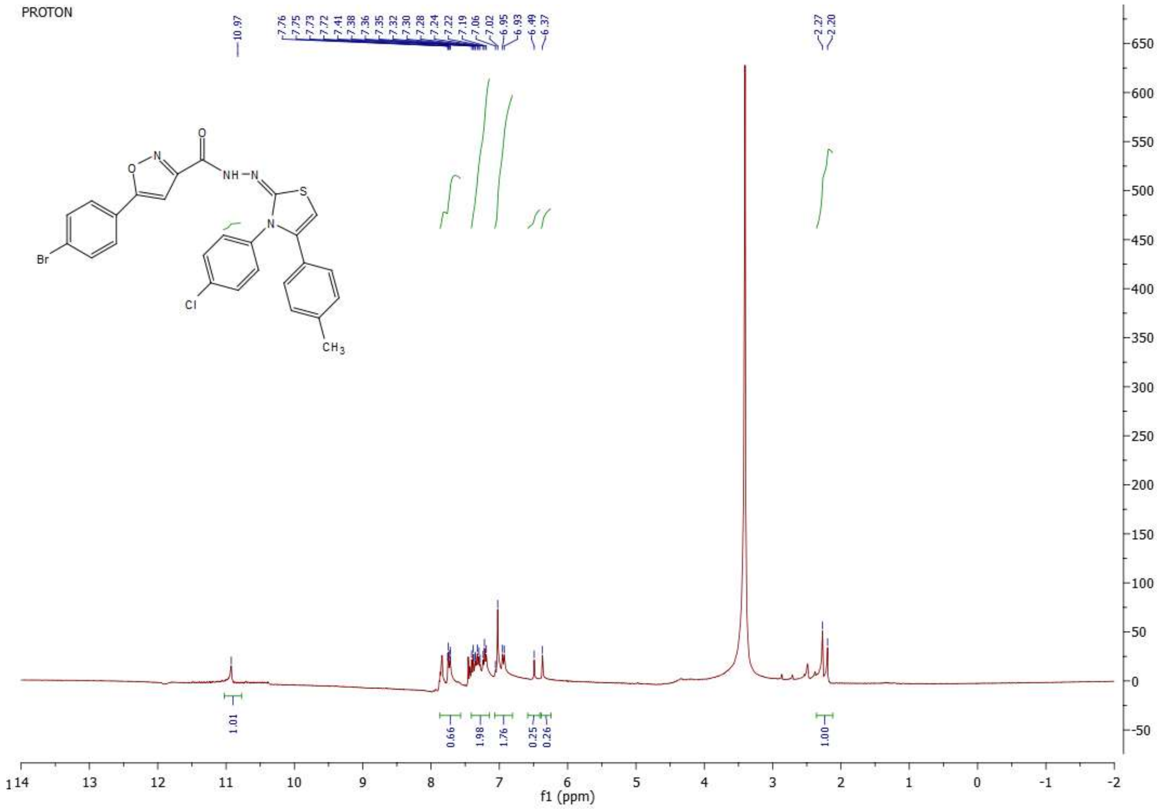

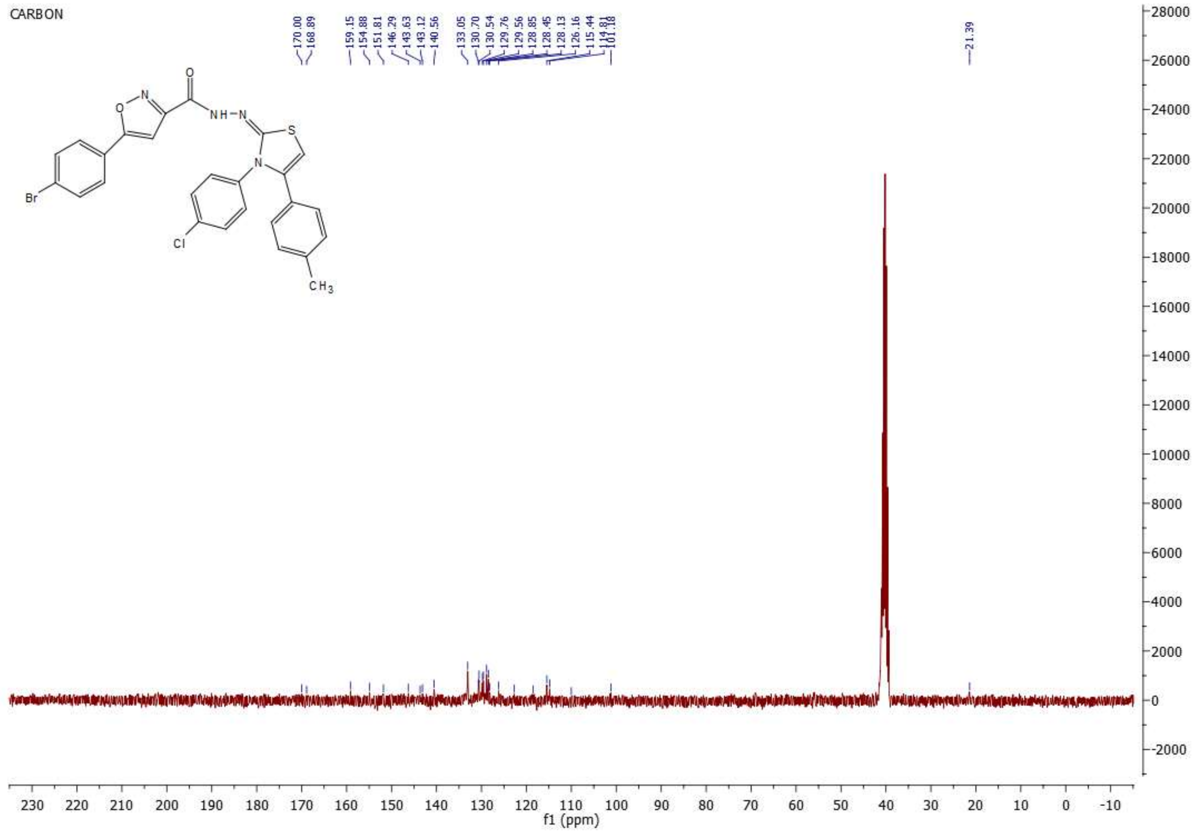

11m)5-(4-bromofenil)-N'-(3-(4-klorofenil)-4-(4-metoksifenil)tiazol-2(3H)-iliden)izoksazol-3-karbohidrazid:

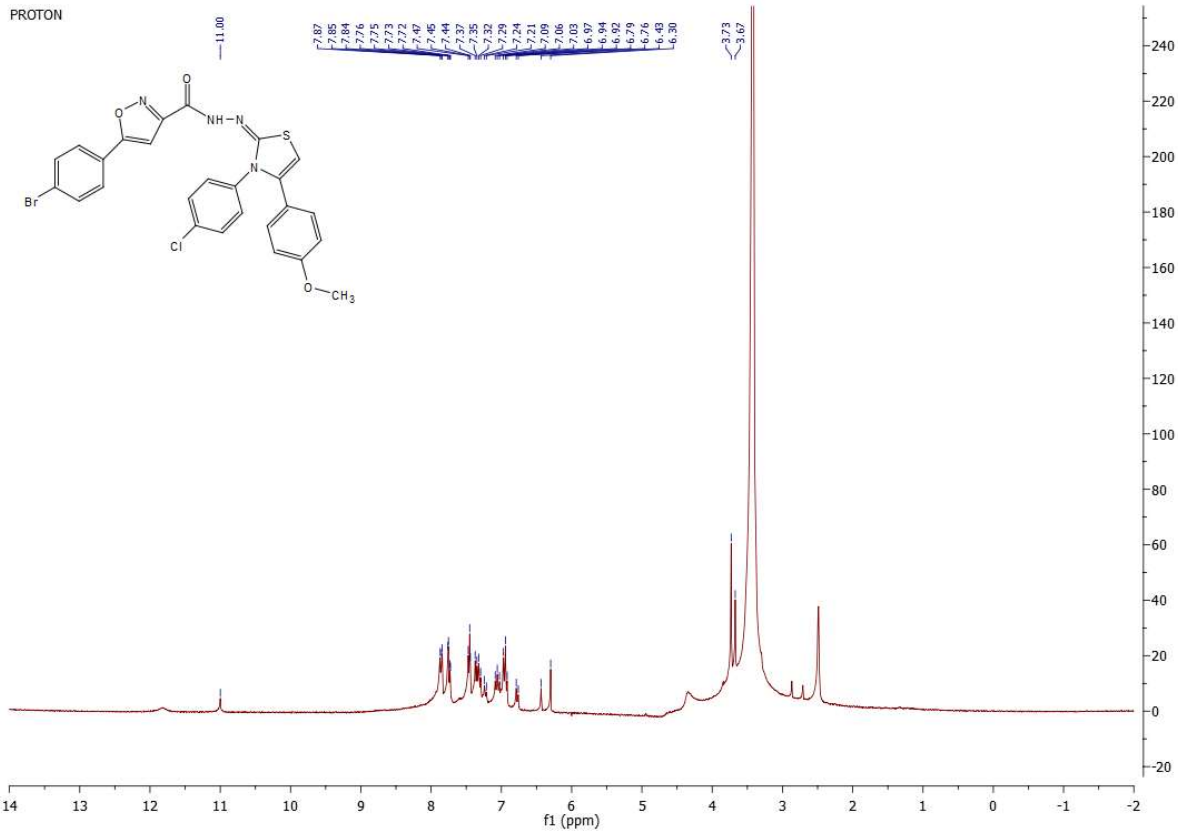

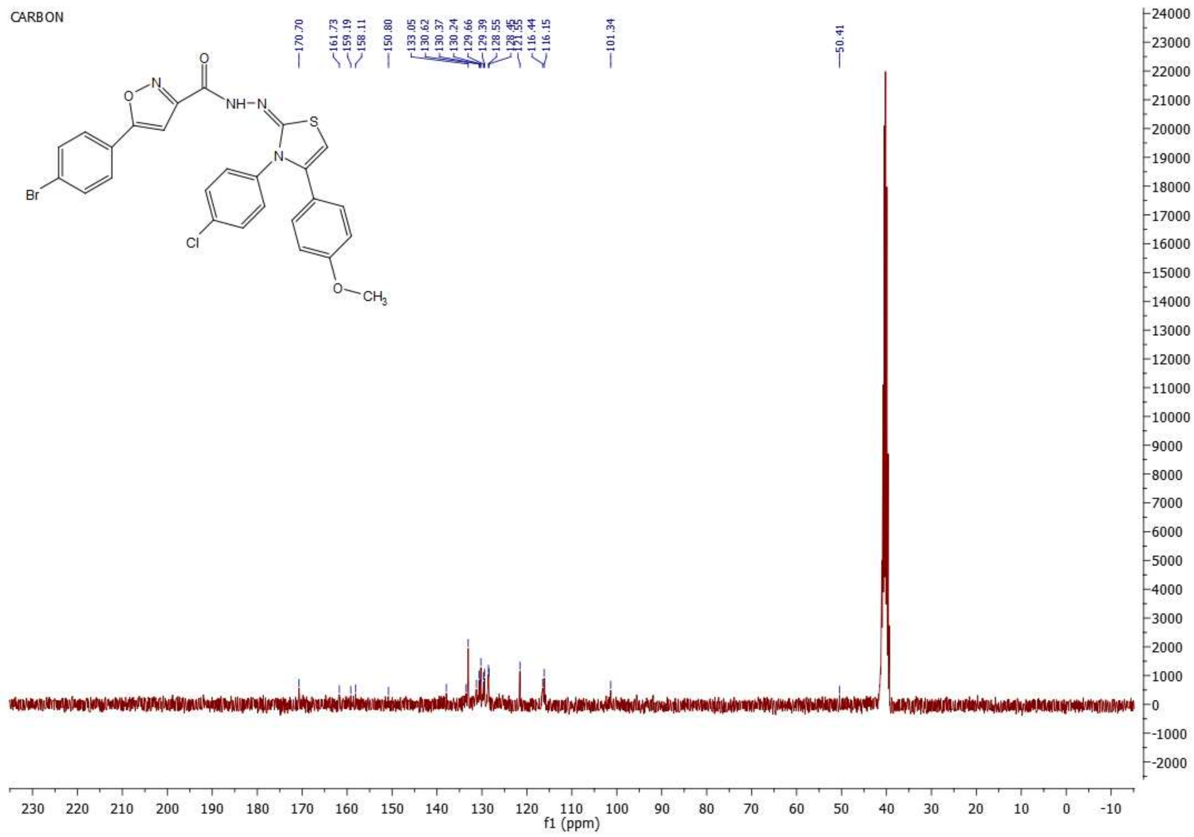

11n)5-(4-bromofenil)-N'-(3-(4-klorofenil)-4-(4-florofenil)tiazol-2(3H)-iliden)izoksazol-3-karbohidrazid:

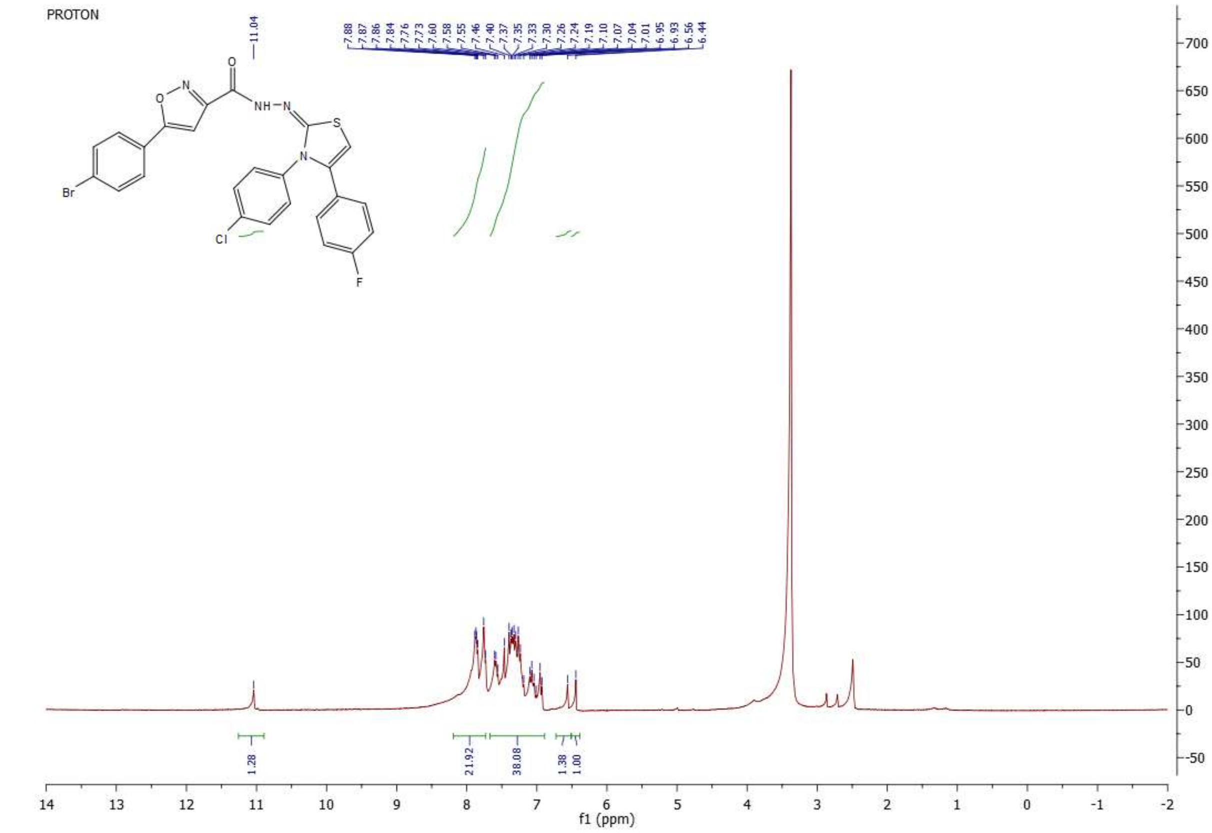

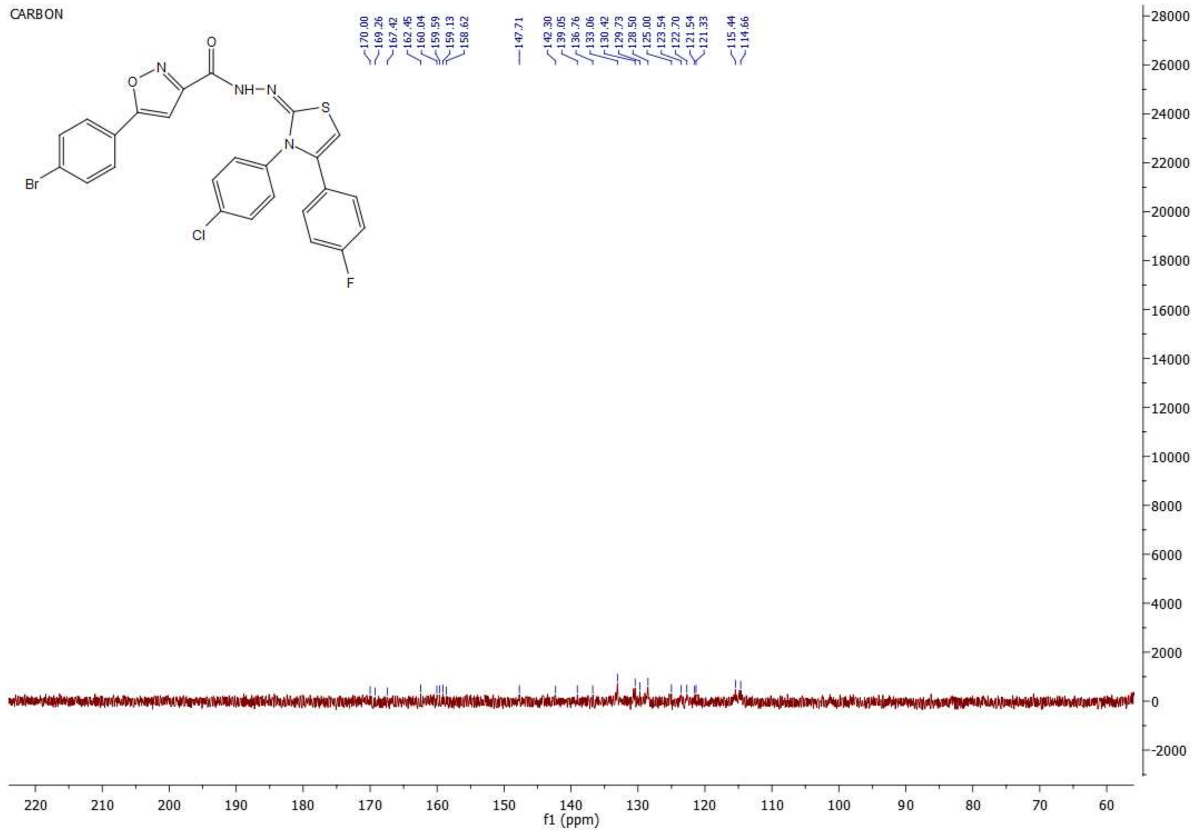

Scheme 2: IR spectra

5-(4-bromophenyl)-N'-(3-phenyl-4-p-tolylthiazol-2(3H)-ylidene)isoxazole-3-carbohydrazide (11a):

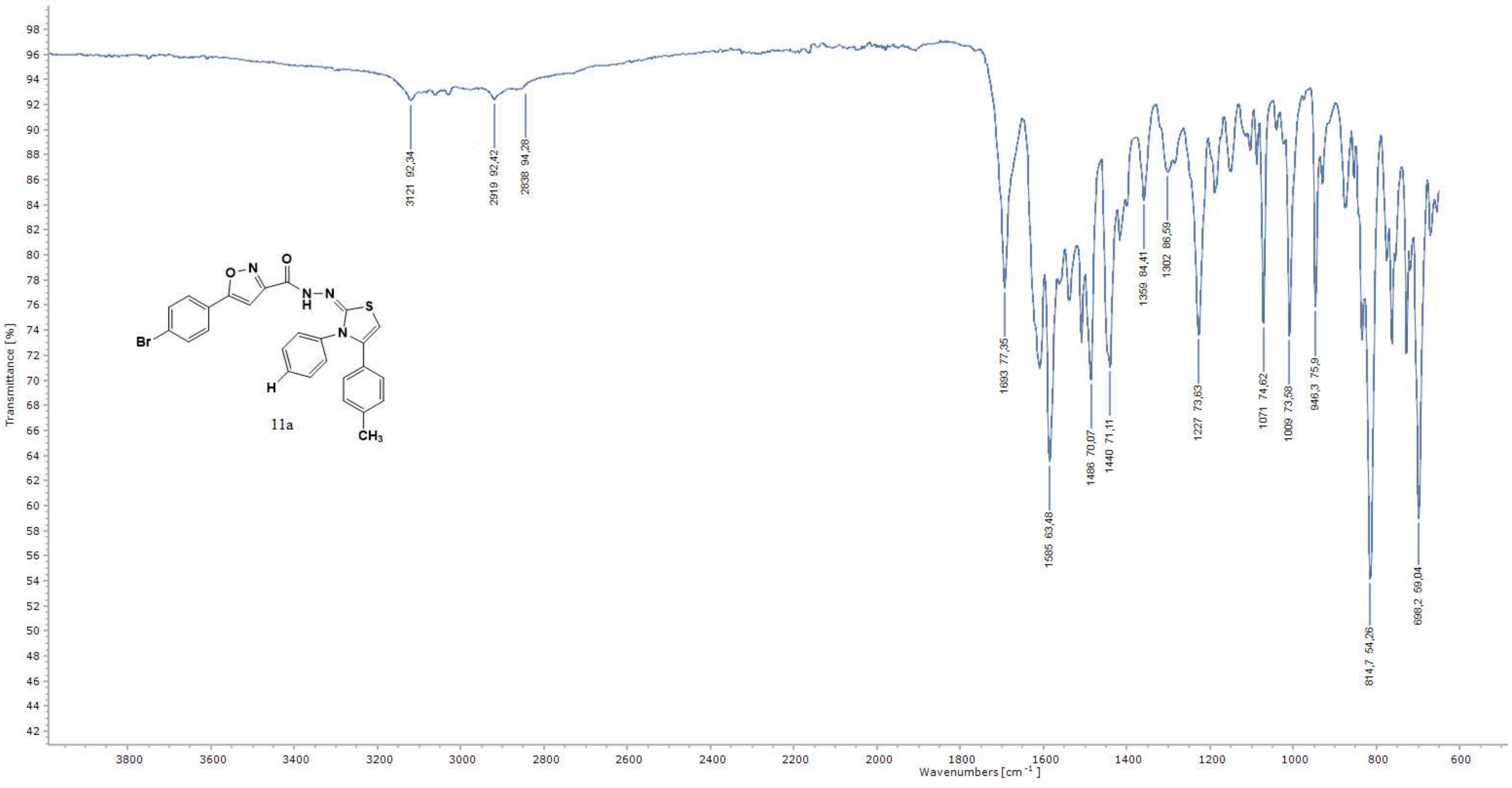

5-(4-bromophenyl)-N'-(4-(4-methoxyphenyl)-3-phenylthiazol-2(3H)-ylidene)isoxazole-3-carbohydrazide (11b):

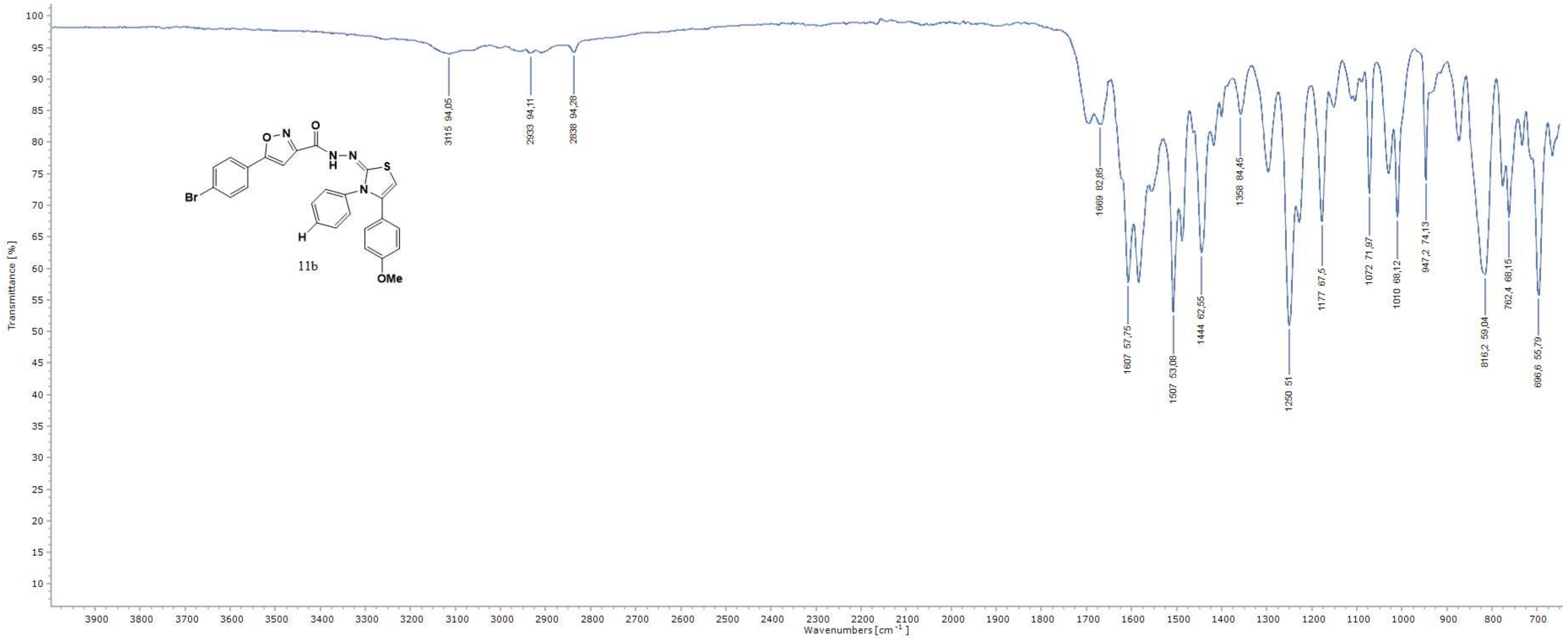

5-(4-bromophenyl)-N'-(4-(4-fluorophenyl)-3-phenylthiazol-2(3H)-ylidene)isoxazole-3-carbohydrazide (11c):

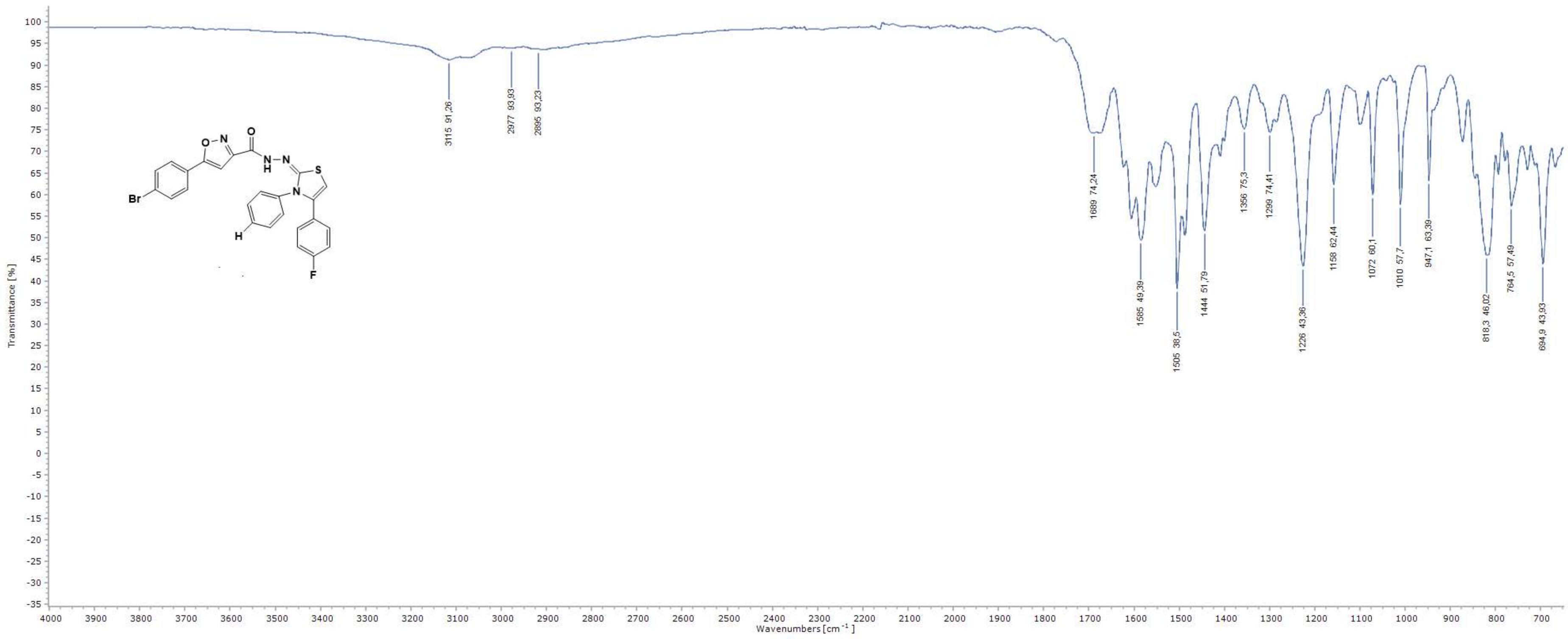

5-(4-bromophenyl)-N'-(3,4-dip-tolylthiazol-2(3H)-ylidene)isoxazole-3-carbohydrazide (11d):

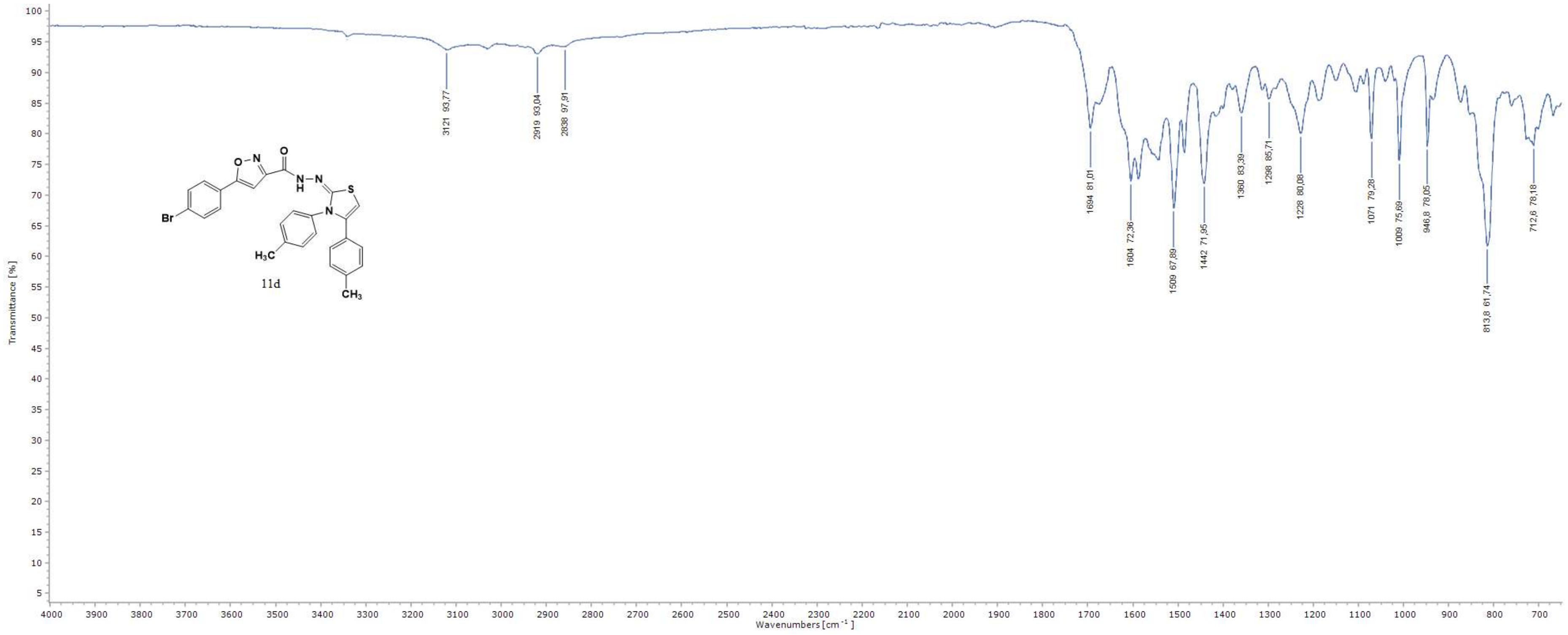

5-(4-bromophenyl)-N'-(4-(4-methoxyphenyl)-3-p-tolylthiazol-2(3H)-ylidene)isoxazole-3-carbohydrazide (11e):

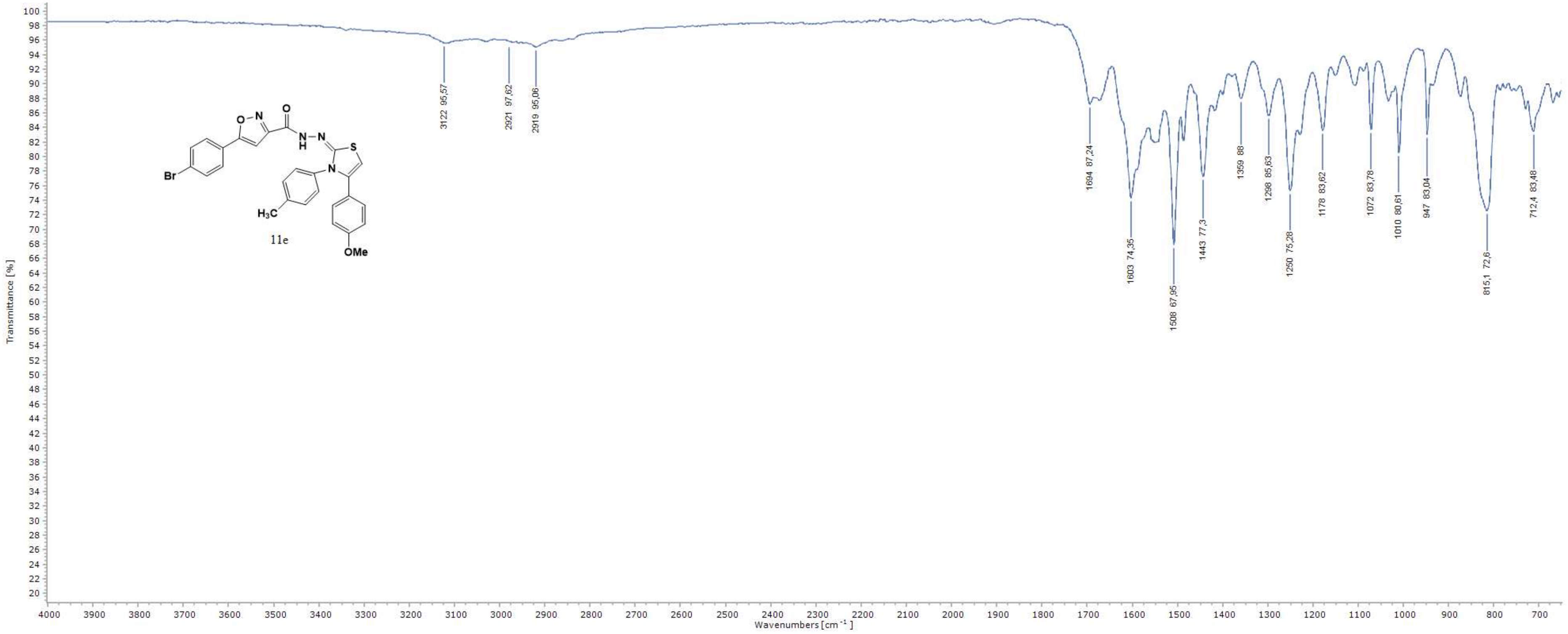

5-(4-bromophenyl)-N'-(4-(4-fluorophenyl)-3-p-tolylthiazol-2(3H)-ylidene)isoxazole-3-carbohydrazide (11f):

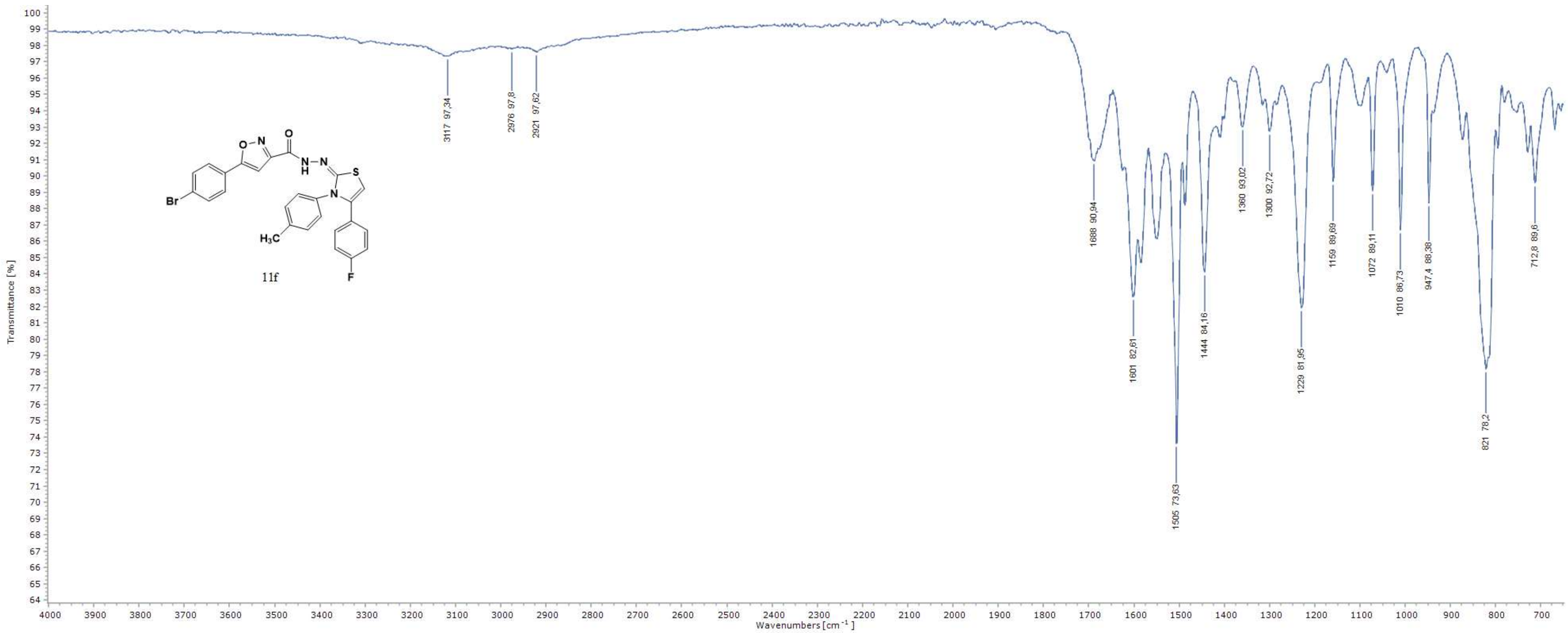

5-(4-bromophenyl)-N'-(3-(4-methoxyphenyl)-4-p-tolylthiazol-2(3H)-ylidene)isoxazole-3-carbohydrazide (11g):

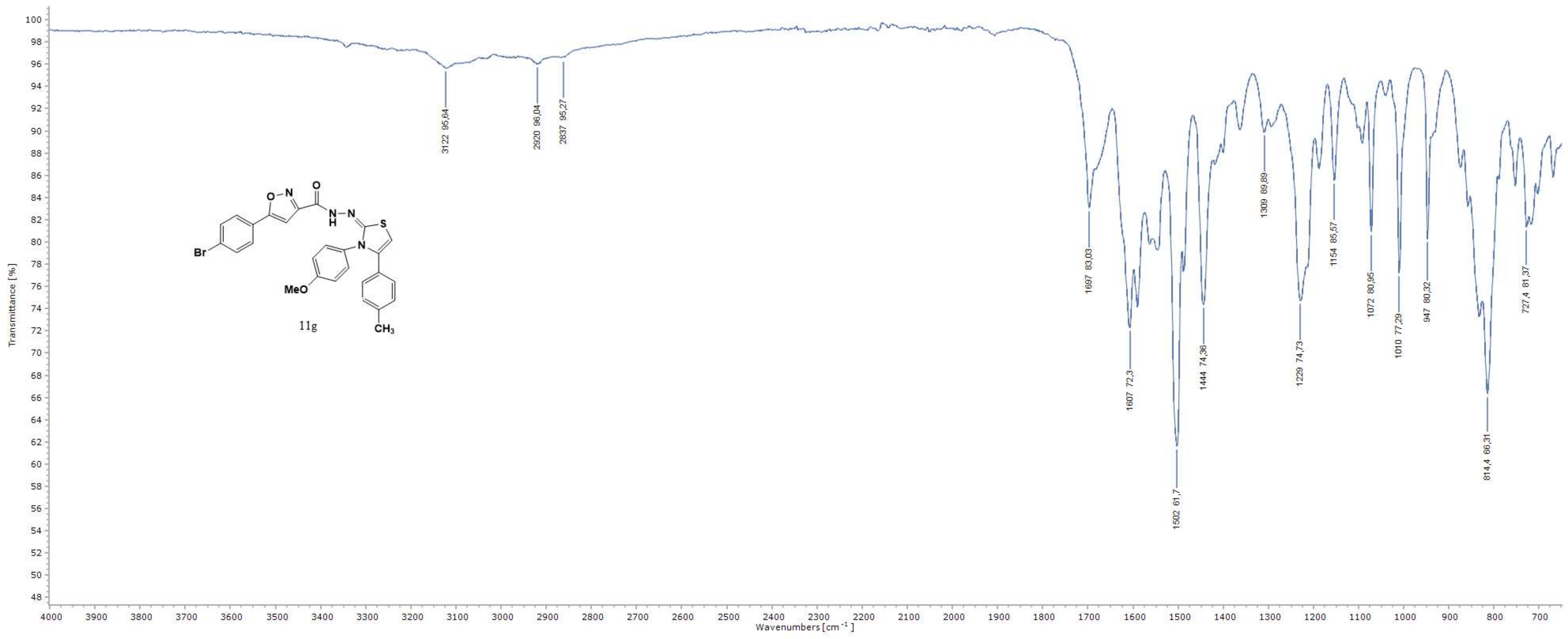

N'-(3,4-bis(4-methoxyphenyl)thiazol-2(3H)-ylidene)-5-(4-bromophenyl)isoxazole-3-carbohydrazide (11h):

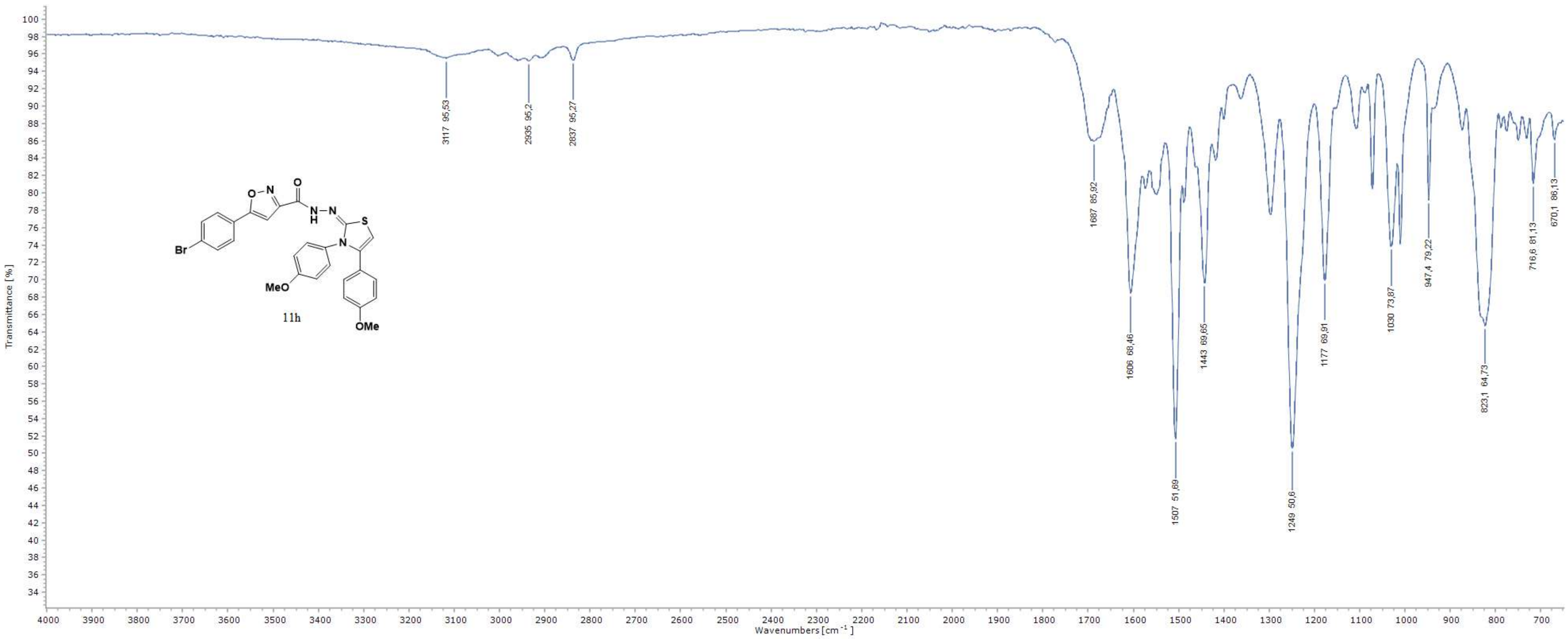

5-(4-bromophenyl)-N'-(4-(4-fluorophenyl)-3-(4-methoxyphenyl)thiazol-2(3H)-ylidene)isoxazole-3-carbohydrazide (11ı):

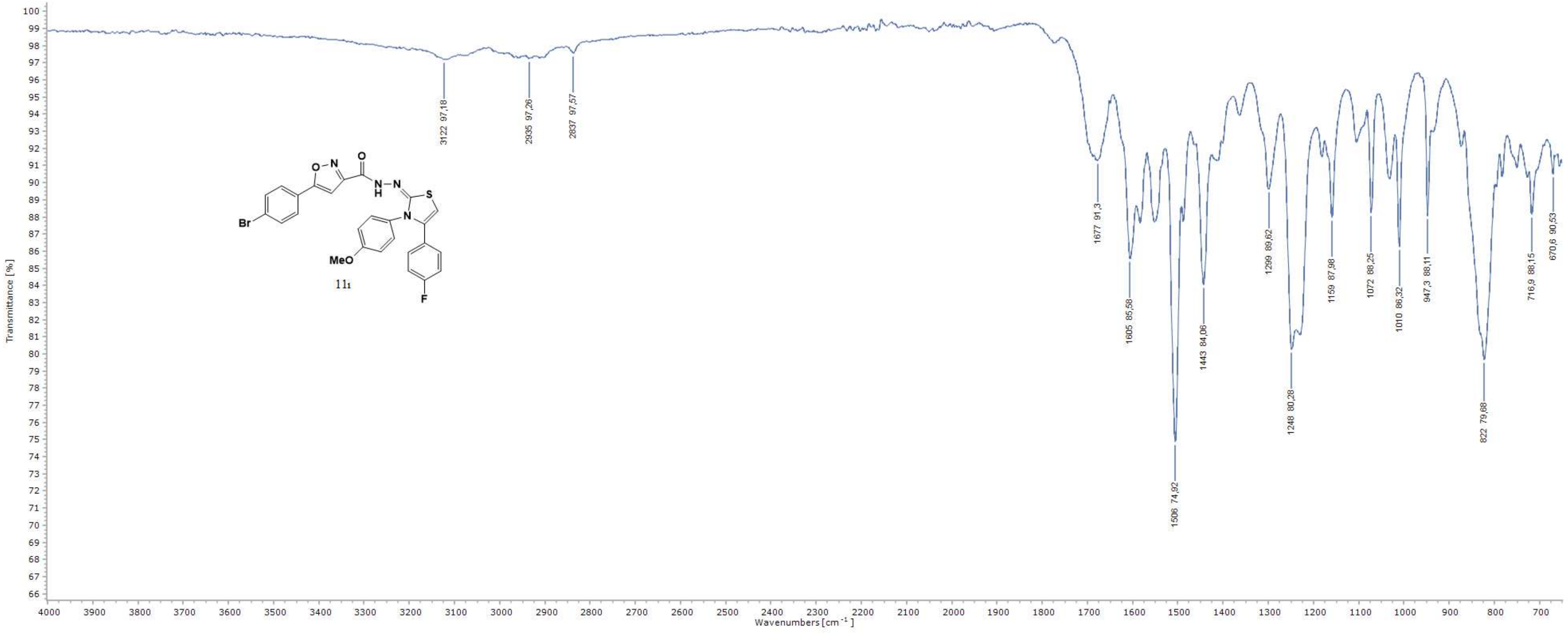

5-(4-bromophenyl)-N'-(3-(4-fluorophenyl)-4-p-tolylthiazol-2(3H)-ylidene)isoxazole-3-carbohydrazide (11i):

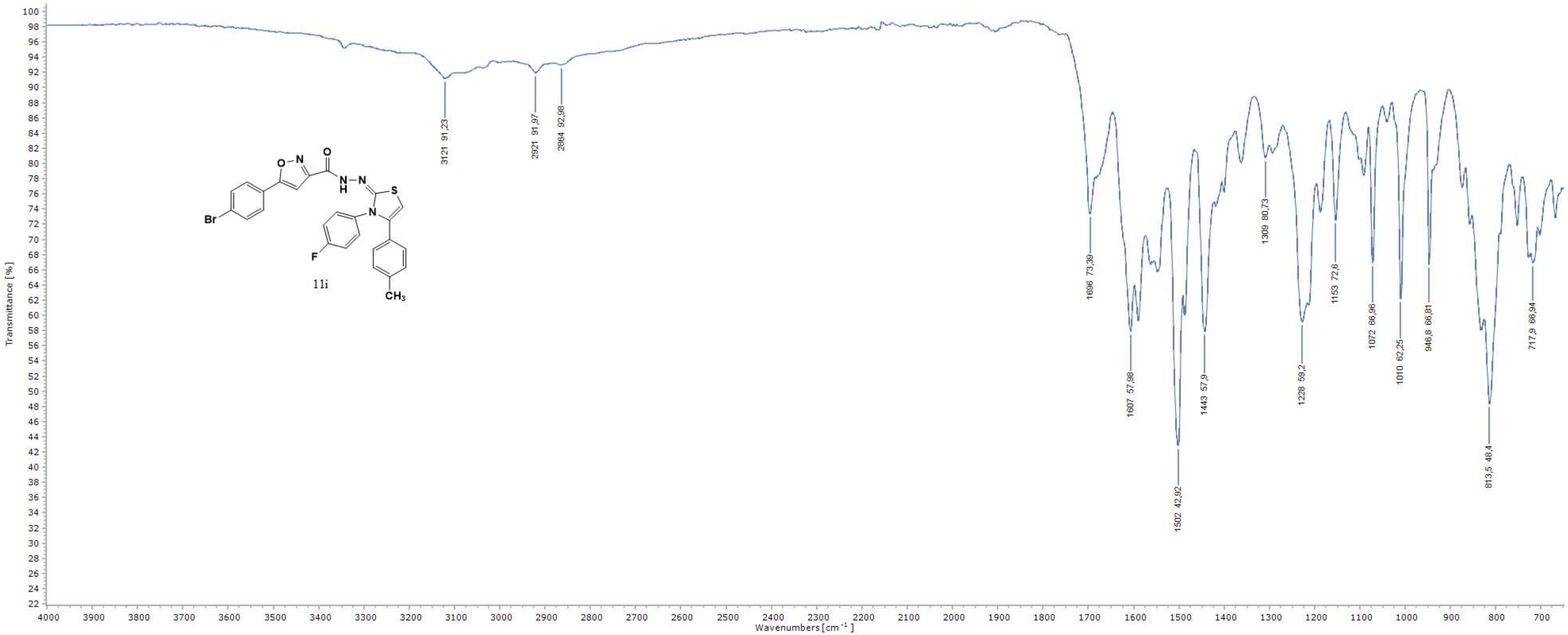

5-(4-bromophenyl)-N'-(3-(4-fluorophenyl)-4-(4-methoxyphenyl)thiazol-2(3H)-ylidene)isoxazole-3-carbohydrazide (11j):

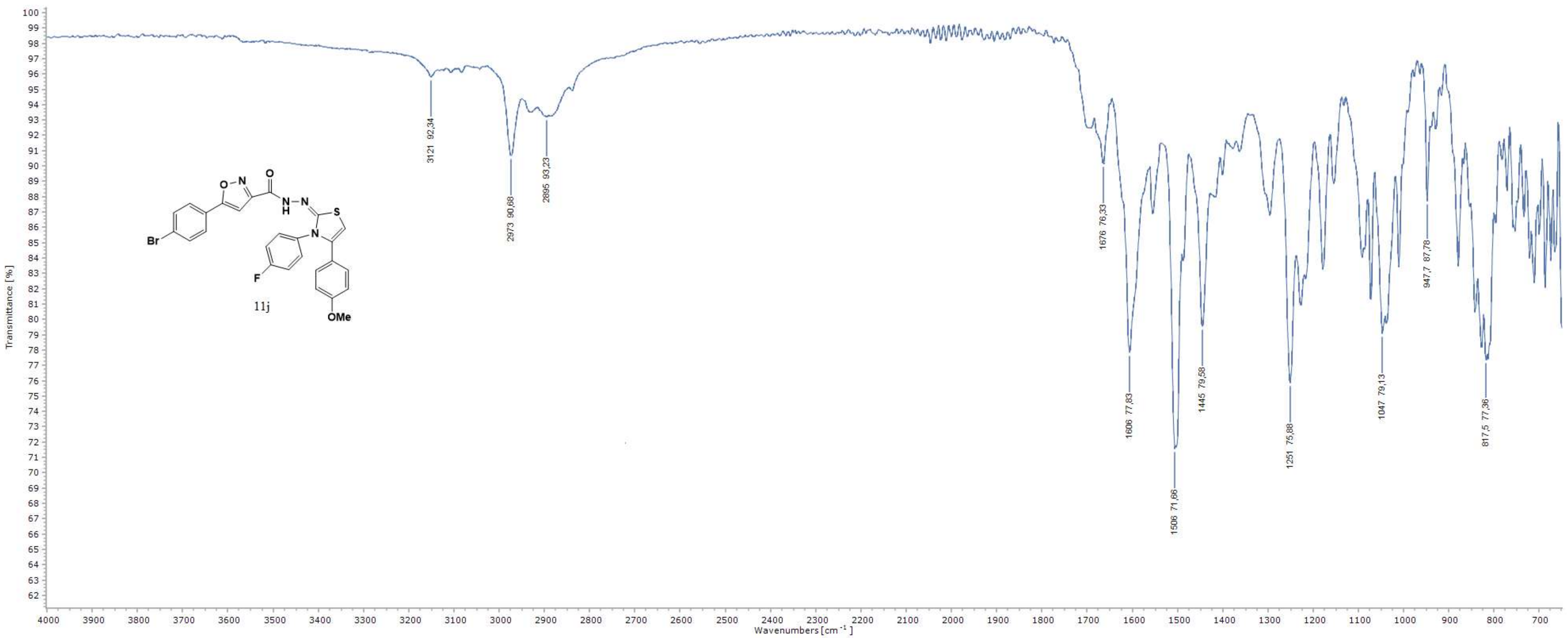

5-(3,4-bis(4-fluorophenyl)thiazol-2(3H)-ylidene)-5-(4-bromophenyl)isoxazole-3-carbohydrazide (11k):

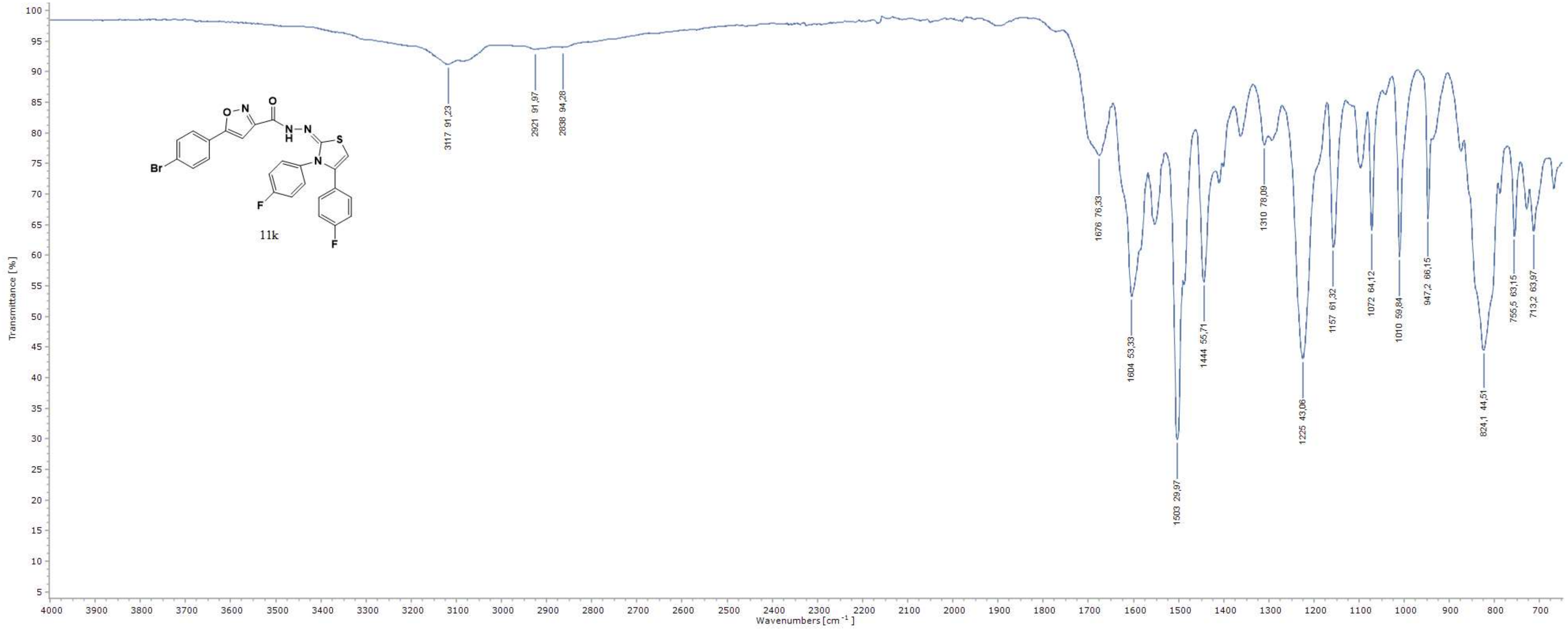

5-(4-bromophenyl)-N'-(3-(4-chlorophenyl)-4-p-tolylthiazol-2(3H)-ylidene)isoxazole-3-carbohydrazide (11l):

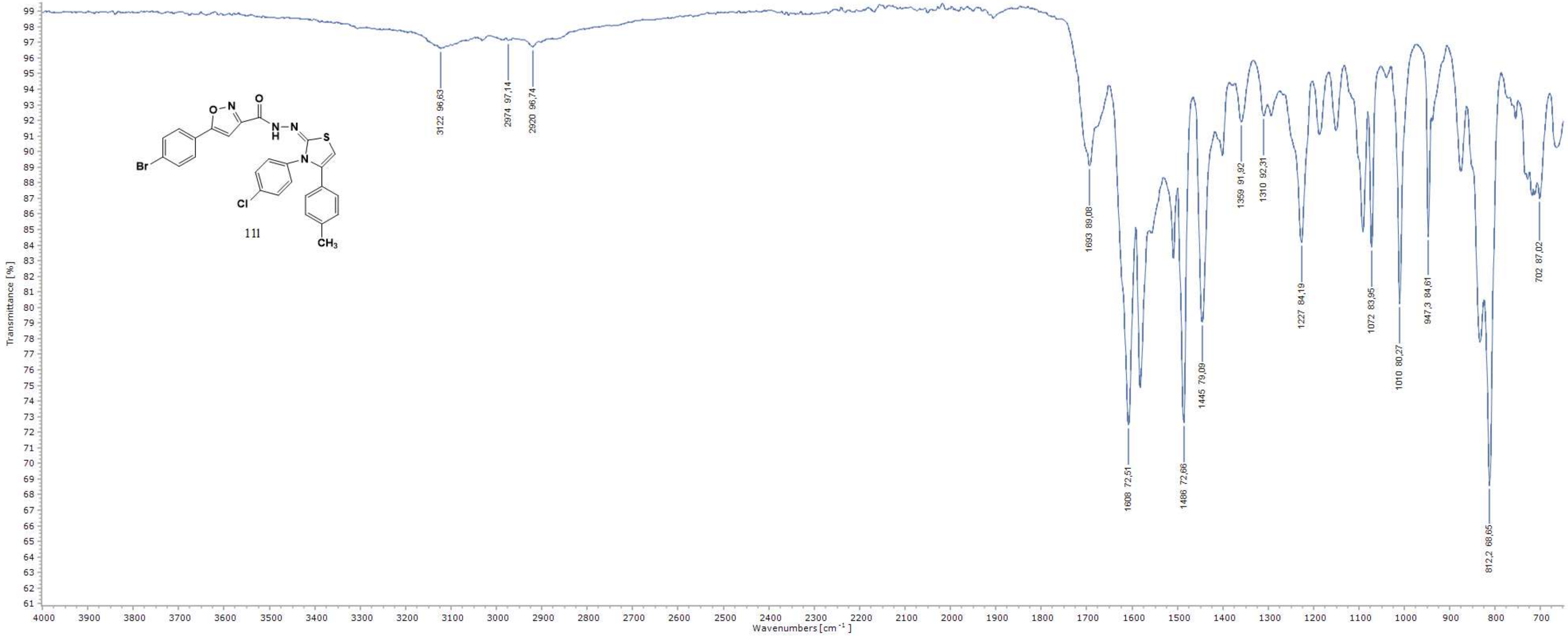

Scheme 1: NMR spectraScheme 1: NMR spectra

5-(4-bromophenyl)-N'-(3-(4-chlorophenyl)-4-(4-methoxyphenyl)thiazol-2(3H)-ylidene)isoxazole-3-carbohydrazide (11m):

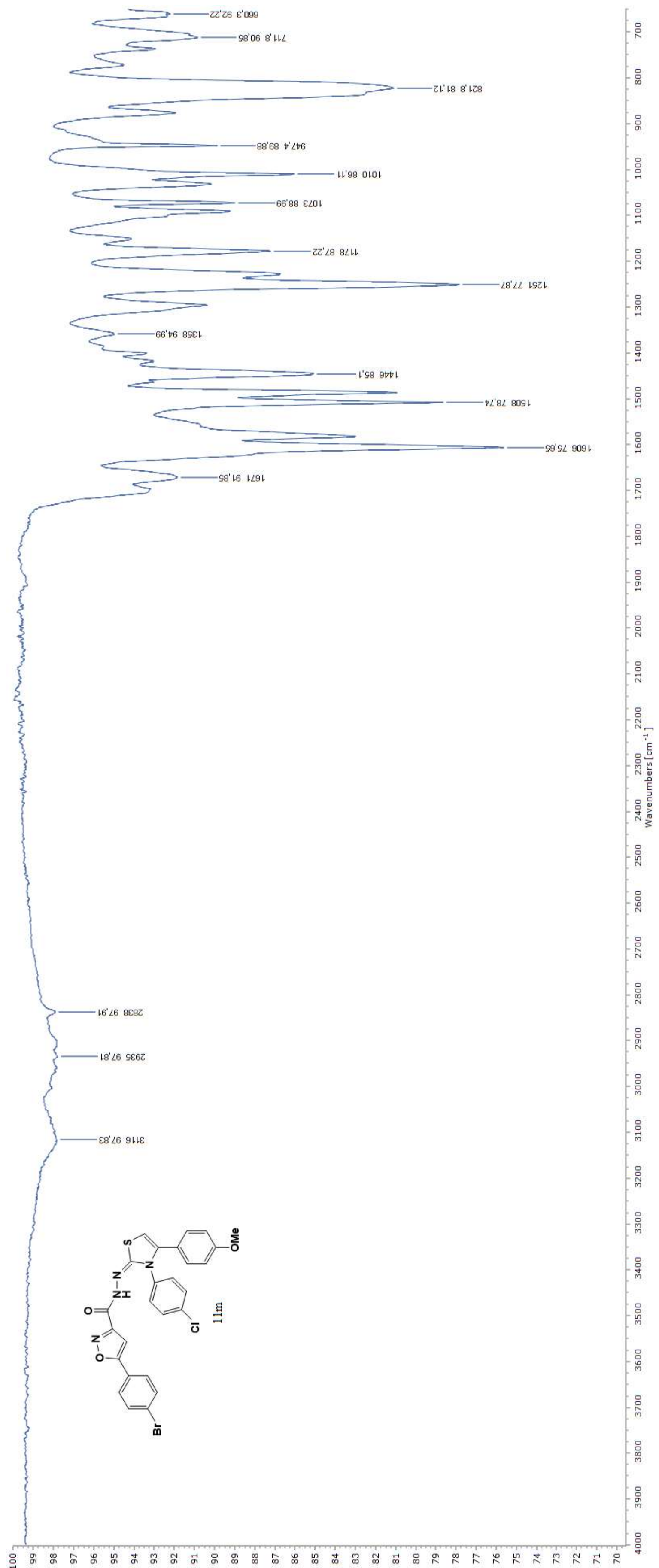

5-(4-bromophenyl)-N'-(3-(4-chlorophenyl)-4-(4-fluorophenyl)thiazol-2(3H)-ylidene)isoxazole-3-carbohydrazide (11n):

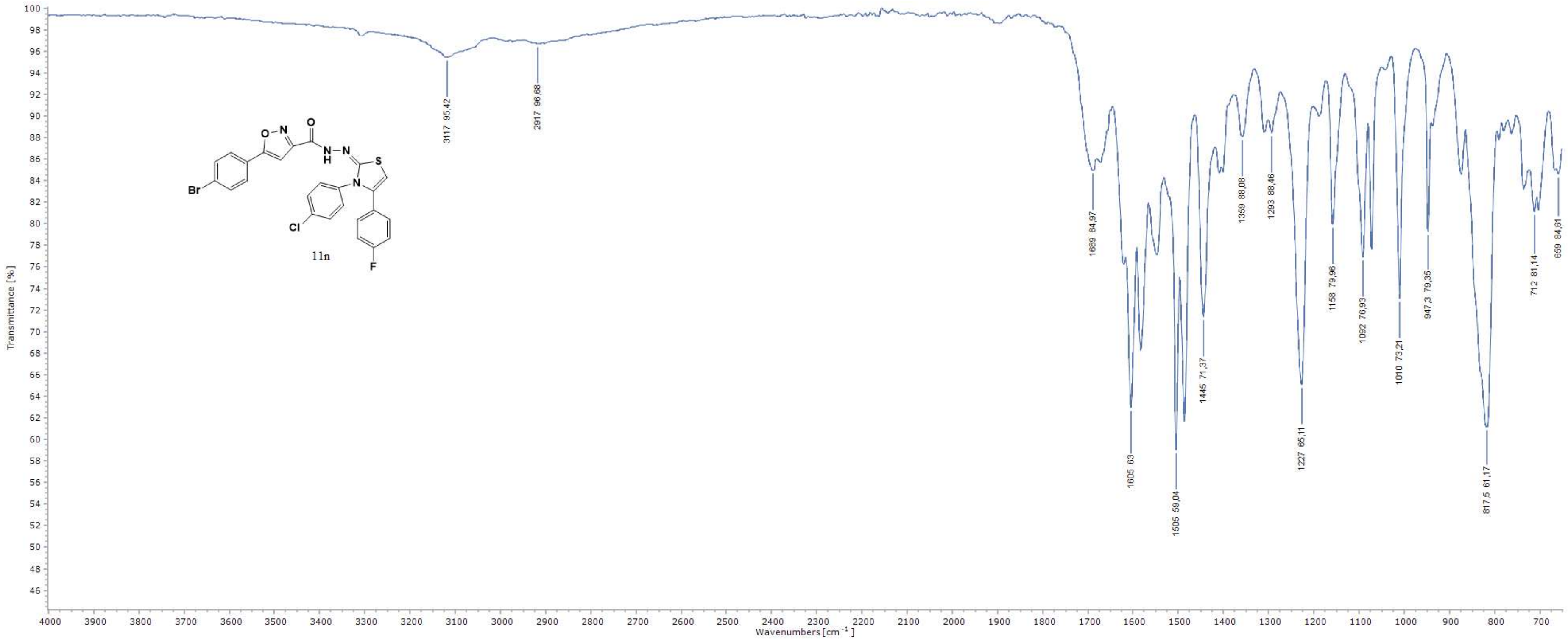

Figure 1: Inhibition graphics

CA1-II IC_{50} (HYDRATASE)

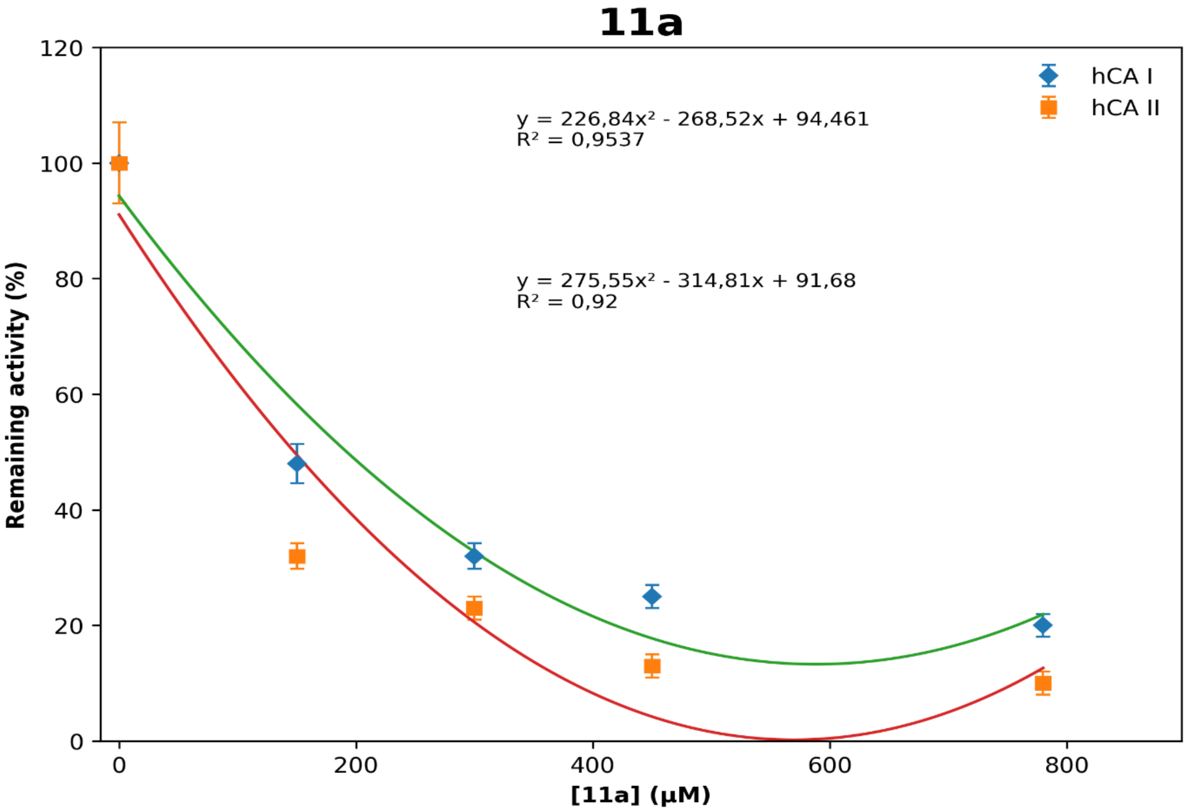

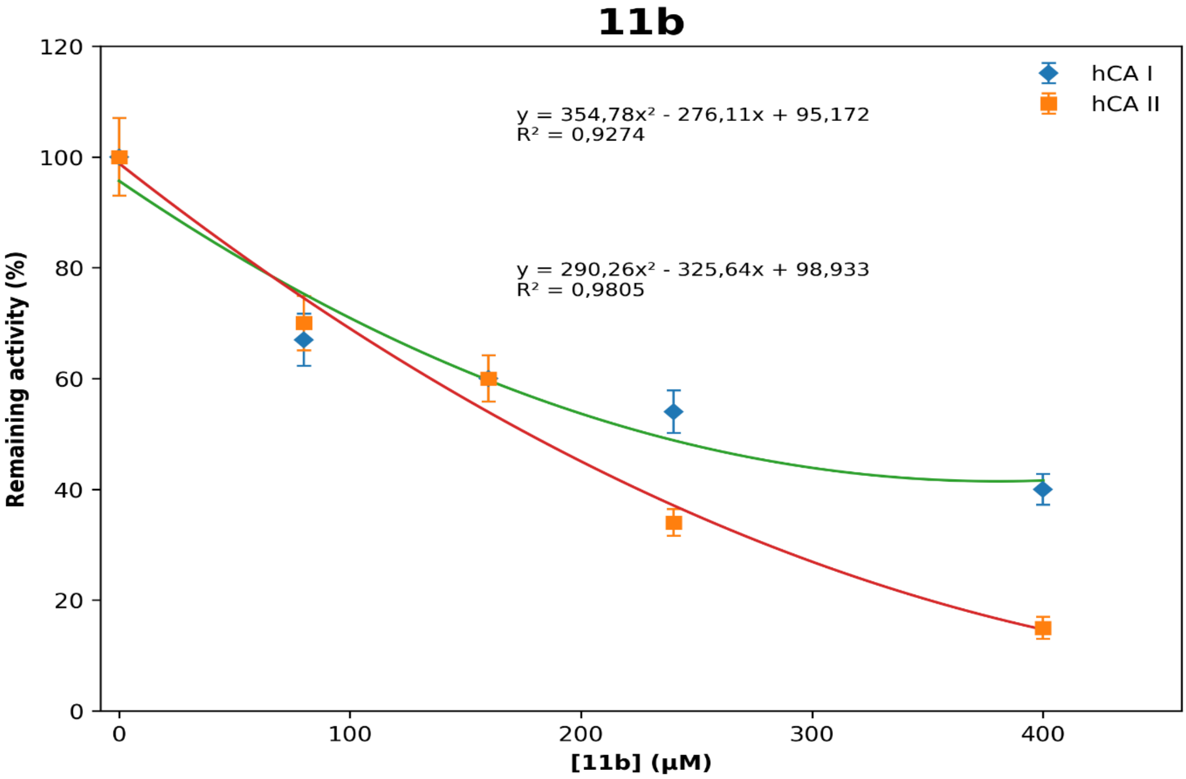

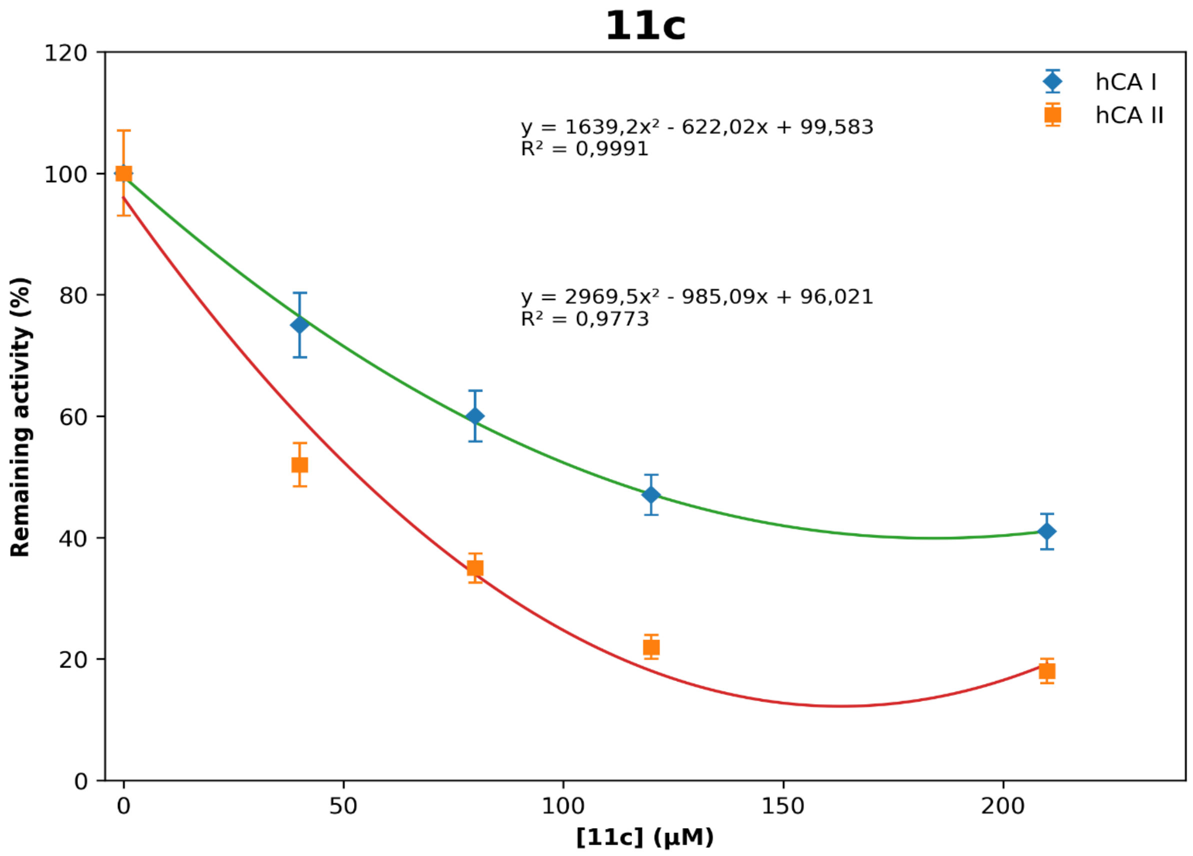

11c
hCA I
hCA II
y = 1639,2x² - 622,02x + 99,583
R² = 0,9991
y = 2969,5x² - 985,09x + 96,021
R² = 0,9773
Remaining activity (%)
[11c] (μM)

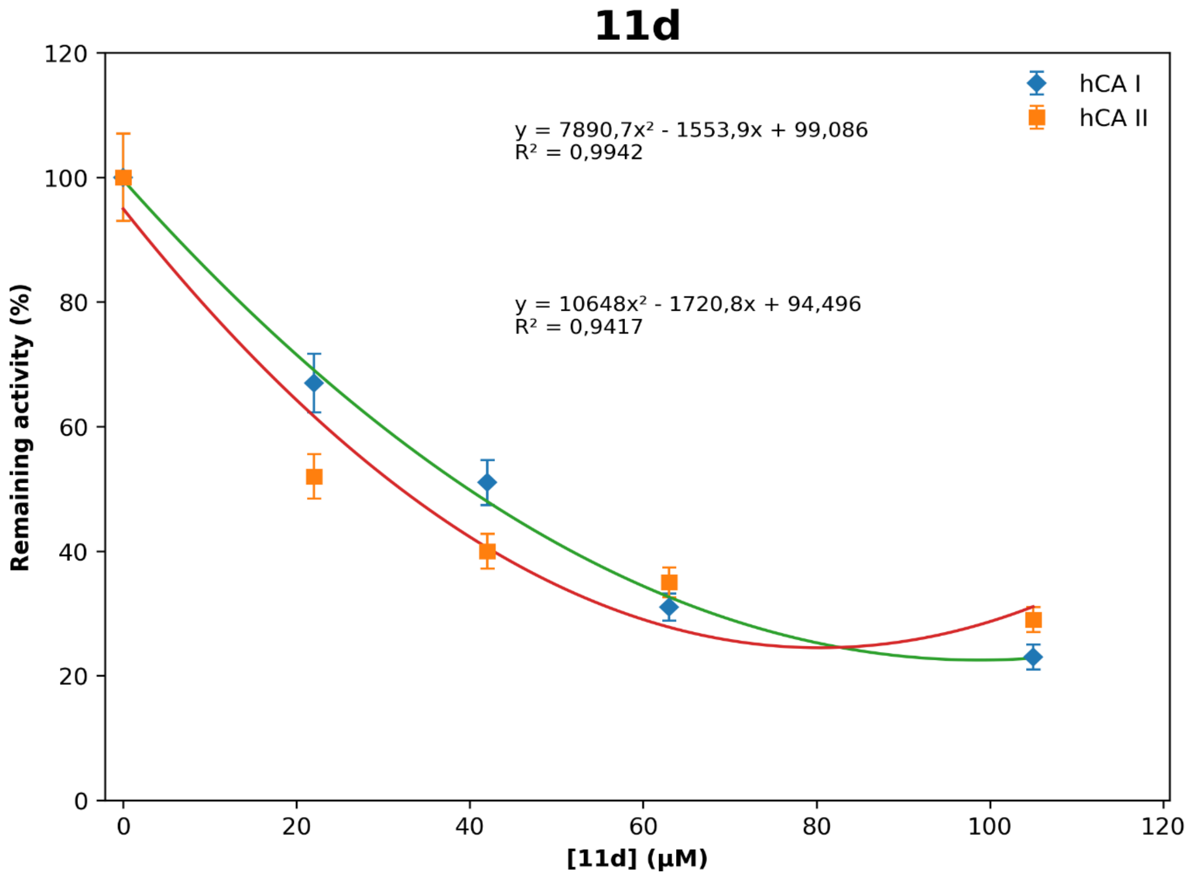

11d
hCA I
hCA II
y = 7890,7x² - 1553,9x + 99,086
R² = 0,9942
y = 10648x² - 1720,8x + 94,496
R² = 0,9417
Remaining activity (%)
[11d] (μM)

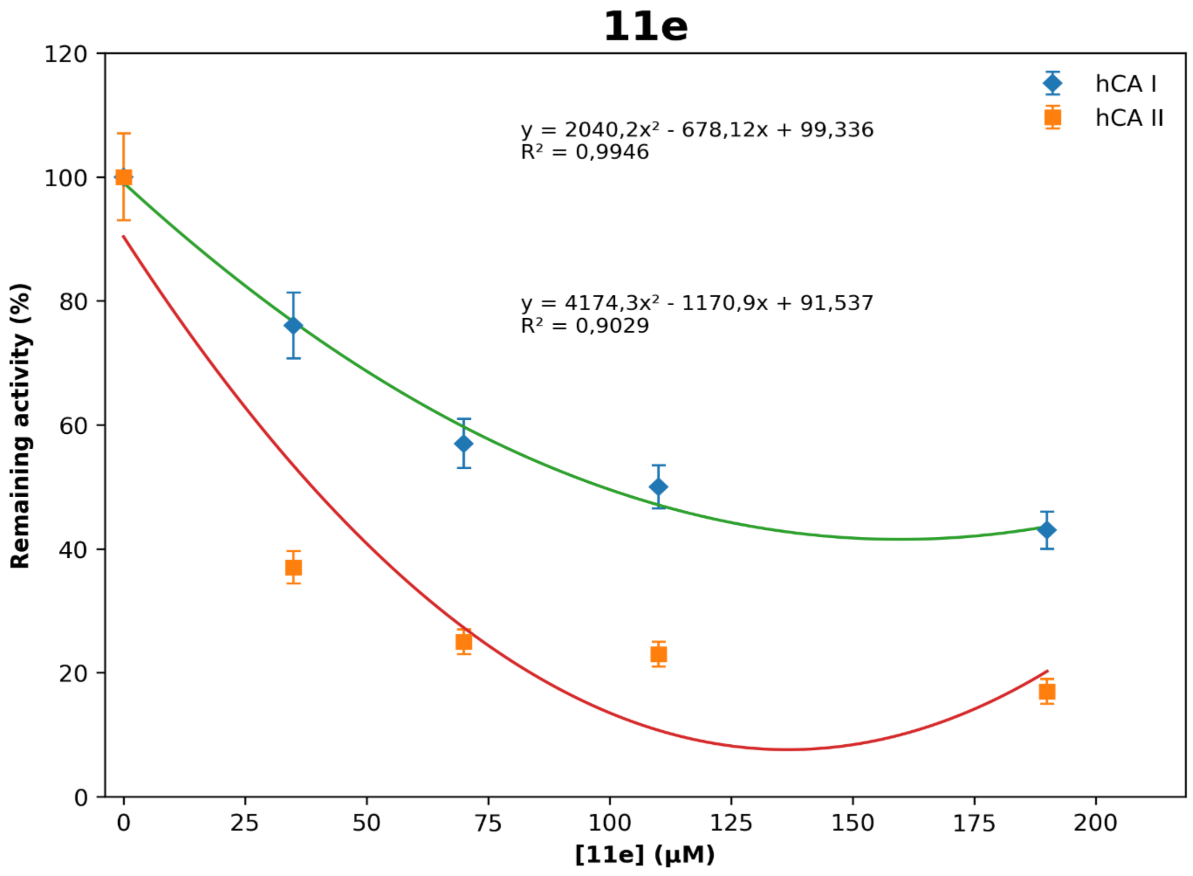
11e
hCA I
hCA II
y = 2040,2x² - 678,12x + 99,336
R² = 0,9946
y = 4174,3x² - 1170,9x + 91,537
R² = 0,9029
Remaining activity (%)
[11e] (μM)
0
20
40
60
80
100
120
0
25
50
75
100
125
150
175
200

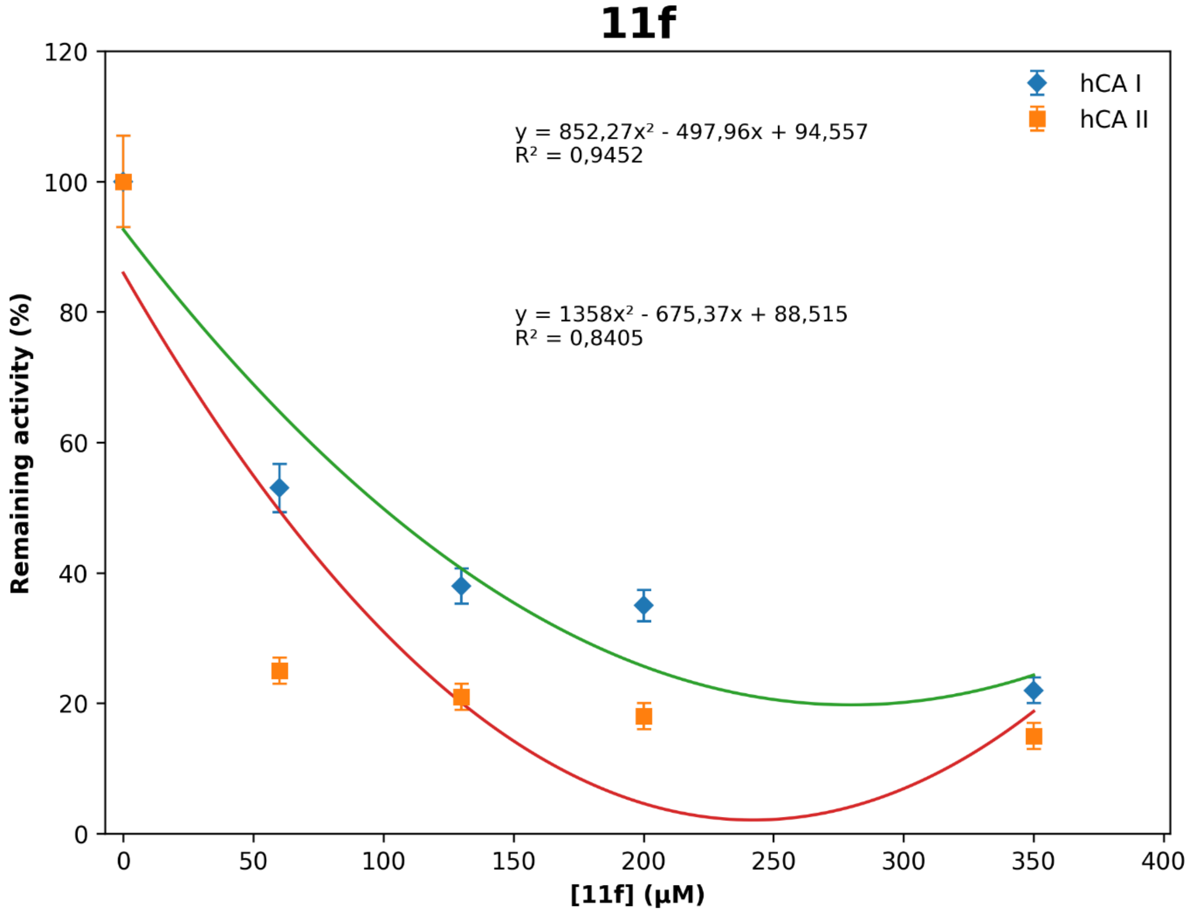
11f
hCA I
hCA II
y = 852,27x² - 497,96x + 94,557
R² = 0,9452
y = 1358x² - 675,37x + 88,515
R² = 0,8405
Remaining activity (%)
[11f] (μM)
0
20
40
60
80
100
120
0
50
100
150
200
250
300
350
400

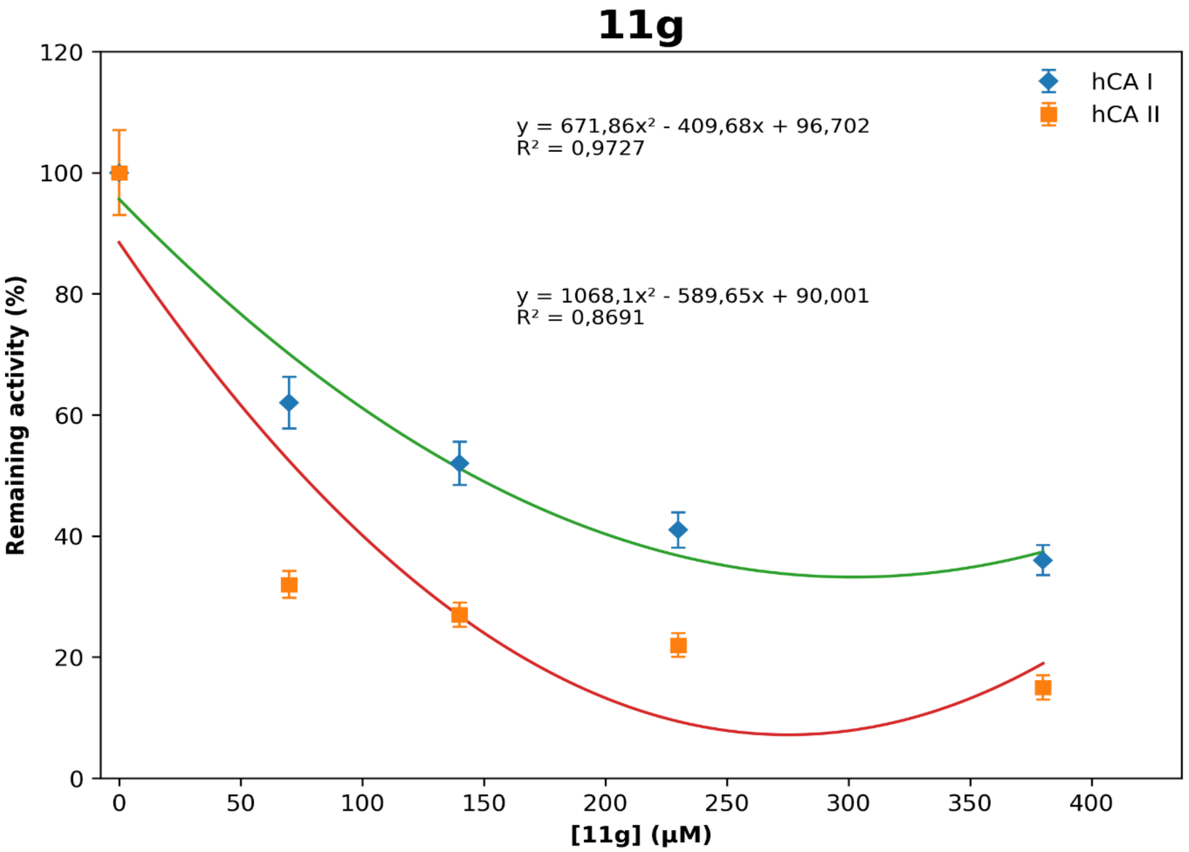
11g
hCA I
hCA II
y = 671,86x² - 409,68x + 96,702
R² = 0,9727
y = 1068,1x² - 589,65x + 90,001
R² = 0,8691
Remaining activity (%)
[11g] (μM)

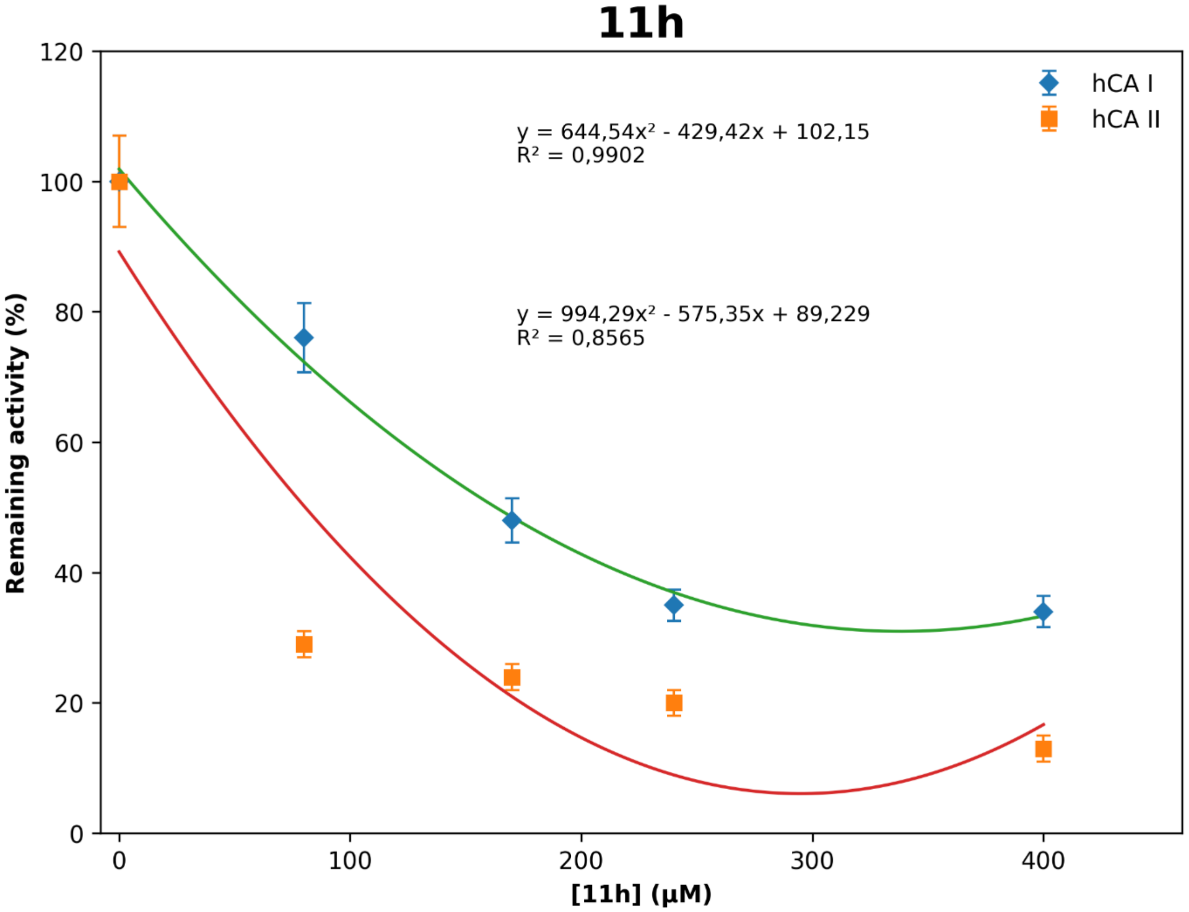
11h
hCA I
hCA II
y = 644,54x² - 429,42x + 102,15
R² = 0,9902
y = 994,29x² - 575,35x + 89,229
R² = 0,8565
Remaining activity (%)
[11h] (μM)

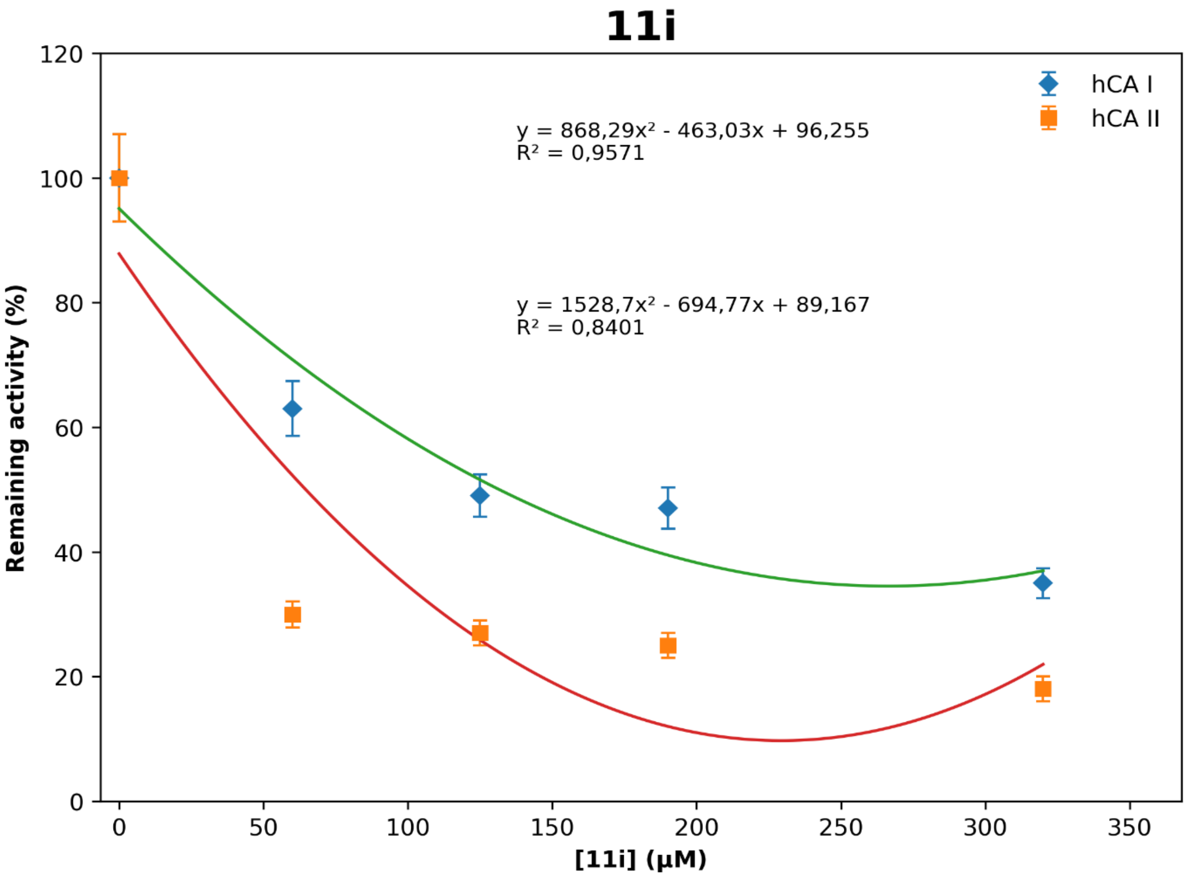
11i
Remaining activity (%)
[11i] (μM)
hCA I
hCA II
y = 868,29x² - 463,03x + 96,255
R² = 0,9571
y = 1528,7x² - 694,77x + 89,167
R² = 0,8401

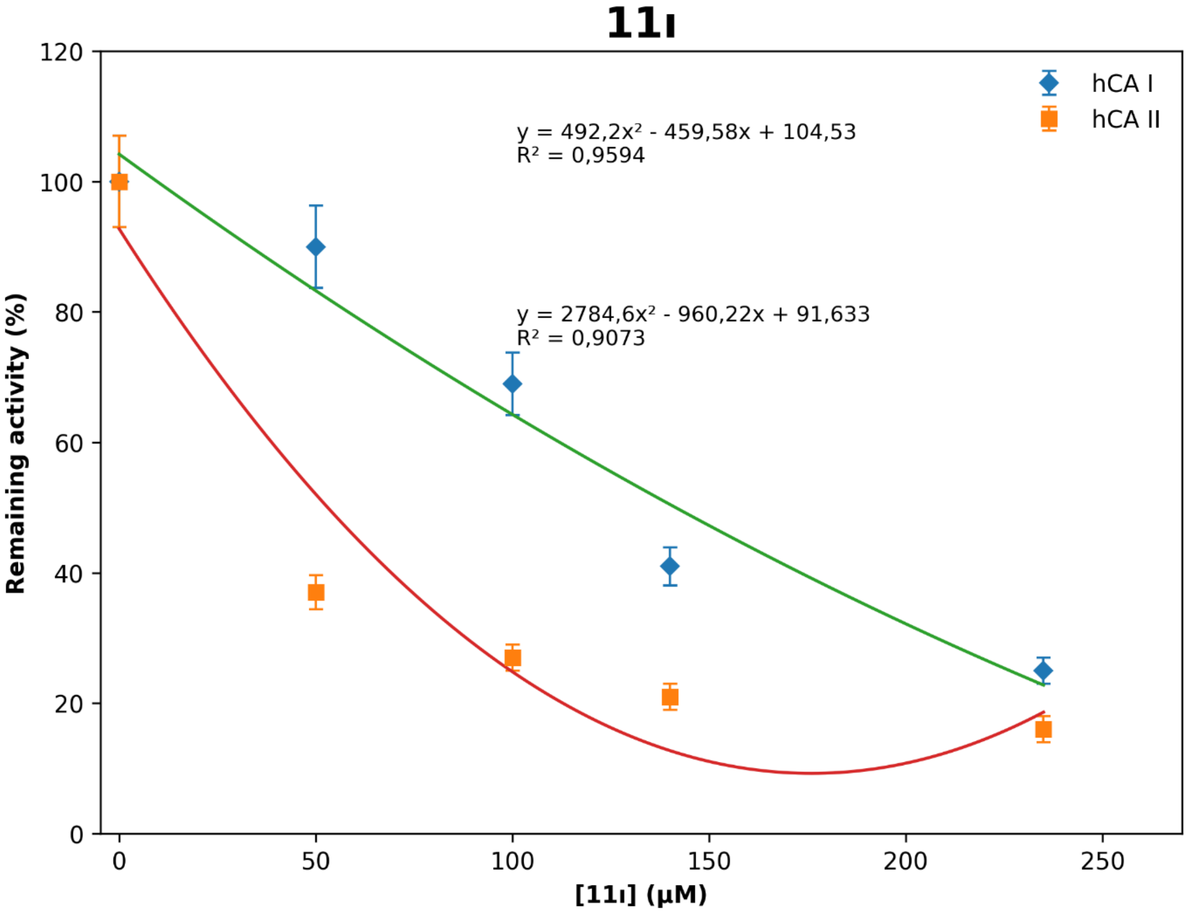
11ı
Remaining activity (%)
[11ı] (μM)
hCA I
hCA II
y = 492,2x² - 459,58x + 104,53
R² = 0,9594
y = 2784,6x² - 960,22x + 91,633
R² = 0,9073

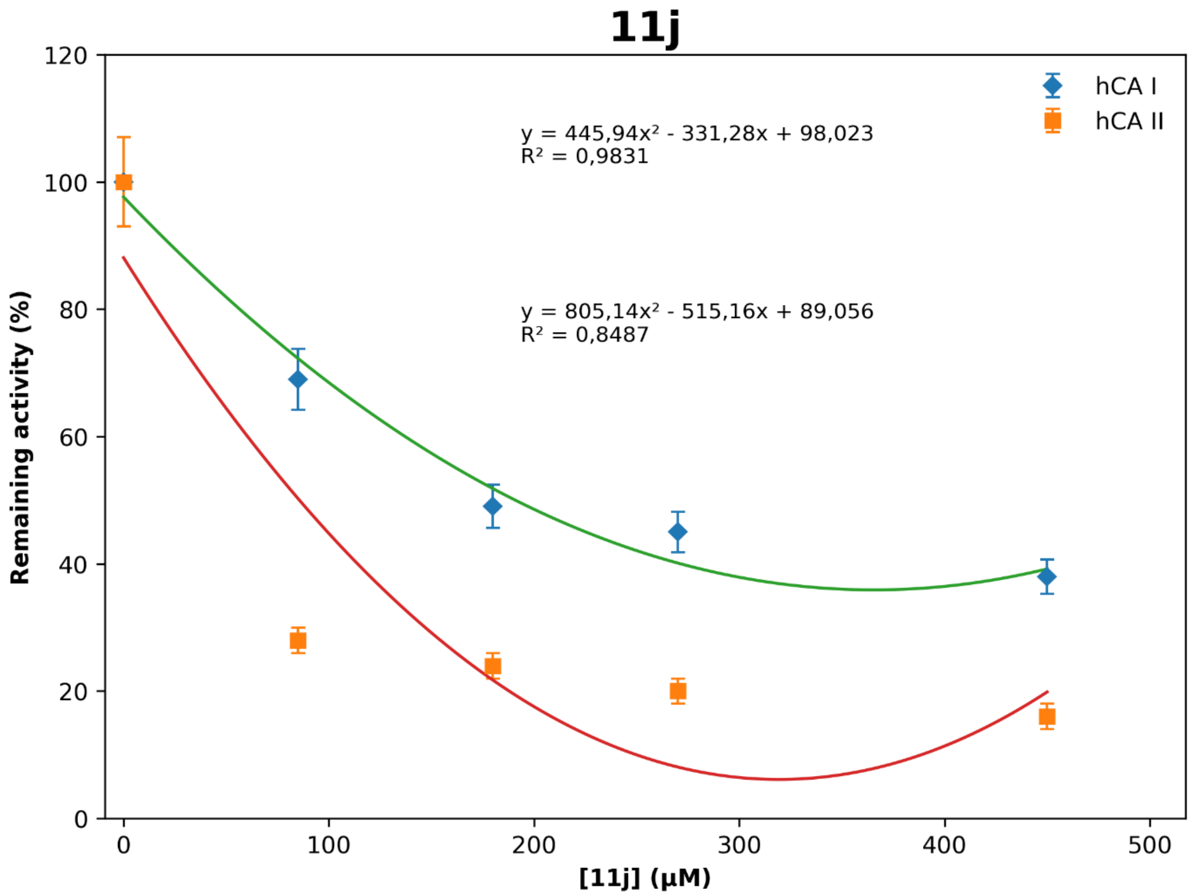
11j
hCA I
hCA II
y = 445,94x² - 331,28x + 98,023
R² = 0,9831
y = 805,14x² - 515,16x + 89,056
R² = 0,8487
Remaining activity (%)
[11j] (µM)
0
20
40
60
80
100
120
0
100
200
300
400
500

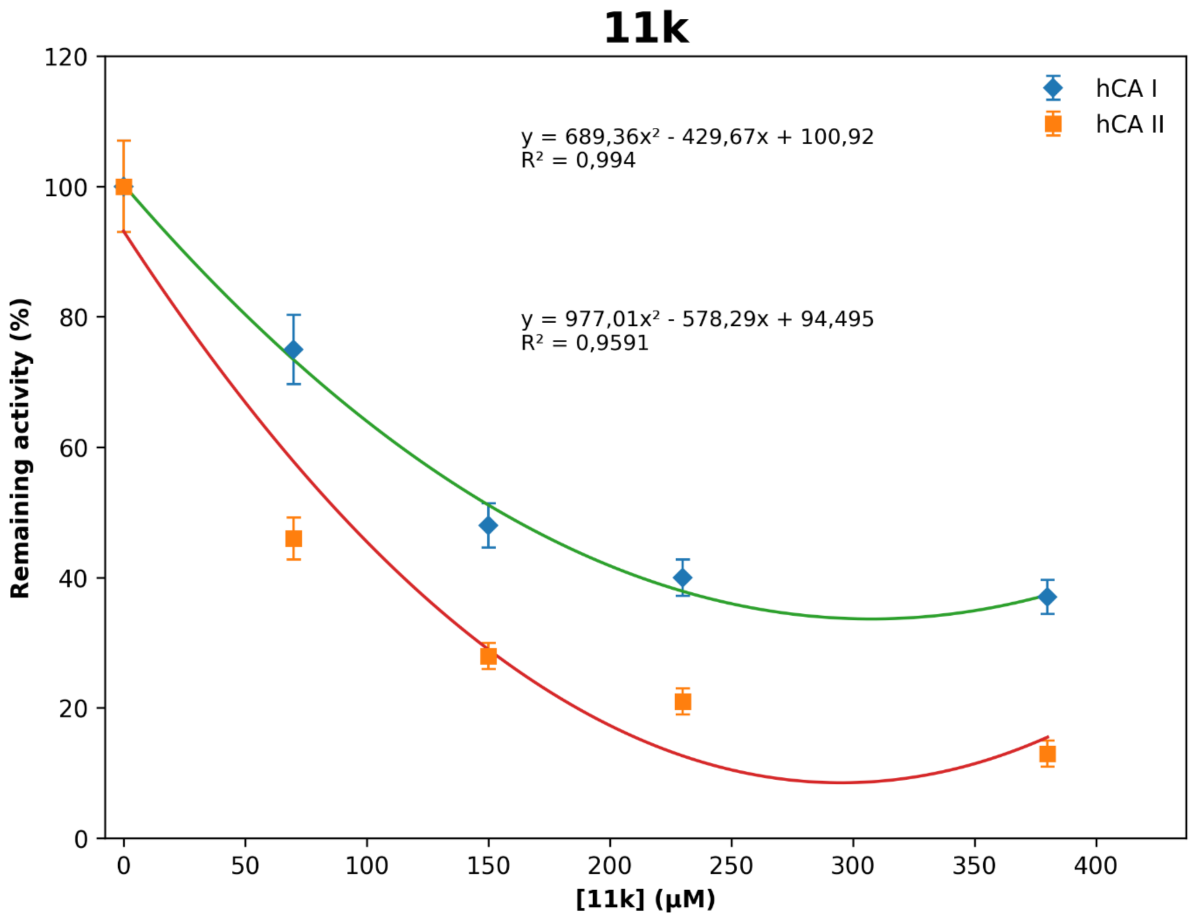
11k
hCA I
hCA II
y = 689,36x² - 429,67x + 100,92
R² = 0,994
y = 977,01x² - 578,29x + 94,495
R² = 0,9591
Remaining activity (%)
[11k] (µM)
0
20
40
60
80
100
120
0
50
100
150
200
250
300
350
400

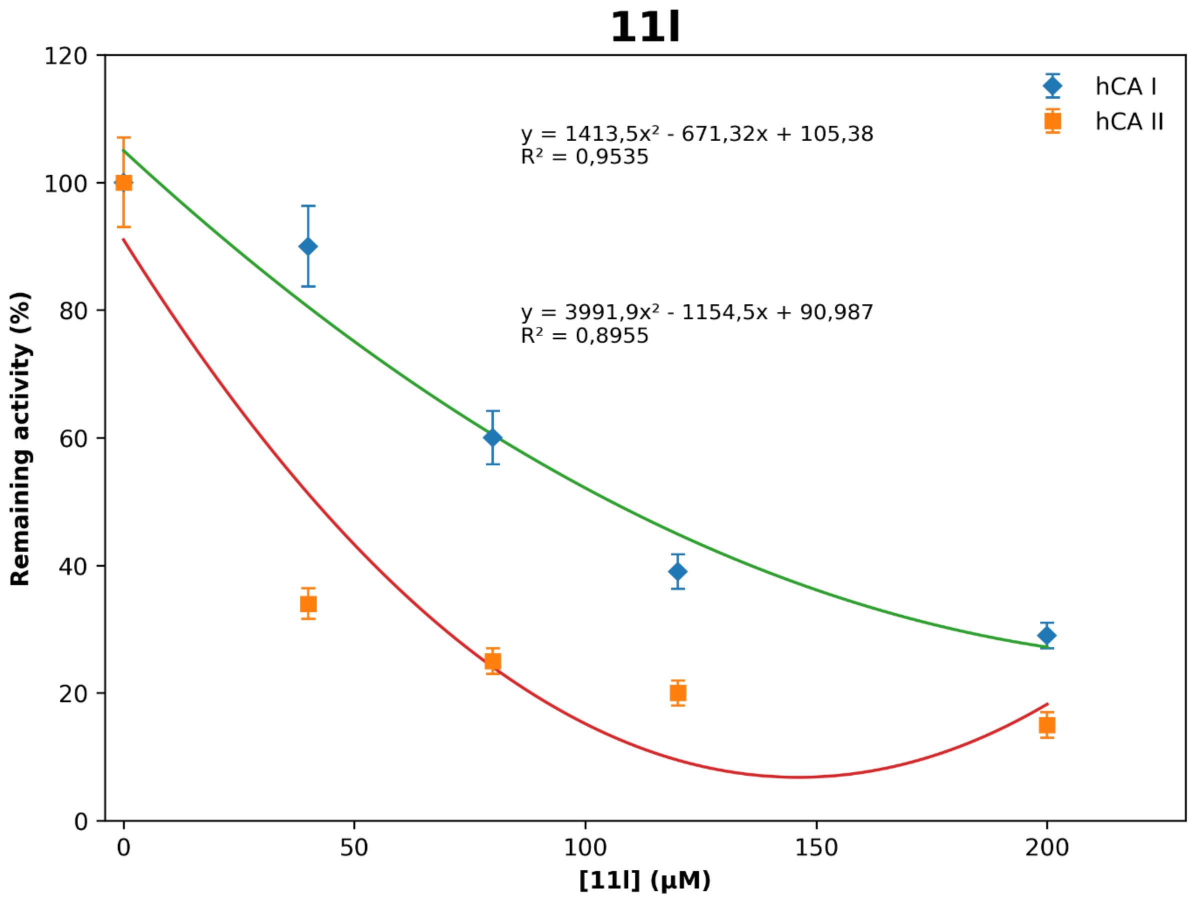
11l
hCA I
hCA II
y = 1413,5x² - 671,32x + 105,38
R² = 0,9535
y = 3991,9x² - 1154,5x + 90,987
R² = 0,8955
Remaining activity (%)
[11l] (μM)
0
20
40
60
80
100
120
0
50
100
150
200

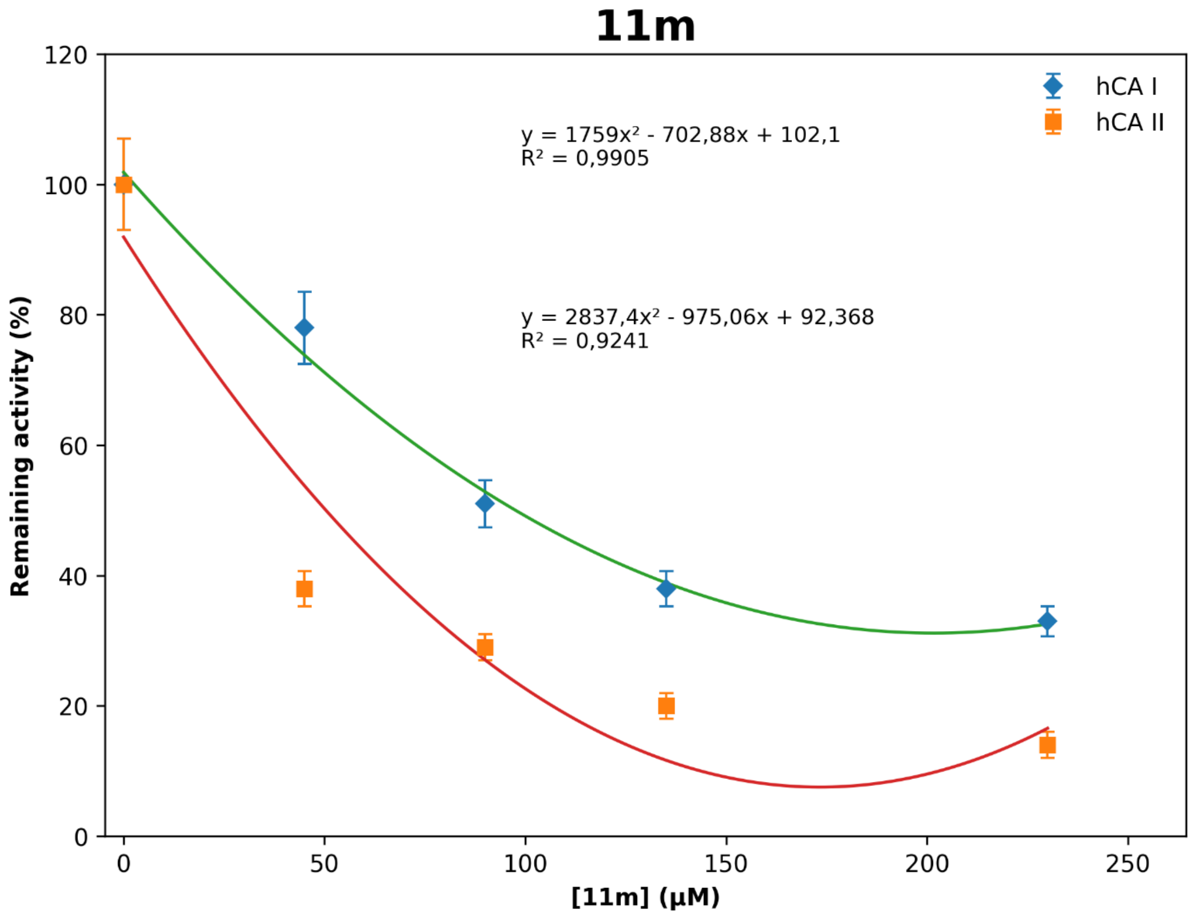
11m
hCA I
hCA II
y = 1759x² - 702,88x + 102,1
R² = 0,9905
y = 2837,4x² - 975,06x + 92,368
R² = 0,9241
Remaining activity (%)
[11m] (μM)
0
20
40
60
80
100
120
0
50
100
150
200
250

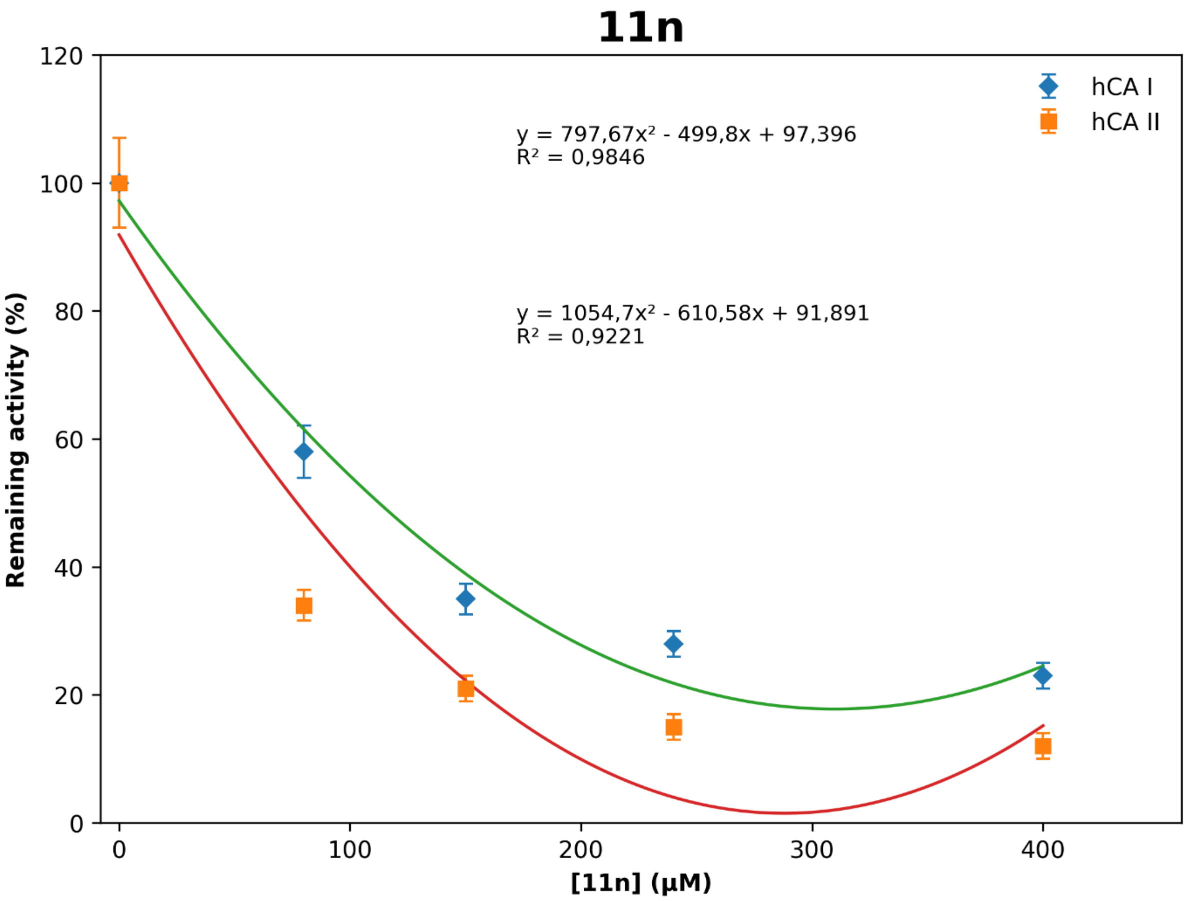

11n
120
100
80
60
40
20
0
0
100
200
300
400
Remaining activity (%)
[11n] (μM)
hCA I
hCA II
y = 797,67x² - 499,8x + 97,396
R² = 0,9846
y = 1054,7x² - 610,58x + 91,891
R² = 0,9221

CA I-II IC_{50} (ESTERASE)

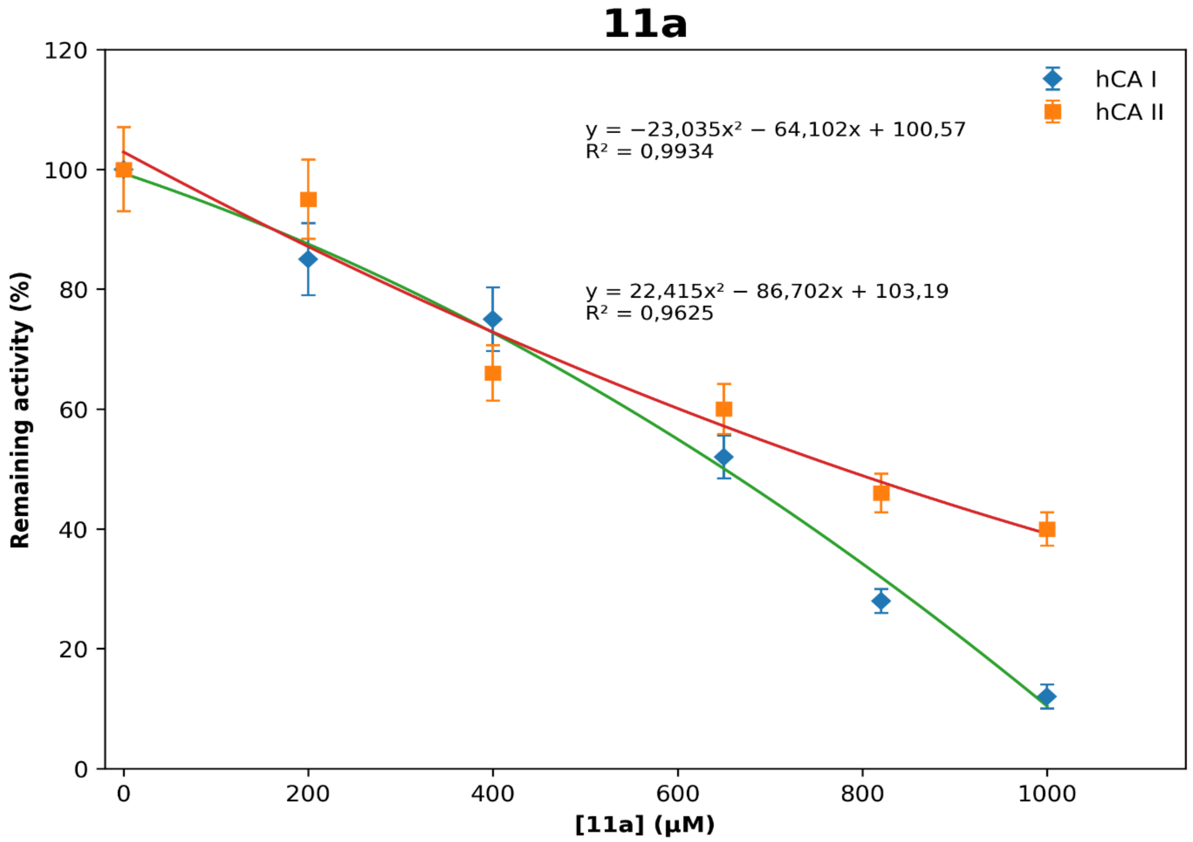

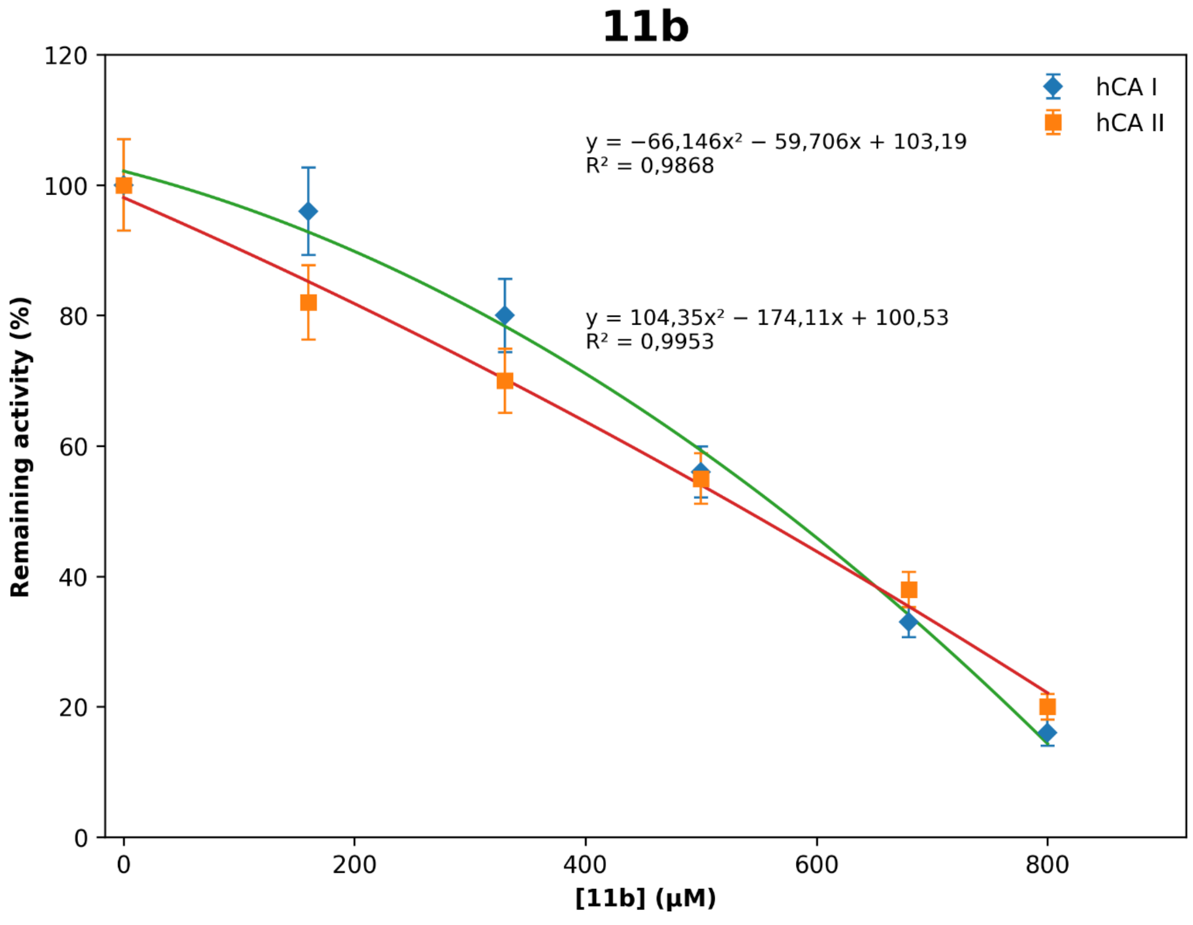

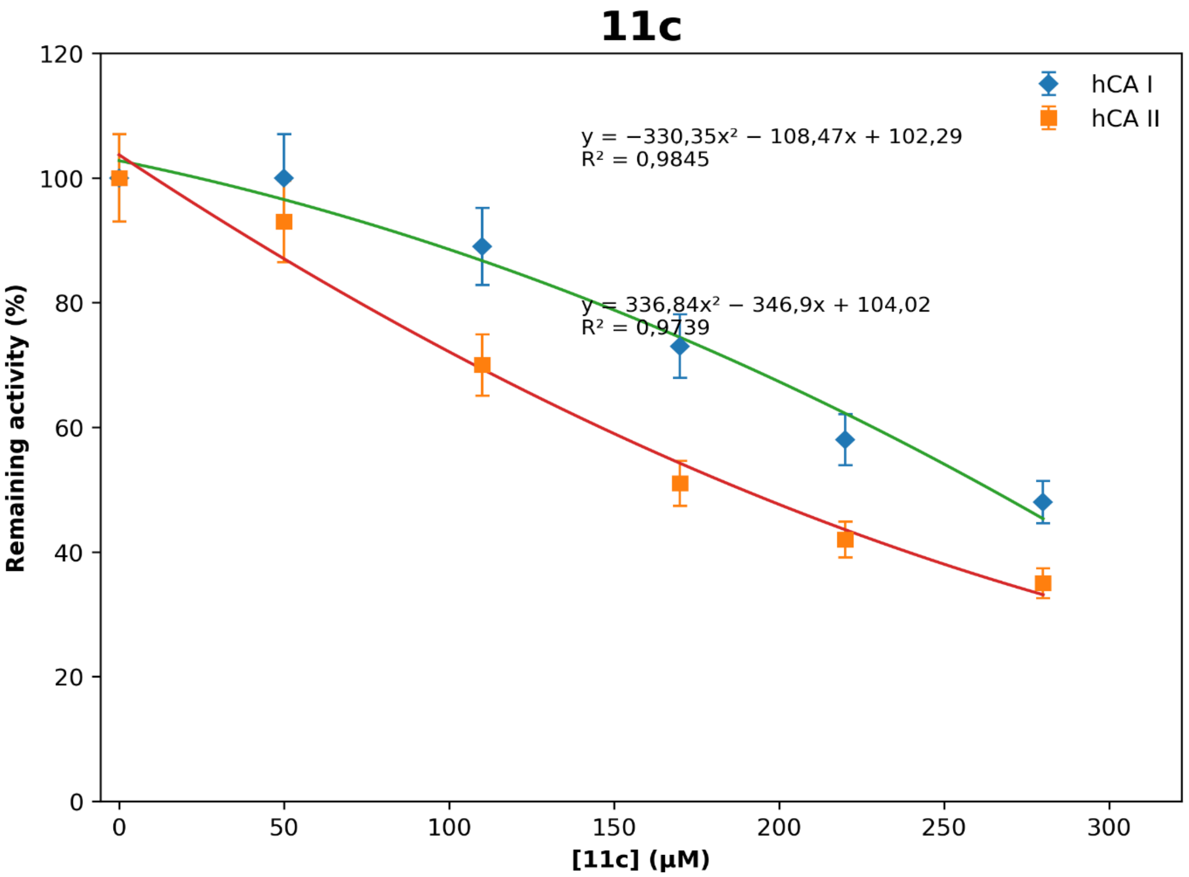
11c
hCA I
hCA II
y = −330,35x² − 108,47x + 102,29
R² = 0,9845
y = 336,84x² − 346,9x + 104,02
R² = 0,9739
Remaining activity (%)
[11c] (µM)
0
20
40
60
80
100
120
0
50
100
150
200
250
300

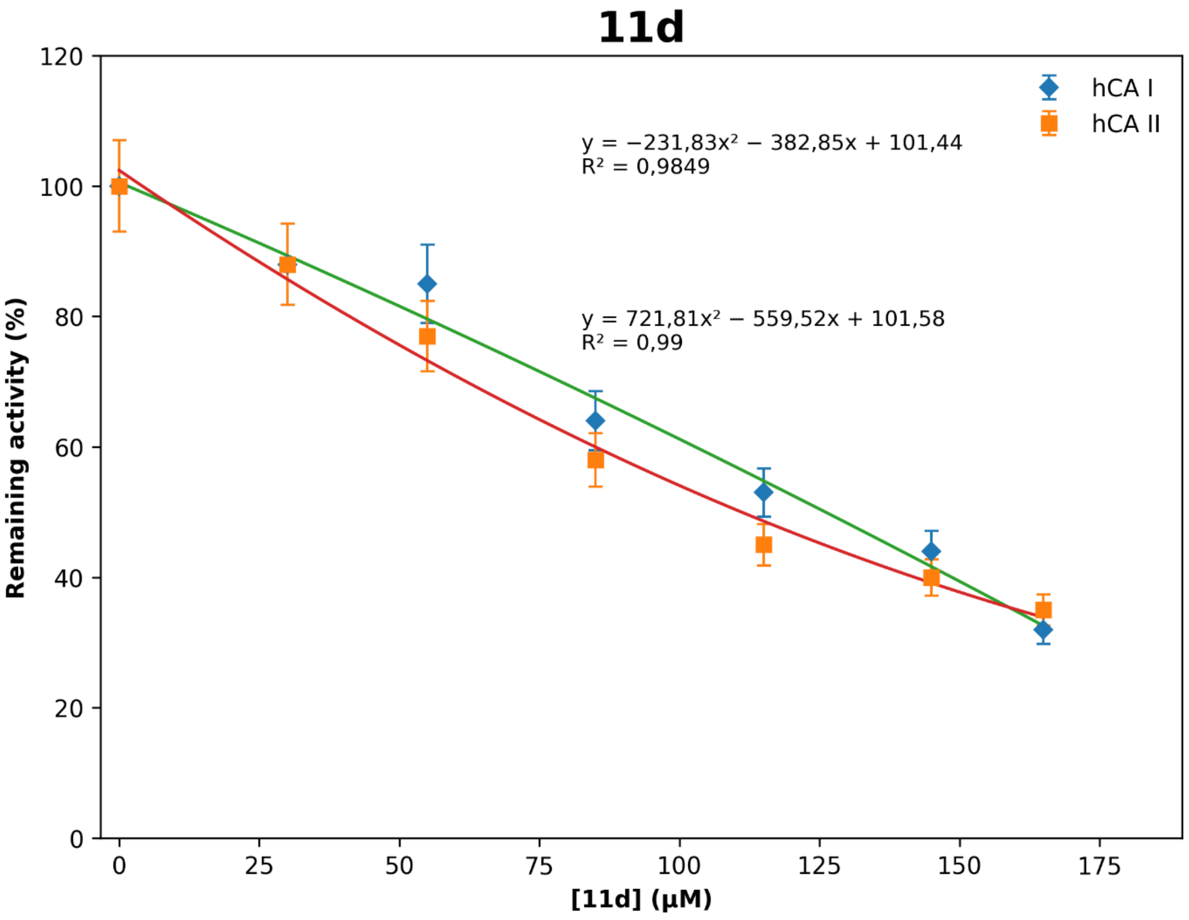
11d
hCA I
hCA II
y = −231,83x² − 382,85x + 101,44
R² = 0,9849
y = 721,81x² − 559,52x + 101,58
R² = 0,99
Remaining activity (%)
[11d] (µM)
0
20
40
60
80
100
120
0
25
50
75
100
125
150
175

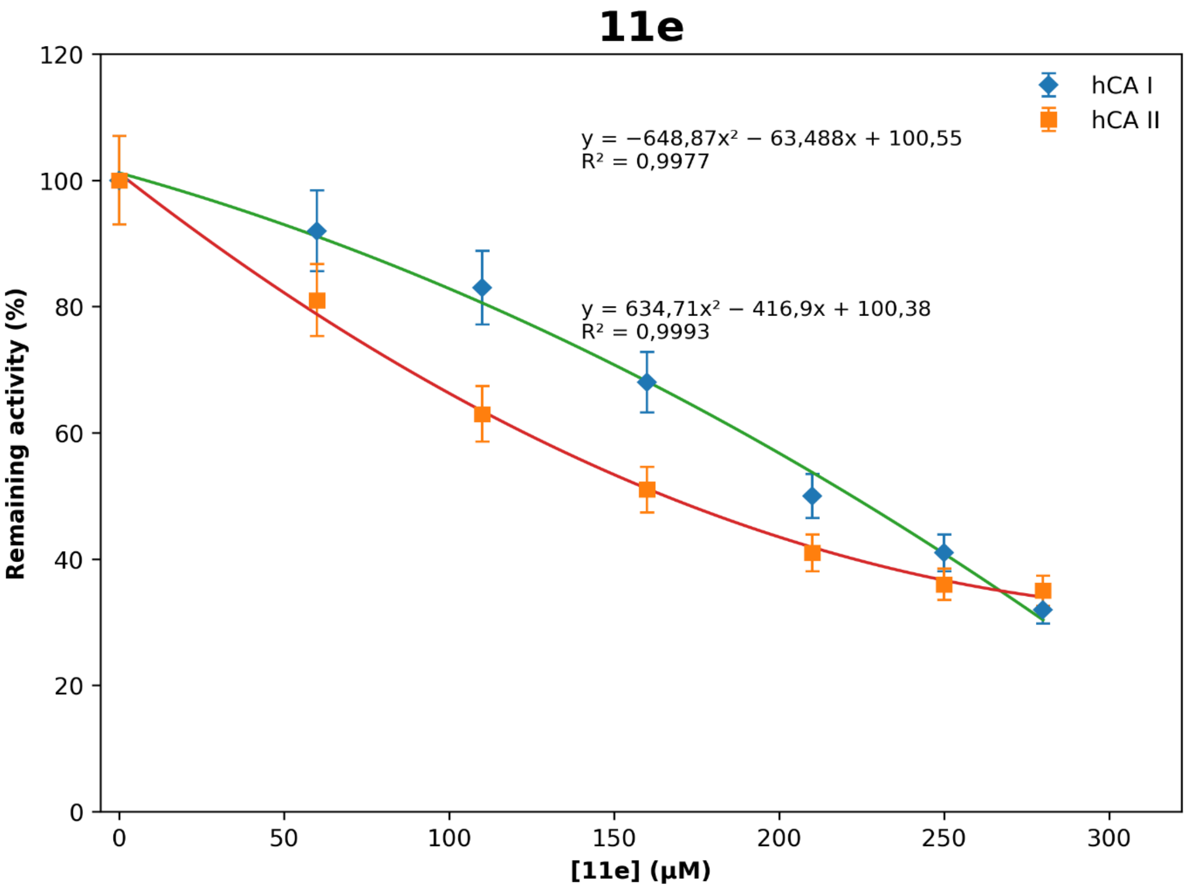
11e
hCA I
hCA II
y = −648,87x² − 63,488x + 100,55
R² = 0,9977
y = 634,71x² − 416,9x + 100,38
R² = 0,9993
Remaining activity (%)
[11e] (μM)

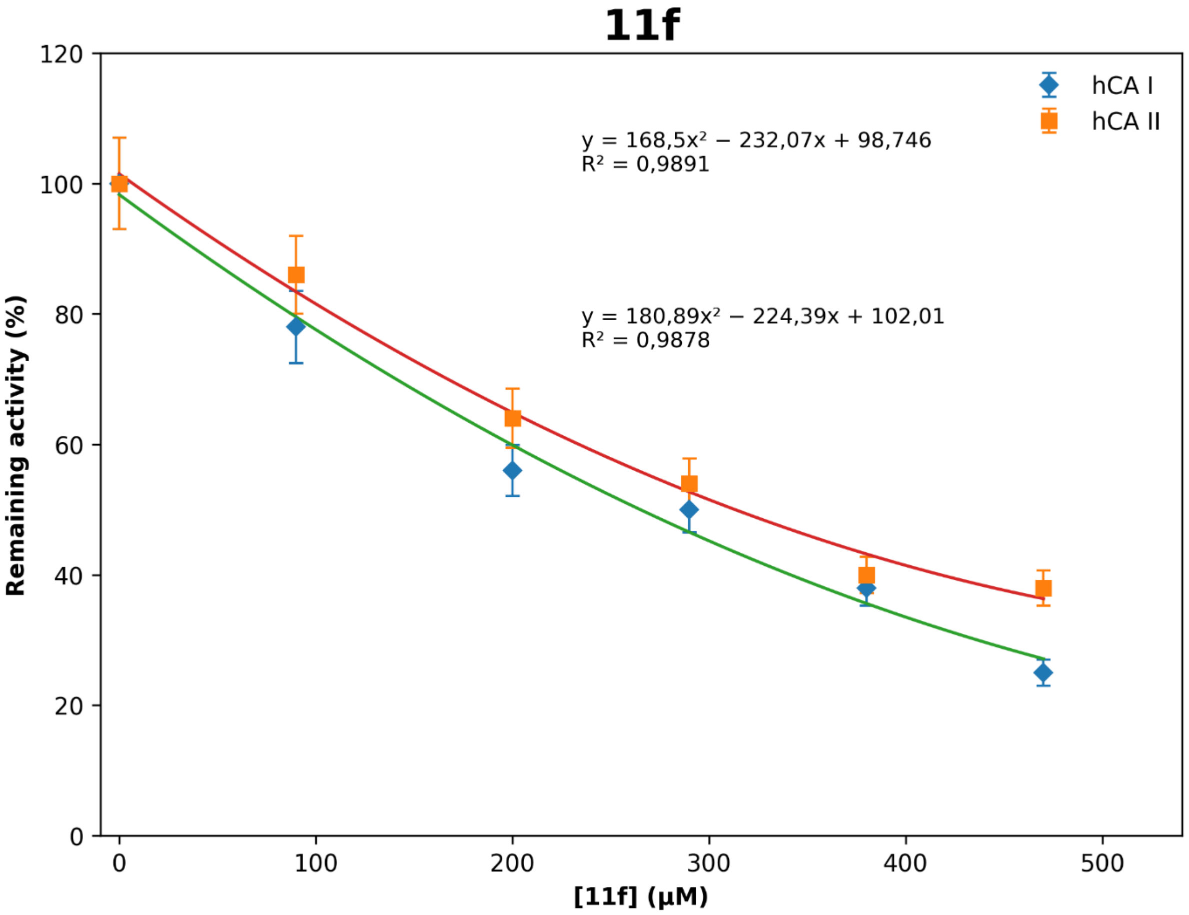
11f
hCA I
hCA II
y = 168,5x² − 232,07x + 98,746
R² = 0,9891
y = 180,89x² − 224,39x + 102,01
R² = 0,9878
Remaining activity (%)
[11f] (μM)

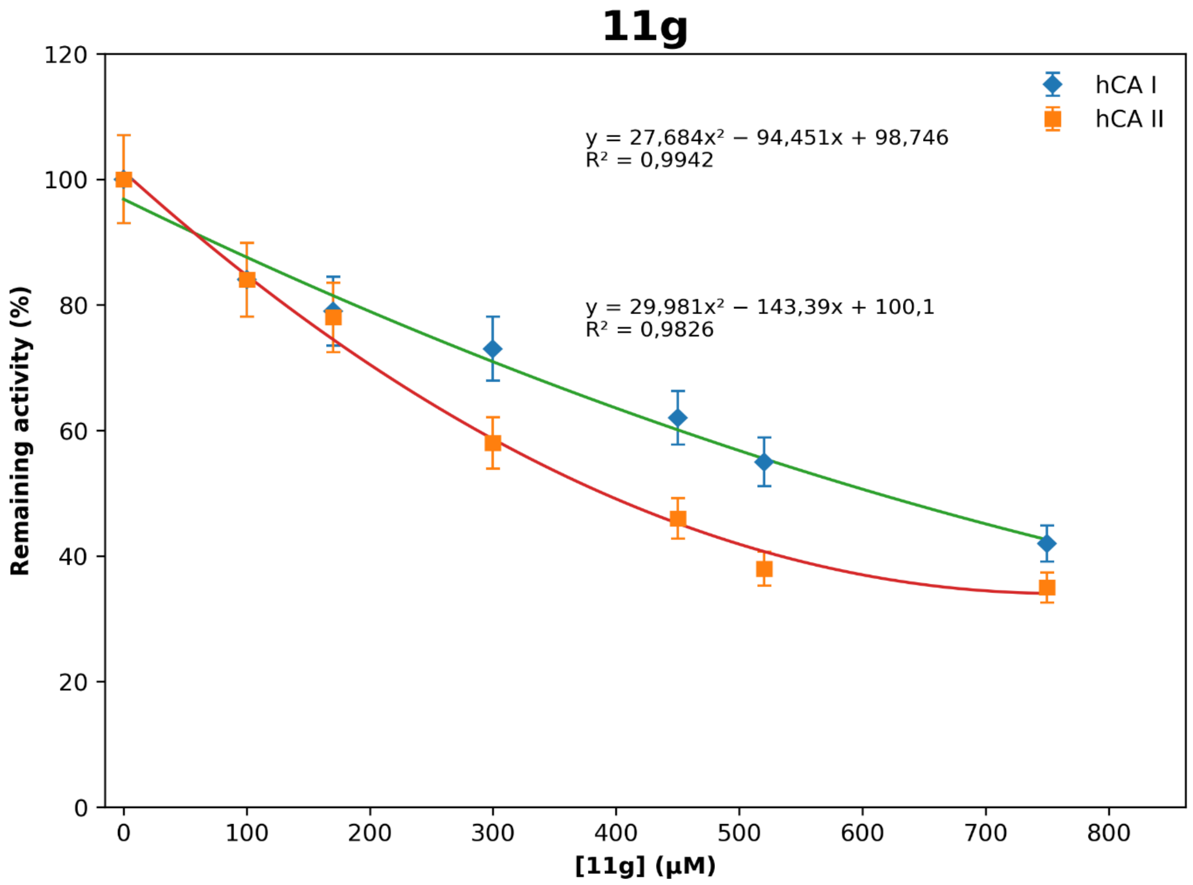
11g
Remaining activity (%)
[11g] (μM)
hCA I
hCA II
y = 27,684x² − 94,451x + 98,746
R² = 0,9942
y = 29,981x² − 143,39x + 100,1
R² = 0,9826
0
20
40
60
80
100
120
0
100
200
300
400
500
600
700
800

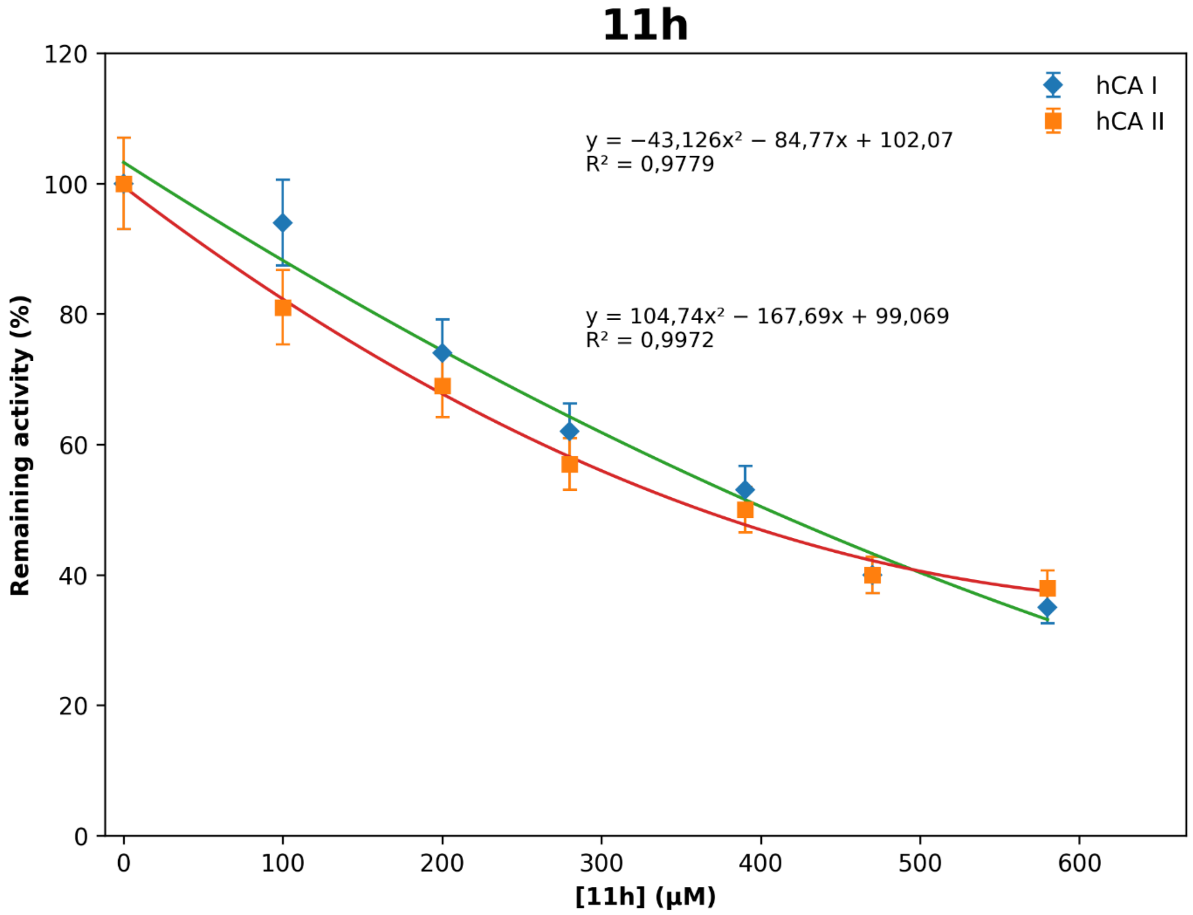
11h
Remaining activity (%)
[11h] (μM)
hCA I
hCA II
y = −43,126x² − 84,77x + 102,07
R² = 0,9779
y = 104,74x² − 167,69x + 99,069
R² = 0,9972
0
20
40
60
80
100
120
0
100
200
300
400
500
600

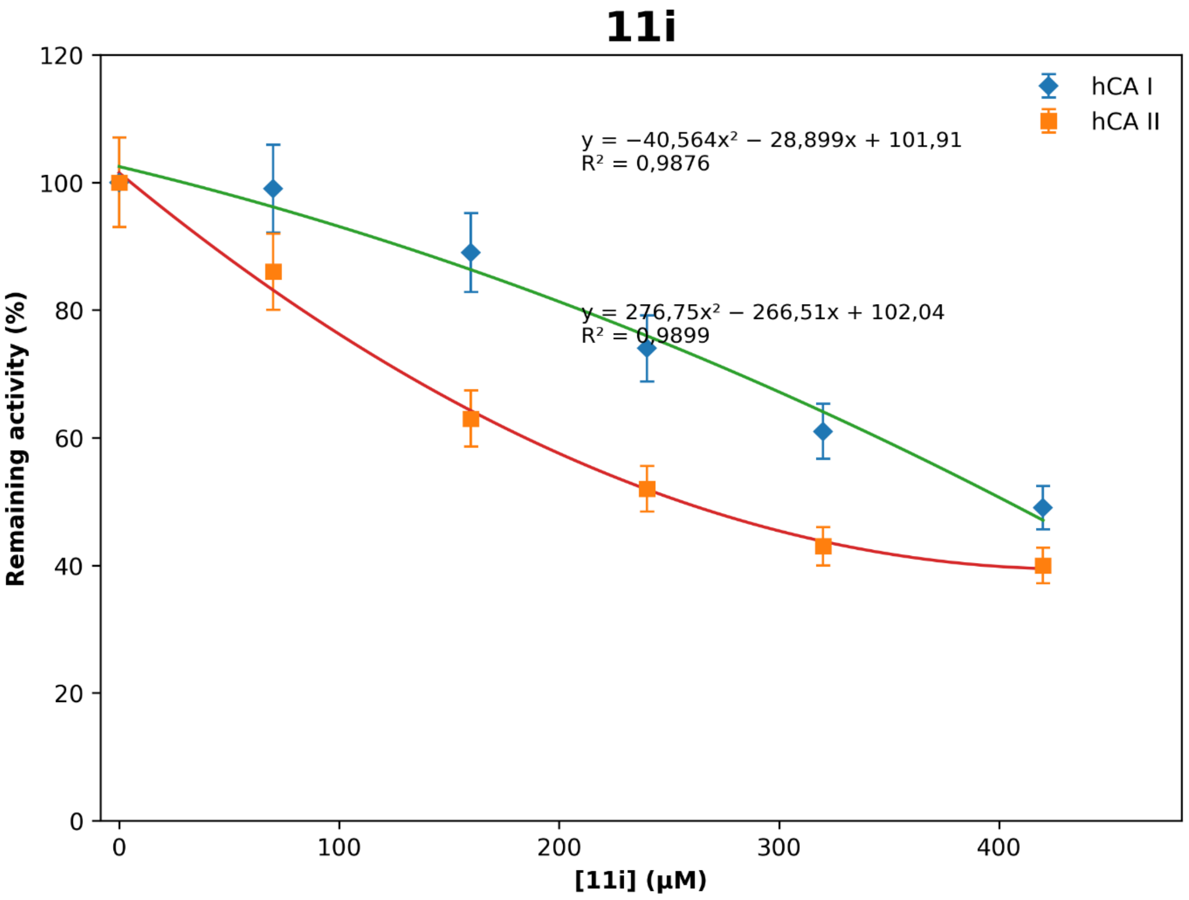

11i
hCA I
hCA II
y = −40,564x² − 28,899x + 101,91
R² = 0,9876
y = 276,75x² − 266,51x + 102,04
R² = 0,9899
Remaining activity (%)
[11i] (μM)

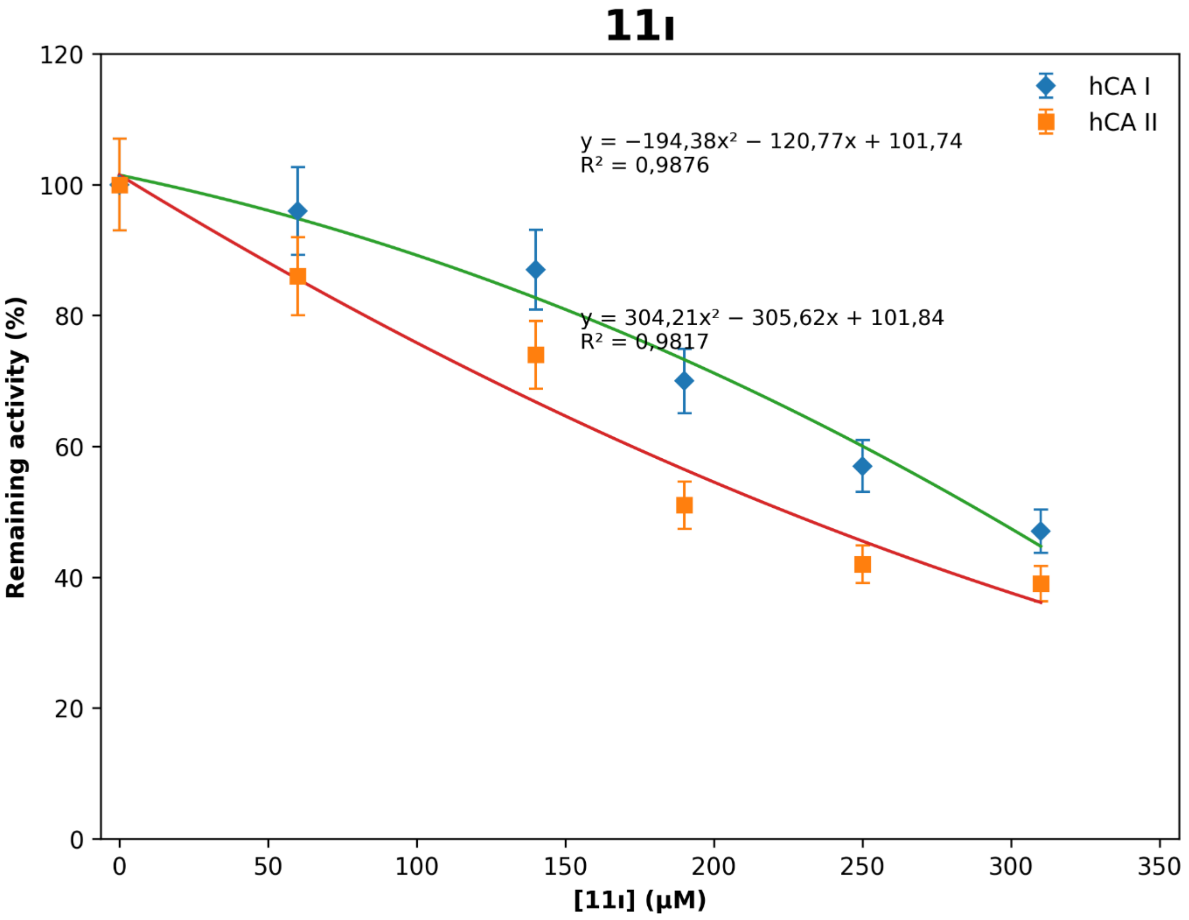

11ı
hCA I
hCA II
y = −194,38x² − 120,77x + 101,74
R² = 0,9876
y = 304,21x² − 305,62x + 101,84
R² = 0,9817
Remaining activity (%)
[11ı] (μM)

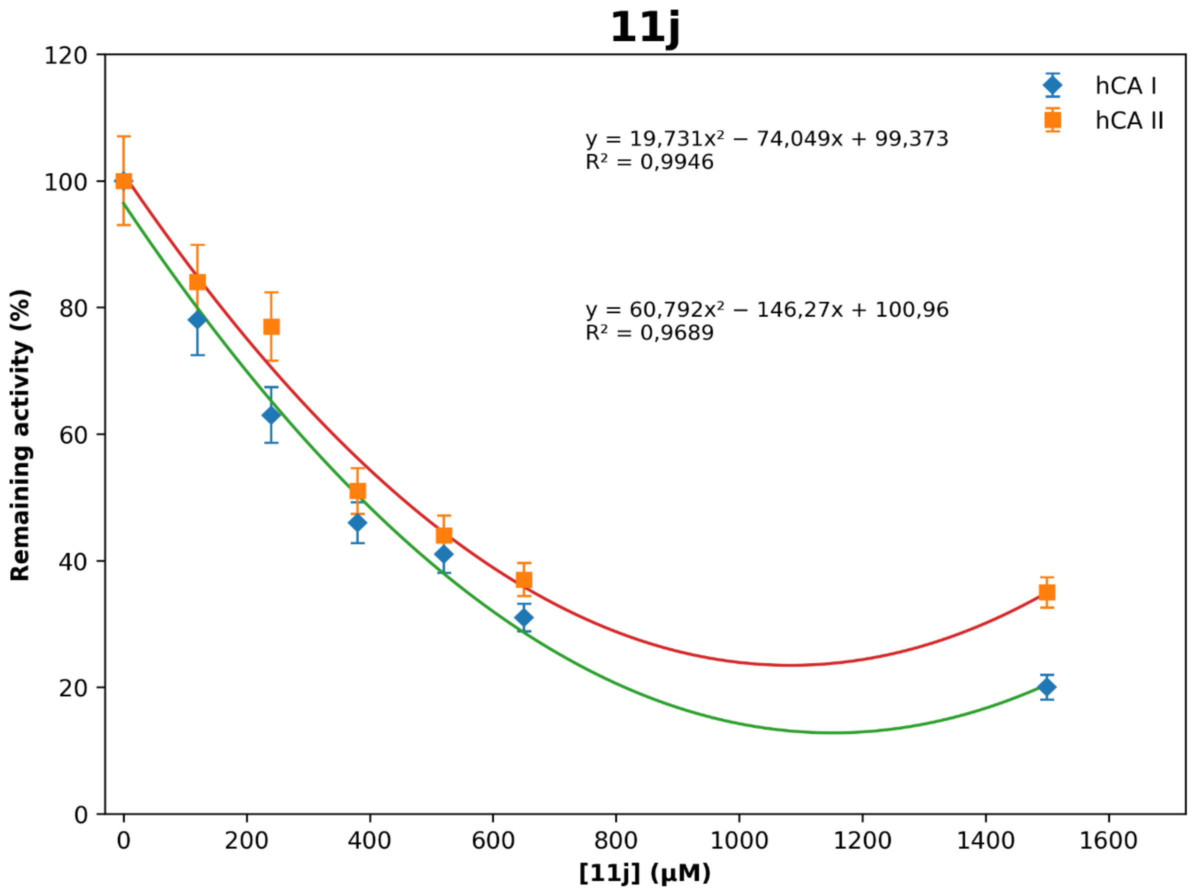
11j
hCA I
hCA II
y = 19,731x² − 74,049x + 99,373
R² = 0,9946
y = 60,792x² − 146,27x + 100,96
R² = 0,9689
Remaining activity (%)
[11j] (µM)

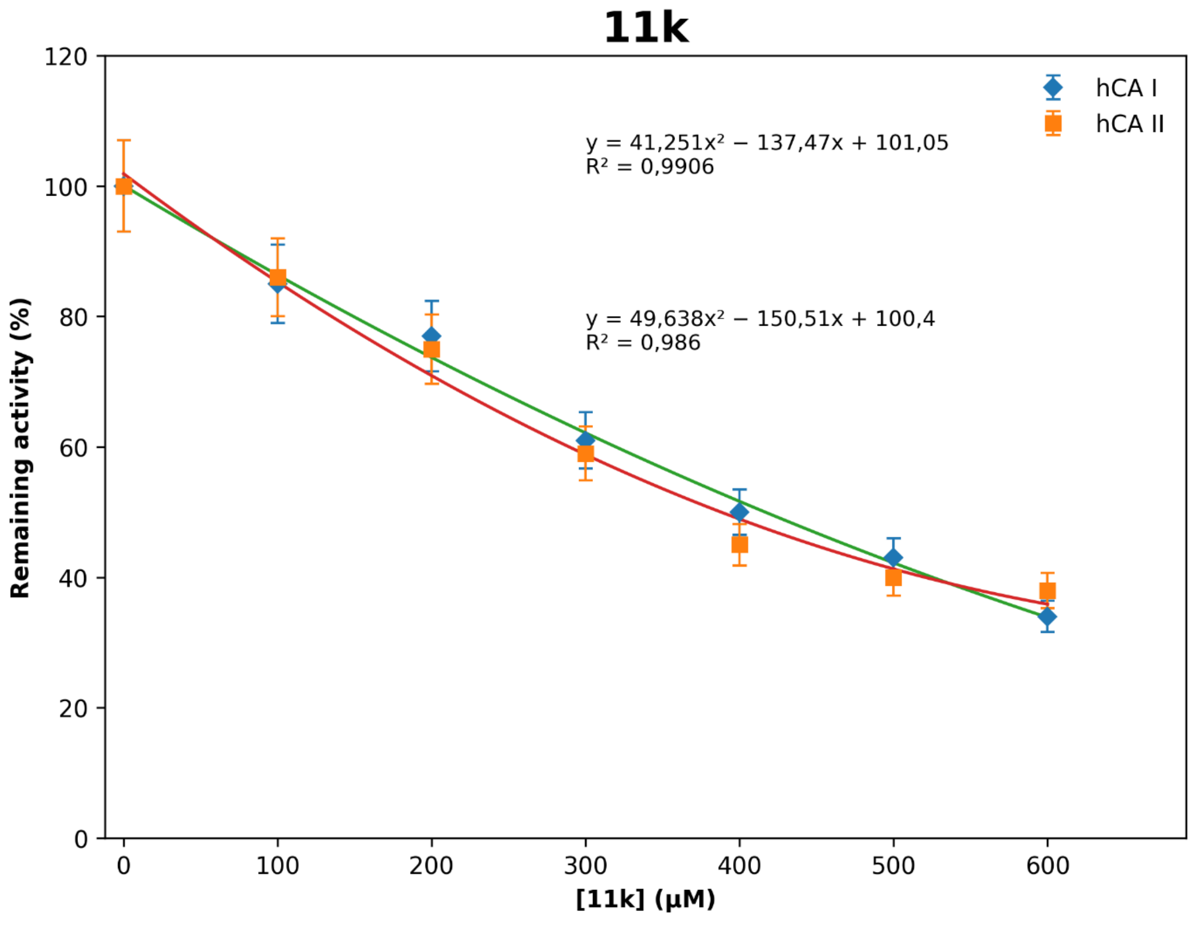
11k
hCA I
hCA II
y = 41,251x² − 137,47x + 101,05
R² = 0,9906
y = 49,638x² − 150,51x + 100,4
R² = 0,986
Remaining activity (%)
[11k] (µM)

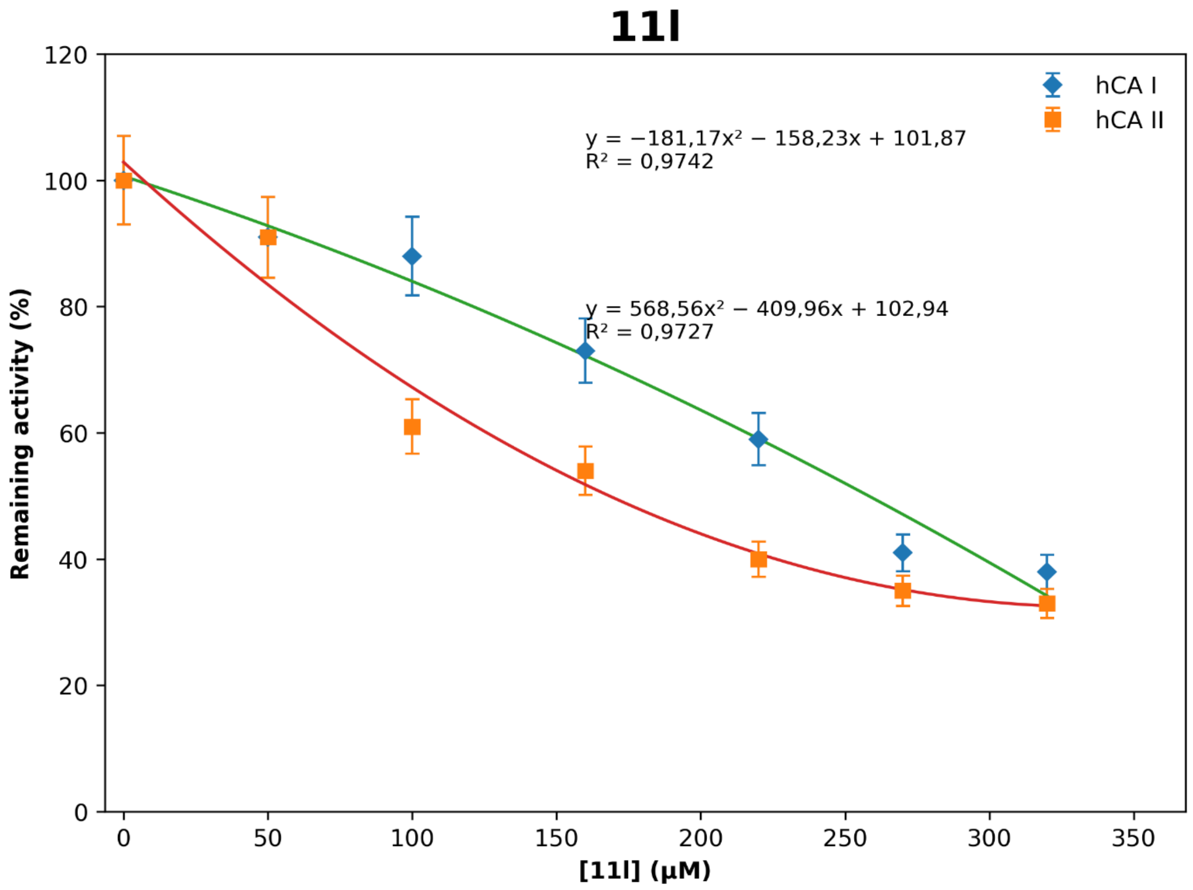
11l
hCA I
hCA II
y = −181,17x² − 158,23x + 101,87
R² = 0,9742
y = 568,56x² − 409,96x + 102,94
R² = 0,9727
Remaining activity (%)
[11l] (µM)

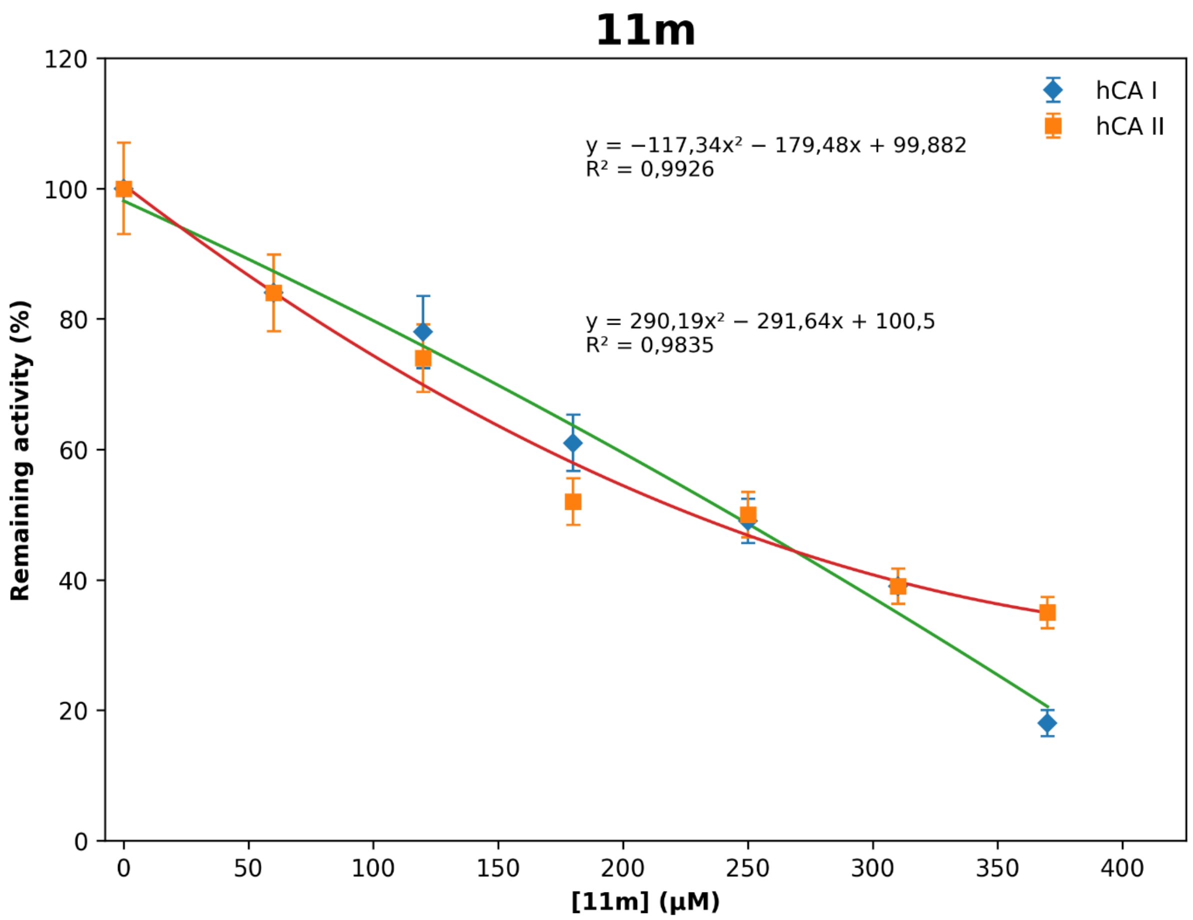
11m
hCA I
hCA II
y = −117,34x² − 179,48x + 99,882
R² = 0,9926
y = 290,19x² − 291,64x + 100,5
R² = 0,9835
Remaining activity (%)
[11m] (µM)

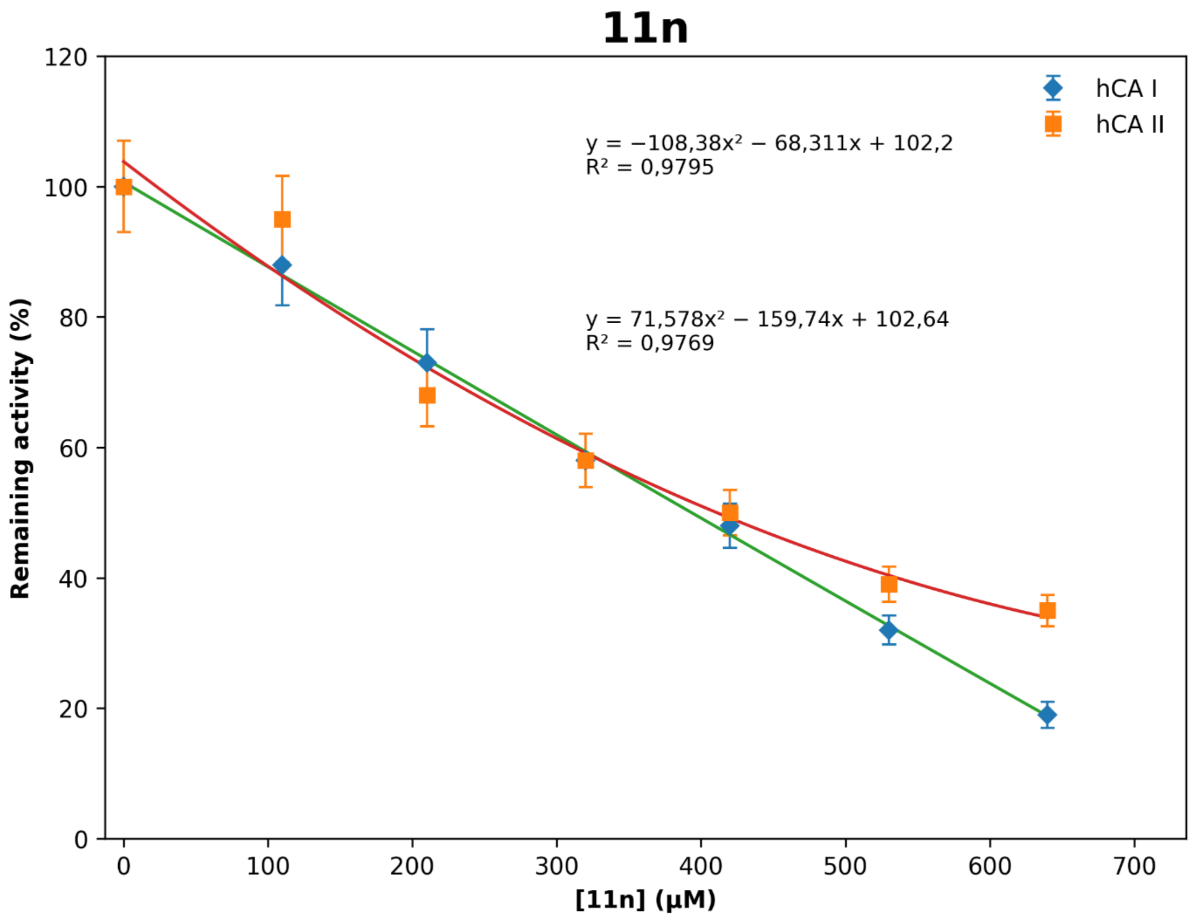

11n
hCA I
hCA II
y = −108,38x² − 68,311x + 102,2
R² = 0,9795
y = 71,578x² − 159,74x + 102,64
R² = 0,9769
Remaining activity (%)
[11n] (µM)
0
20
40
60
80
100
120
0
100
200
300
400
500
600
700

CA I-II K_i (ESTERASE)

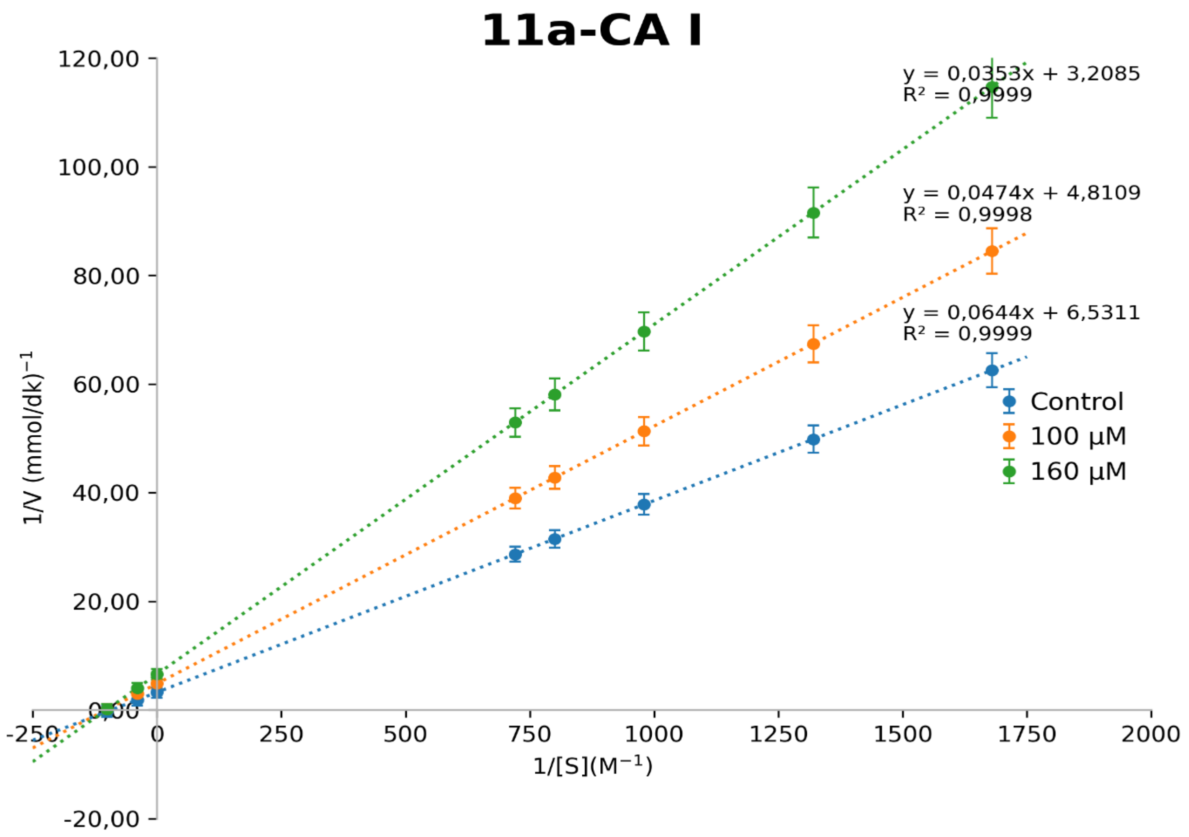

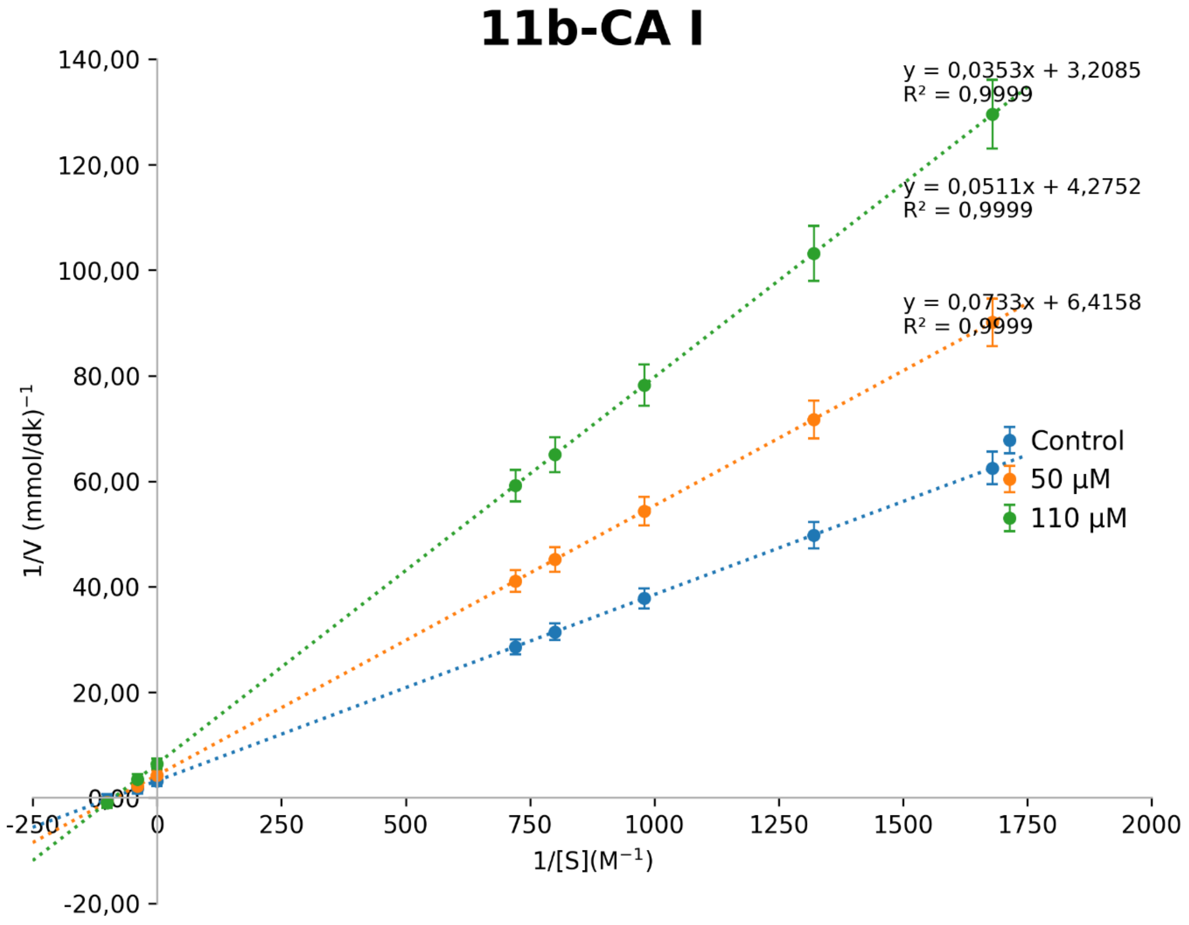

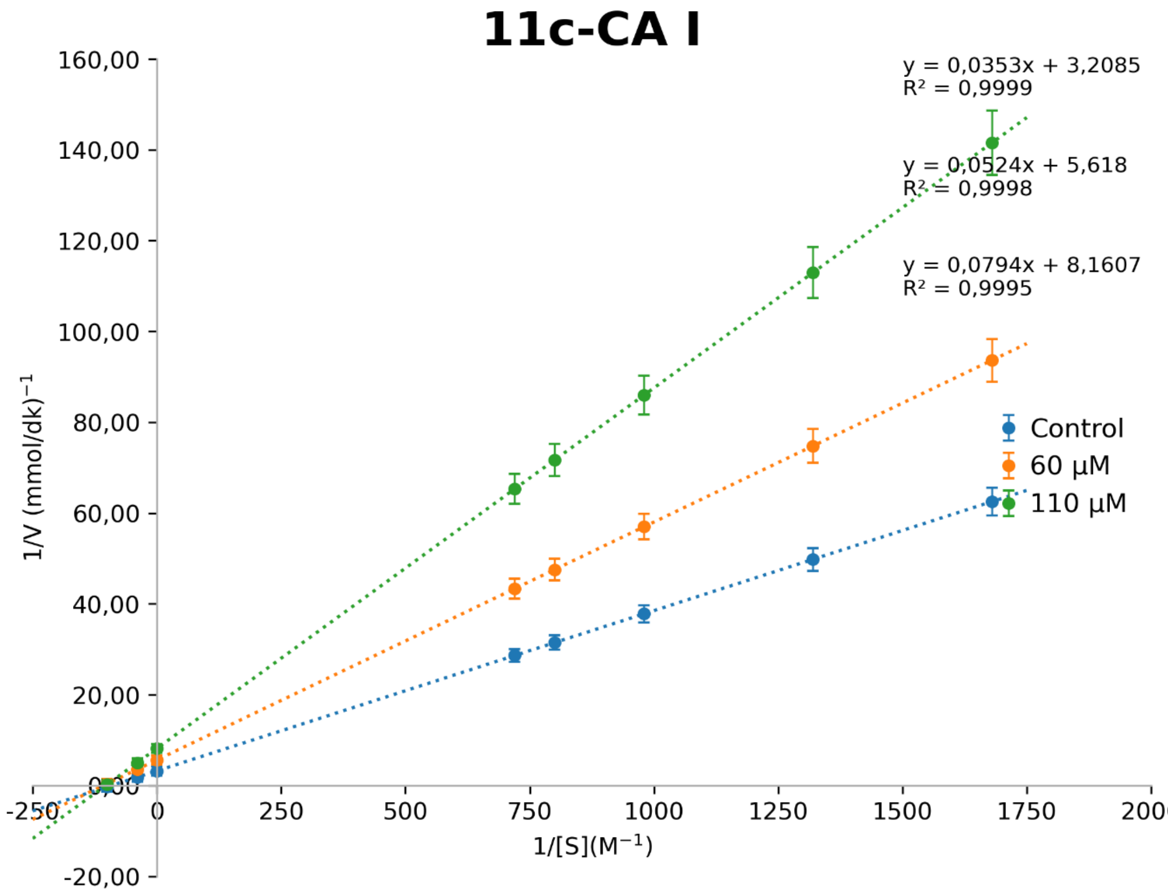

11c-CA I
y = 0,0353x + 3,2085
R² = 0,9999
y = 0,0524x + 5,618
R² = 0,9998
y = 0,0794x + 8,1607
R² = 0,9995
Control
60 µM
110 µM
1/V (mmol/dk)⁻¹
1/[S](M⁻¹)
160,00
140,00
120,00
100,00
80,00
60,00
40,00
20,00
0,00
-20,00
-250
0
250
500
750
1000
1250
1500
1750
2000

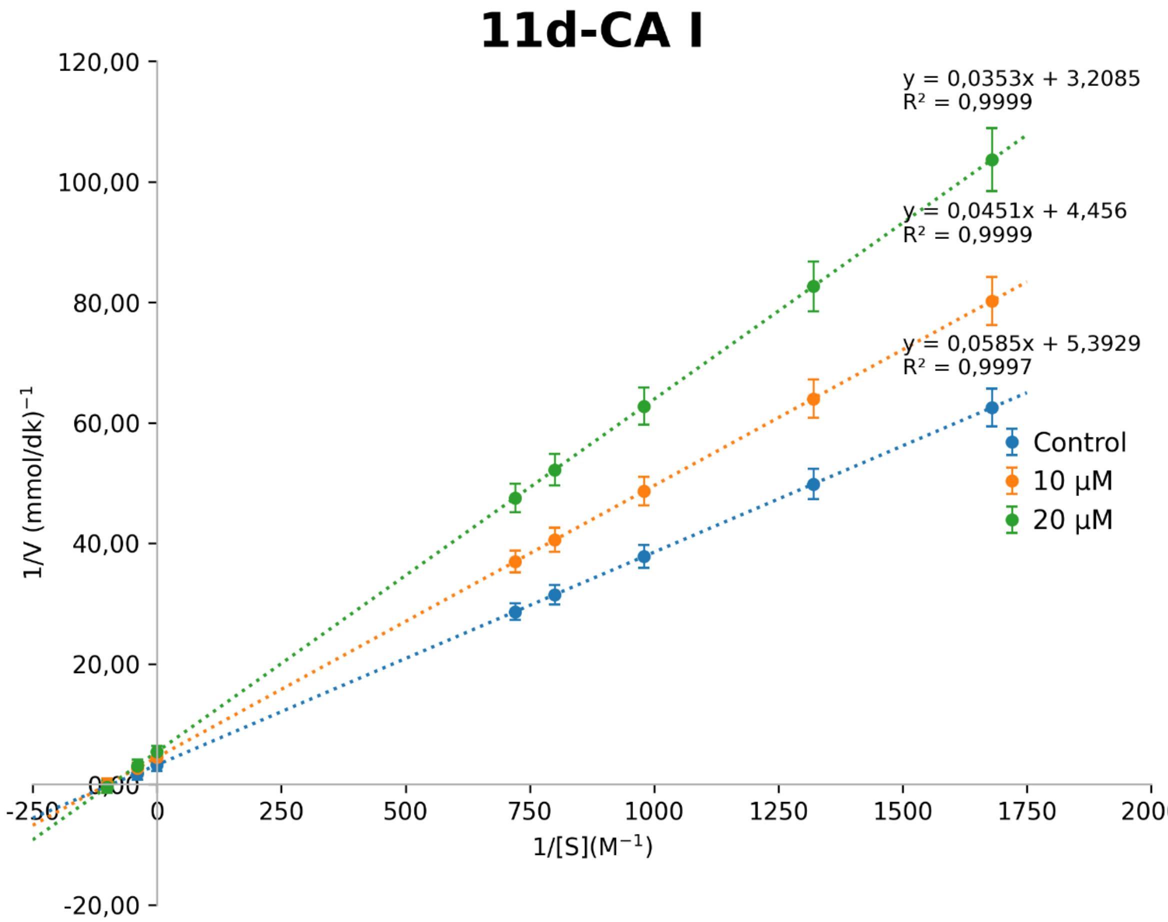

11d-CA I
y = 0,0353x + 3,2085
R² = 0,9999
y = 0,0451x + 4,456
R² = 0,9999
y = 0,0585x + 5,3929
R² = 0,9997
Control
10 µM
20 µM
1/V (mmol/dk)⁻¹
1/[S](M⁻¹)
120,00
100,00
80,00
60,00
40,00
20,00
0,00
-20,00
-250
0
250
500
750
1000
1250
1500
1750
2000

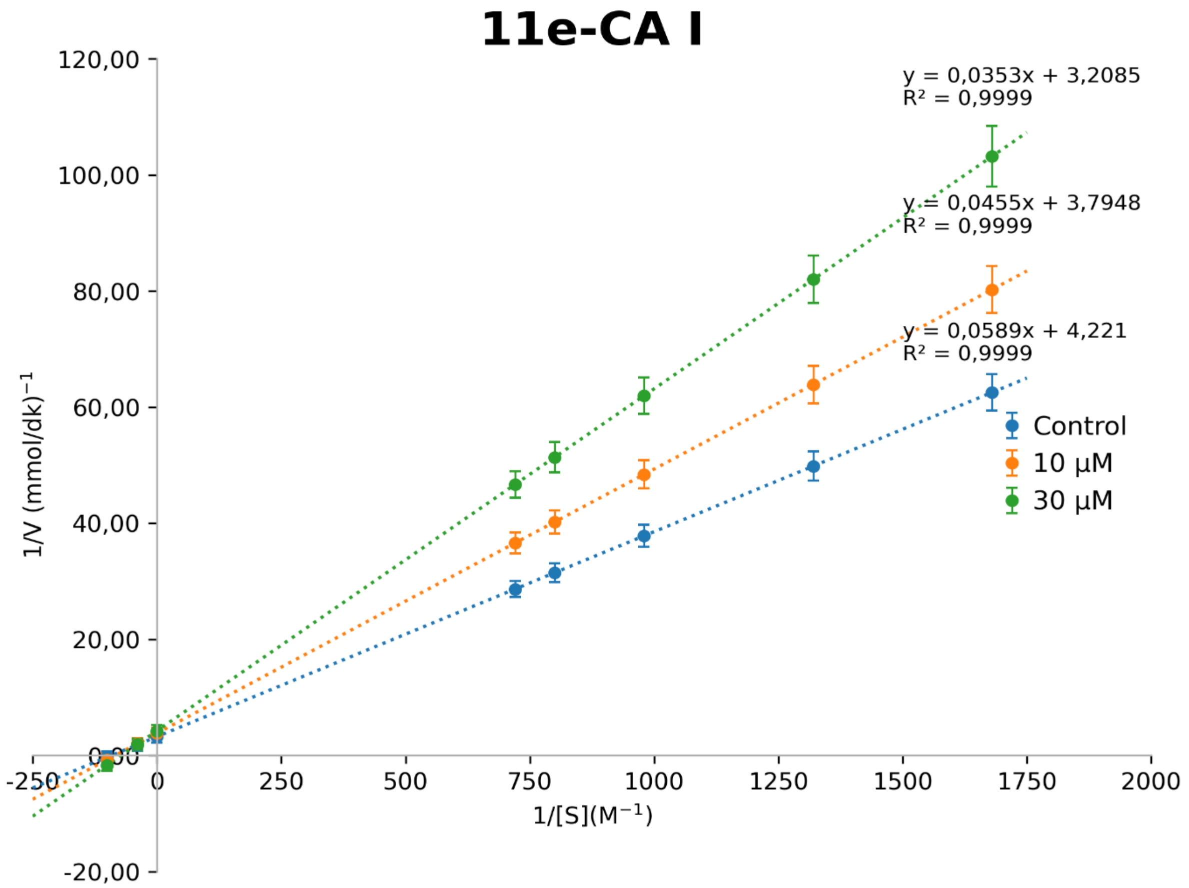
11e-CA I
y = 0,0353x + 3,2085
R² = 0,9999
y = 0,0455x + 3,7948
R² = 0,9999
y = 0,0589x + 4,221
R² = 0,9999
Control
10 µM
30 µM
1/V (mmol/dk)⁻¹
1/[S](M⁻¹)
120,00
100,00
80,00
60,00
40,00
20,00
0,00
-20,00
-250
0
250
500
750
1000
1250
1500
1750
2000

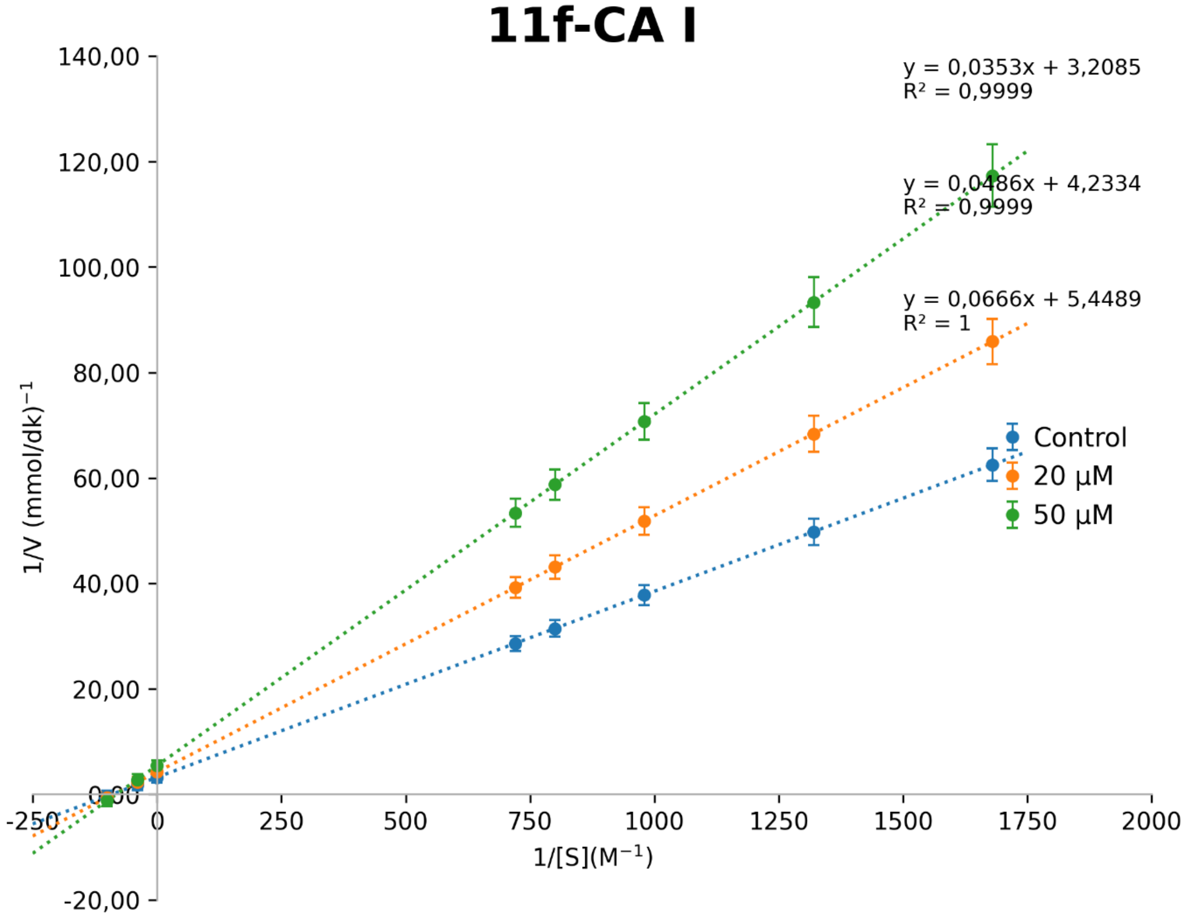
11f-CA I
y = 0,0353x + 3,2085
R² = 0,9999
y = 0,0486x + 4,2334
R² = 0,9999
y = 0,0666x + 5,4489
R² = 1
Control
20 µM
50 µM
1/V (mmol/dk)⁻¹
1/[S](M⁻¹)
140,00
120,00
100,00
80,00
60,00
40,00
20,00
0,00
-20,00
-250
0
250
500
750
1000
1250
1500
1750
2000

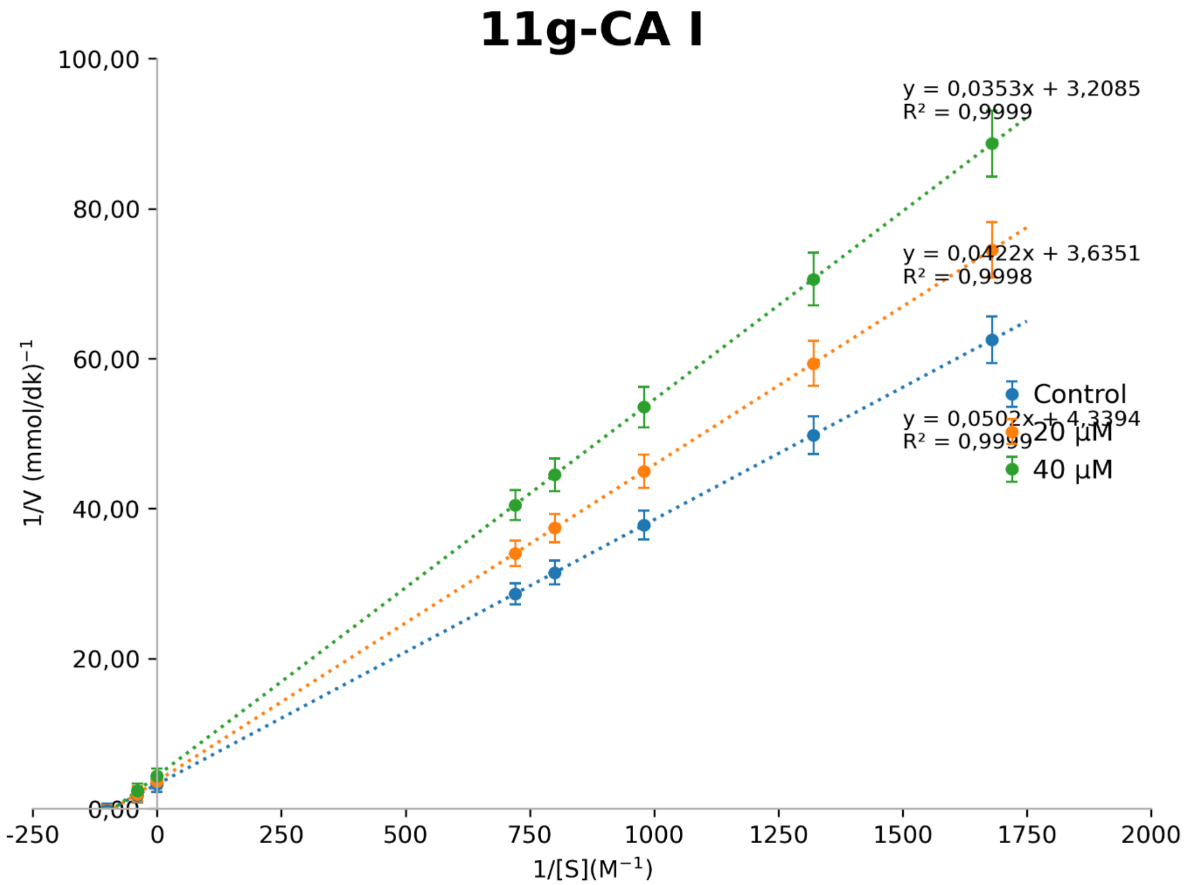
11g-CA I
y = 0,0353x + 3,2085
R² = 0,9999
y = 0,0422x + 3,6351
R² = 0,9998
y = 0,0502x + 4,3394
R² = 0,9999
Control
20 µM
40 µM
1/V (mmol/dk)⁻¹
1/[S](M⁻¹)
100,00
80,00
60,00
40,00
20,00
0,00
-250
0
250
500
750
1000
1250
1500
1750
2000

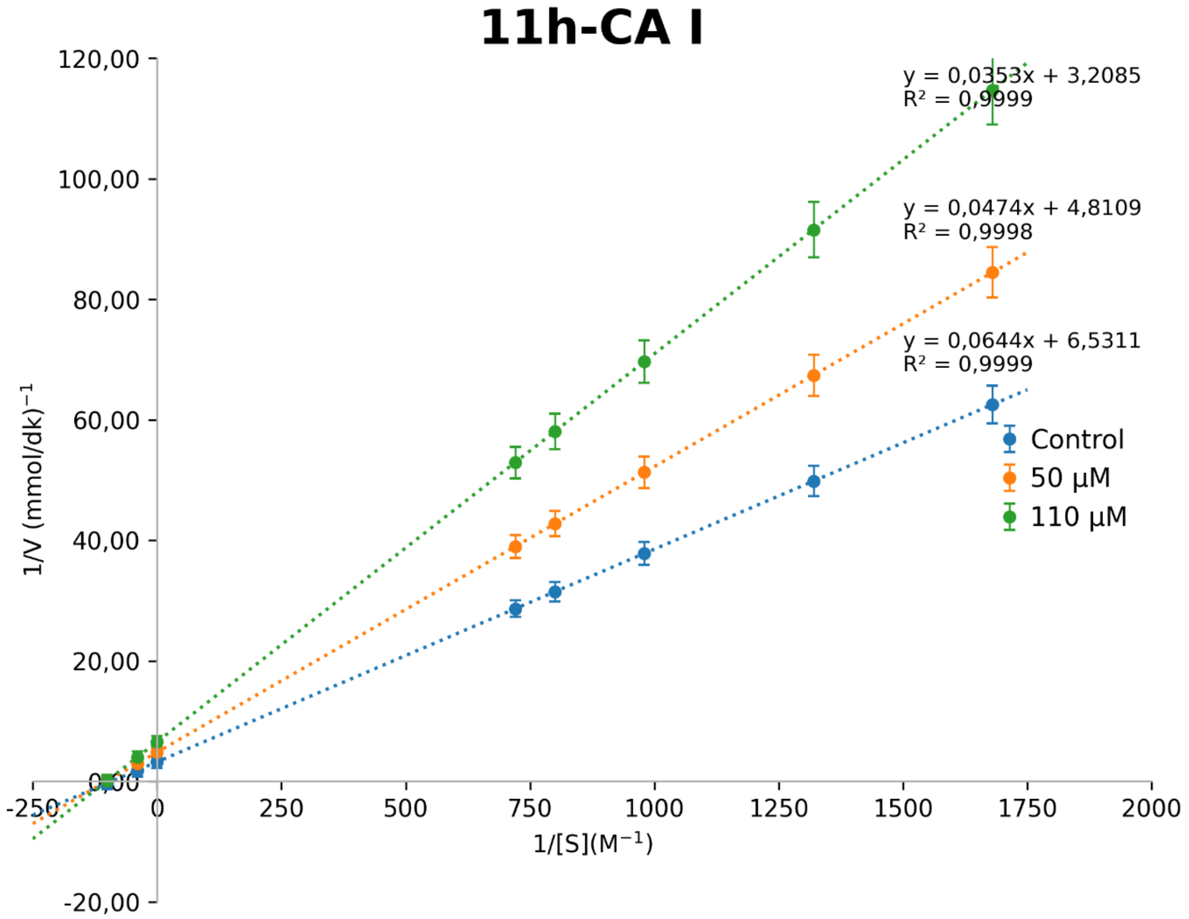
11h-CA I
y = 0,0353x + 3,2085
R² = 0,9999
y = 0,0474x + 4,8109
R² = 0,9998
y = 0,0644x + 6,5311
R² = 0,9999
Control
50 µM
110 µM
1/V (mmol/dk)⁻¹
1/[S](M⁻¹)
120,00
100,00
80,00
60,00
40,00
20,00
0,00
-20,00
-250
0
250
500
750
1000
1250
1500
1750
2000

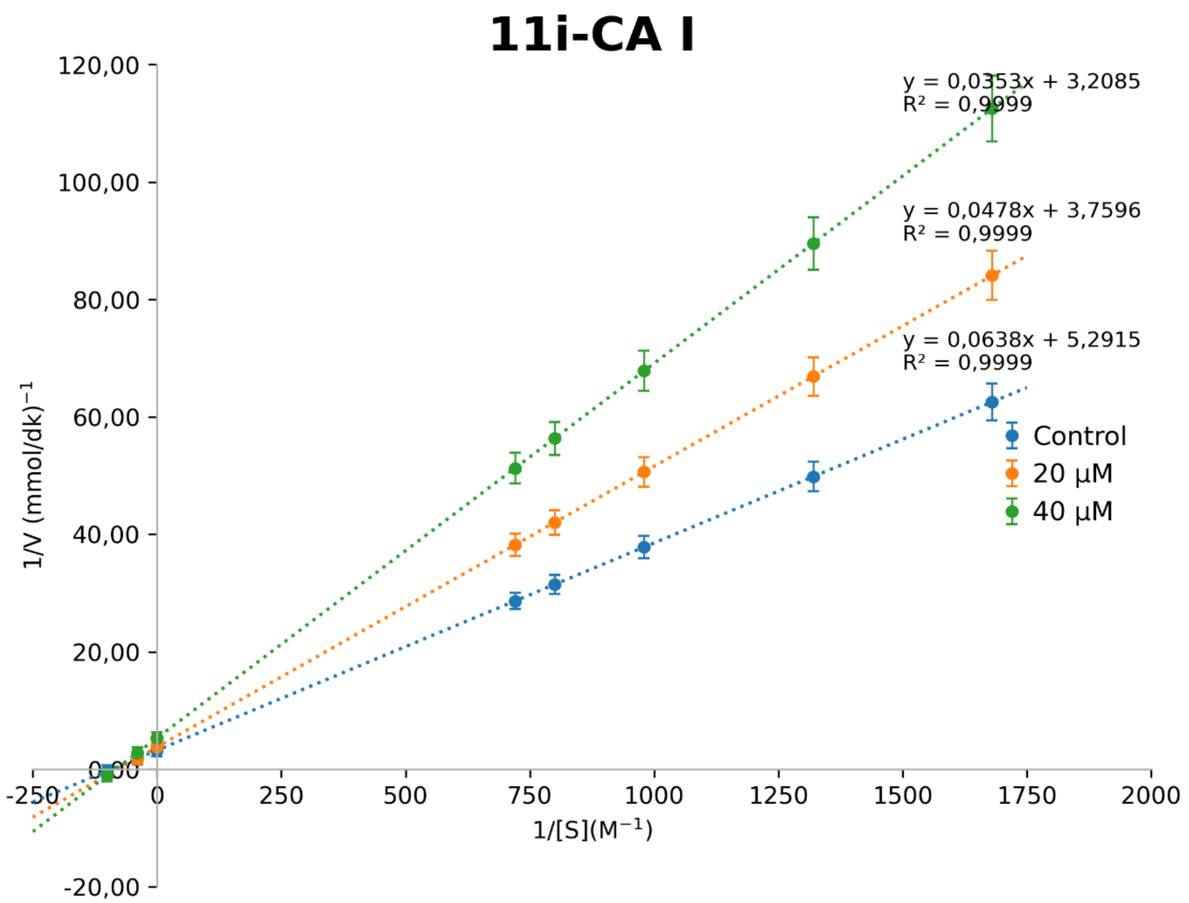
11i-CA I
y = 0,0353x + 3,2085
R² = 0,9999
y = 0,0478x + 3,7596
R² = 0,9999
y = 0,0638x + 5,2915
R² = 0,9999
Control
20 µM
40 µM
1/V (mmol/dk)⁻¹
1/[S](M⁻¹)
120,00
100,00
80,00
60,00
40,00
20,00
0,00
-20,00
-250
0
250
500
750
1000
1250
1500
1750
2000

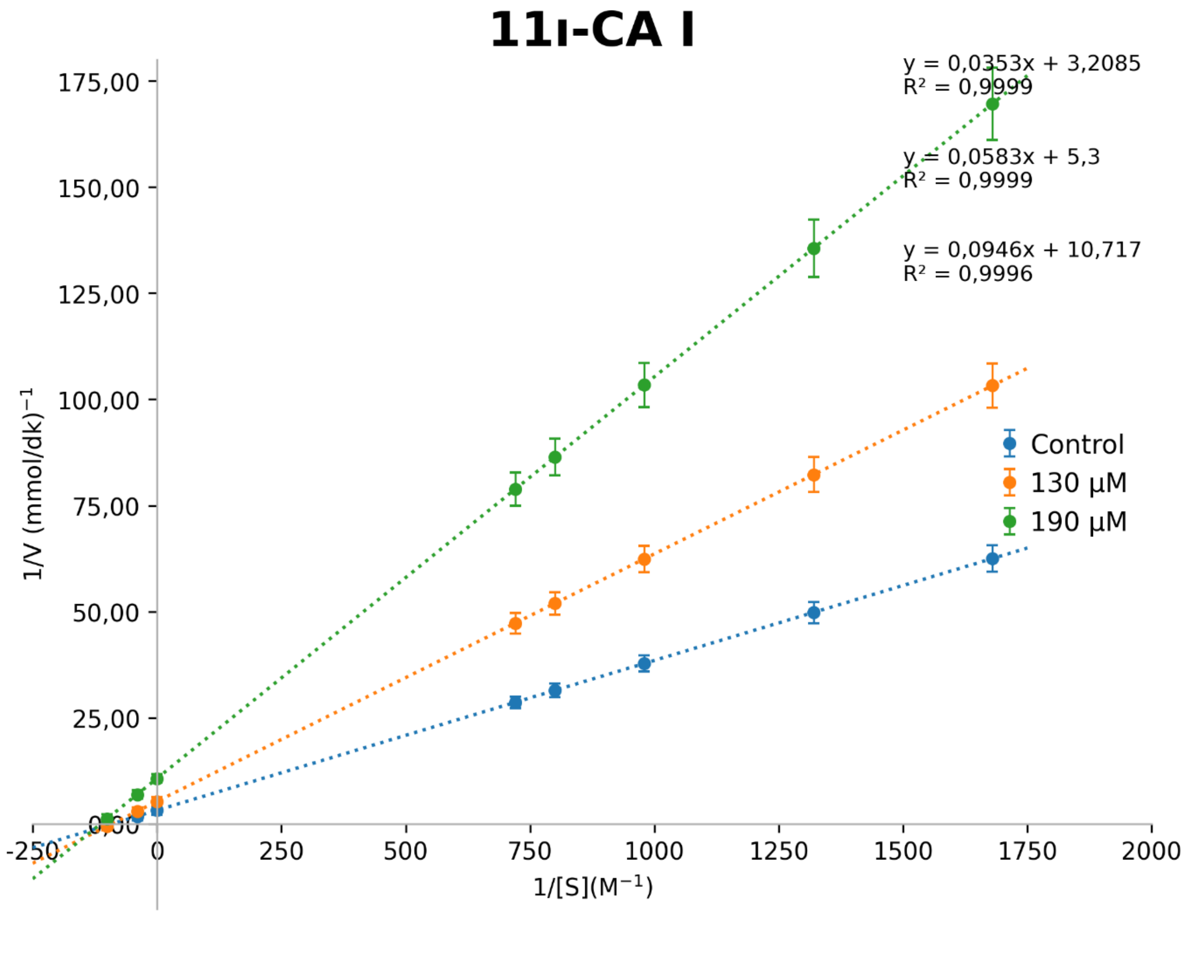
11ı-CA I
y = 0,0353x + 3,2085
R² = 0,9999
y = 0,0583x + 5,3
R² = 0,9999
y = 0,0946x + 10,717
R² = 0,9996
Control
130 µM
190 µM
1/V (mmol/dk)⁻¹
1/[S](M⁻¹)
175,00
150,00
125,00
100,00
75,00
50,00
25,00
0,00
-250
0
250
500
750
1000
1250
1500
1750
2000

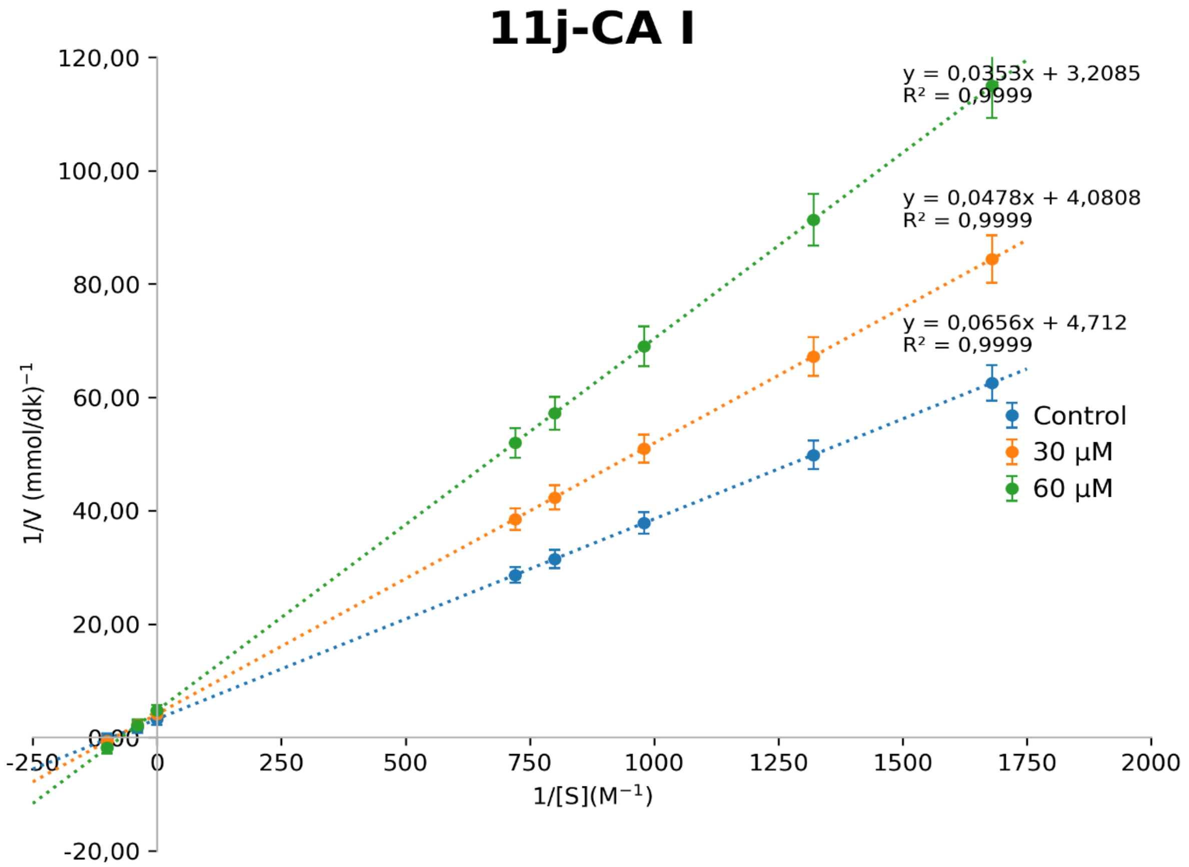
11j-CA I
y = 0,0353x + 3,2085
R² = 0,9999
y = 0,0478x + 4,0808
R² = 0,9999
y = 0,0656x + 4,712
R² = 0,9999
Control
30 µM
60 µM
1/V (mmol/dk)⁻¹
1/[S](M⁻¹)
120,00
100,00
80,00
60,00
40,00
20,00
0,00
-20,00
-250
0
250
500
750
1000
1250
1500
1750
2000

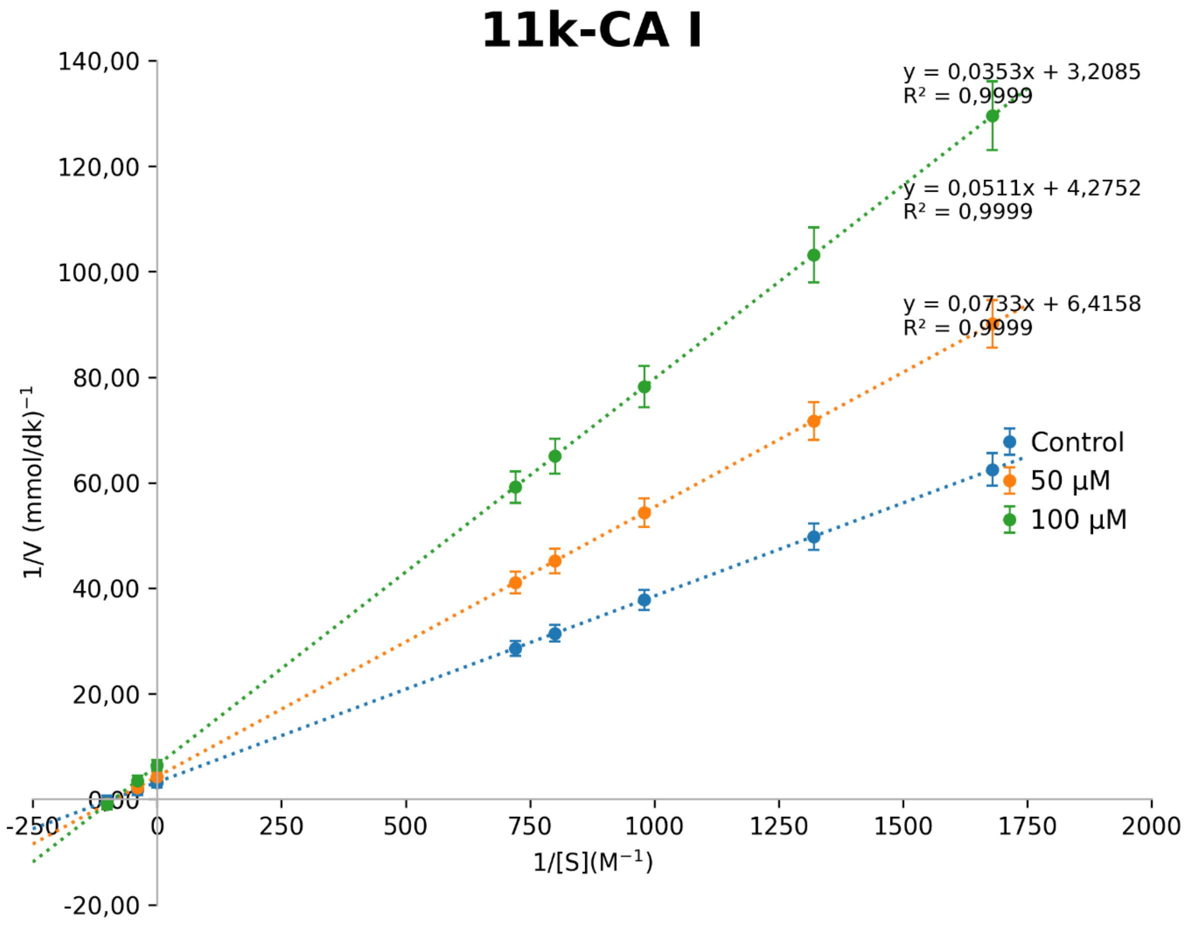
11k-CA I
y = 0,0353x + 3,2085
R² = 0,9999
y = 0,0511x + 4,2752
R² = 0,9999
y = 0,0733x + 6,4158
R² = 0,9999
Control
50 µM
100 µM
1/V (mmol/dk)⁻¹
1/[S](M⁻¹)
140,00
120,00
100,00
80,00
60,00
40,00
20,00
0,00
-20,00
-250
0
250
500
750
1000
1250
1500
1750
2000

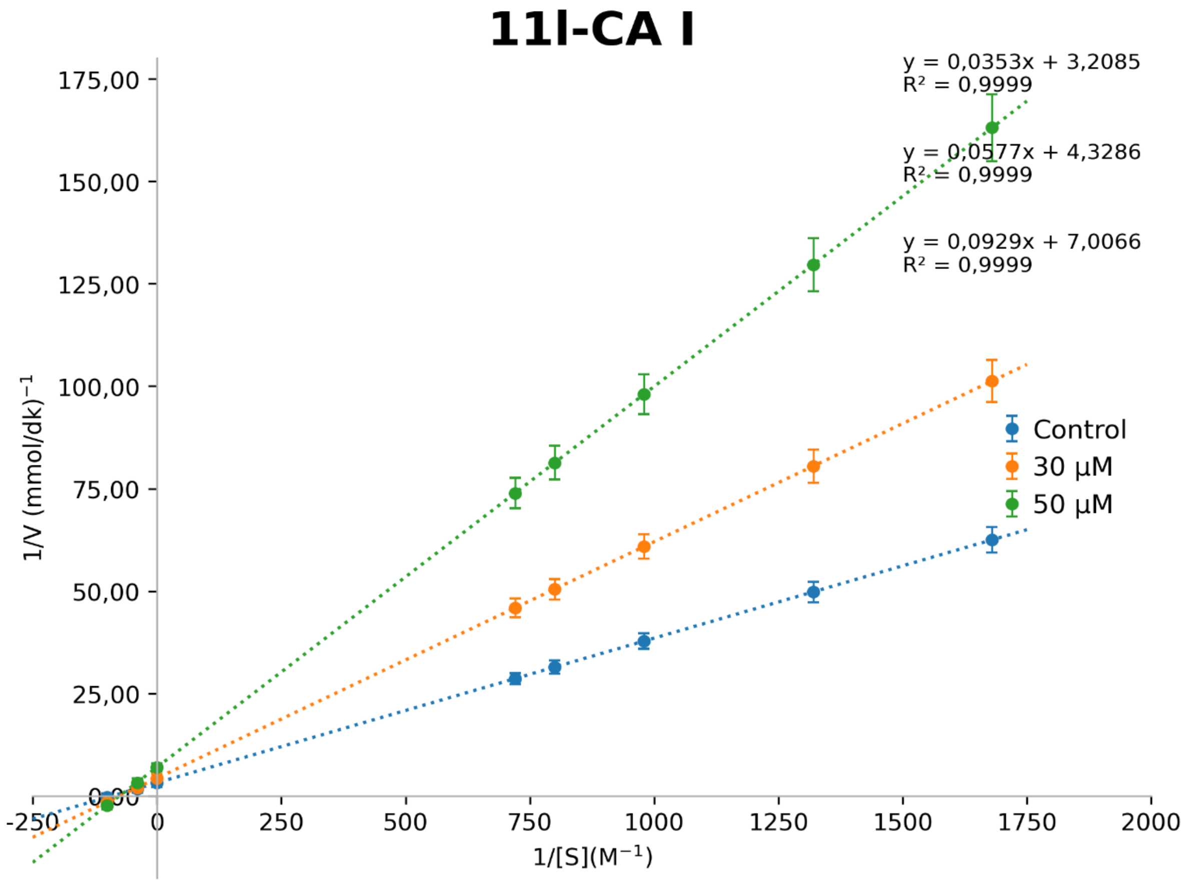
11l-CA I
y = 0,0353x + 3,2085
R² = 0,9999
y = 0,0577x + 4,3286
R² = 0,9999
y = 0,0929x + 7,0066
R² = 0,9999
Control
30 µM
50 µM
1/V (mmol/dk)⁻¹
1/[S](M⁻¹)
175,00
150,00
125,00
100,00
75,00
50,00
25,00
0,00
-250
0
250
500
750
1000
1250
1500
1750
2000

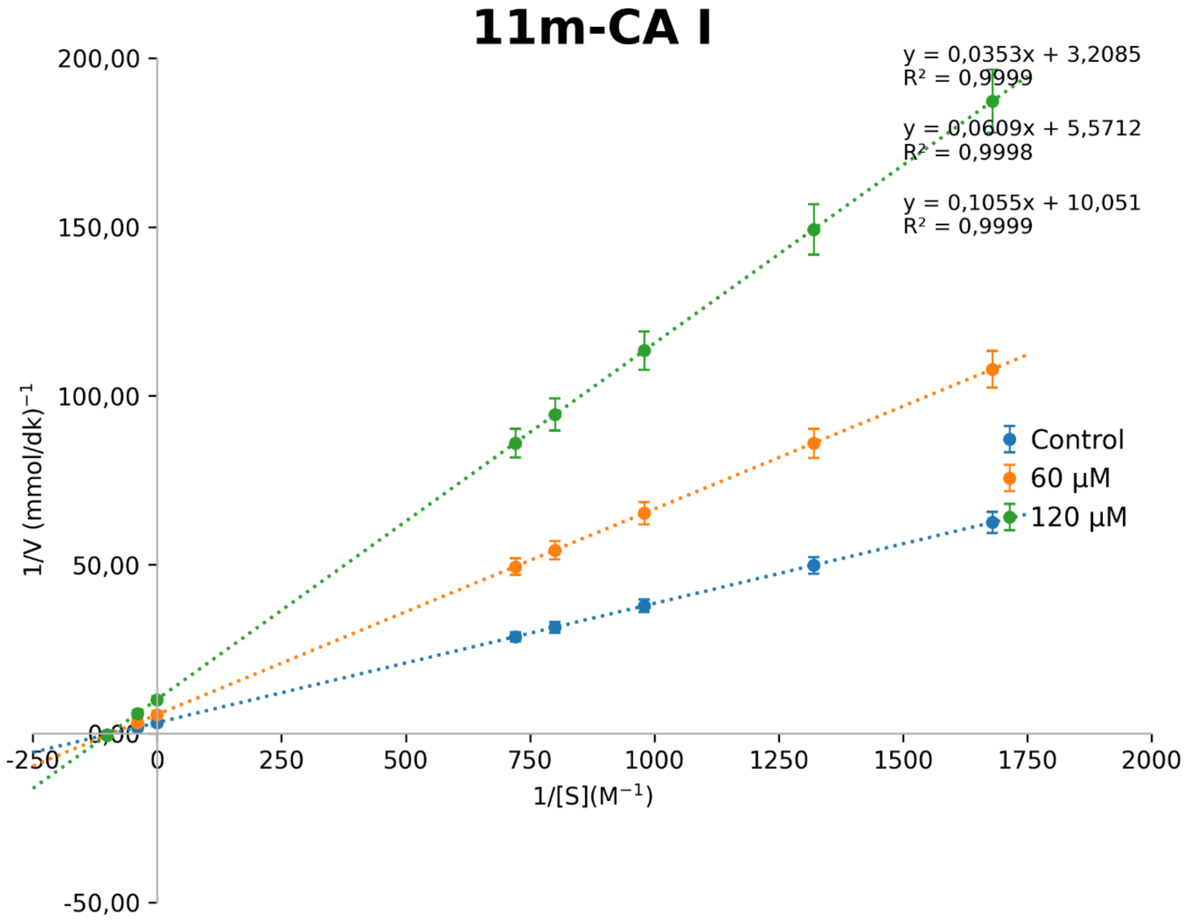
11m-CA I
y = 0,0353x + 3,2085
R² = 0,9999
y = 0,0609x + 5,5712
R² = 0,9998
y = 0,1055x + 10,051
R² = 0,9999
Control
60 µM
120 µM
1/V (mmol/dk)⁻¹
1/[S](M⁻¹)
200,00
150,00
100,00
50,00
0,00
-50,00
-250
0
250
500
750
1000
1250
1500
1750
2000

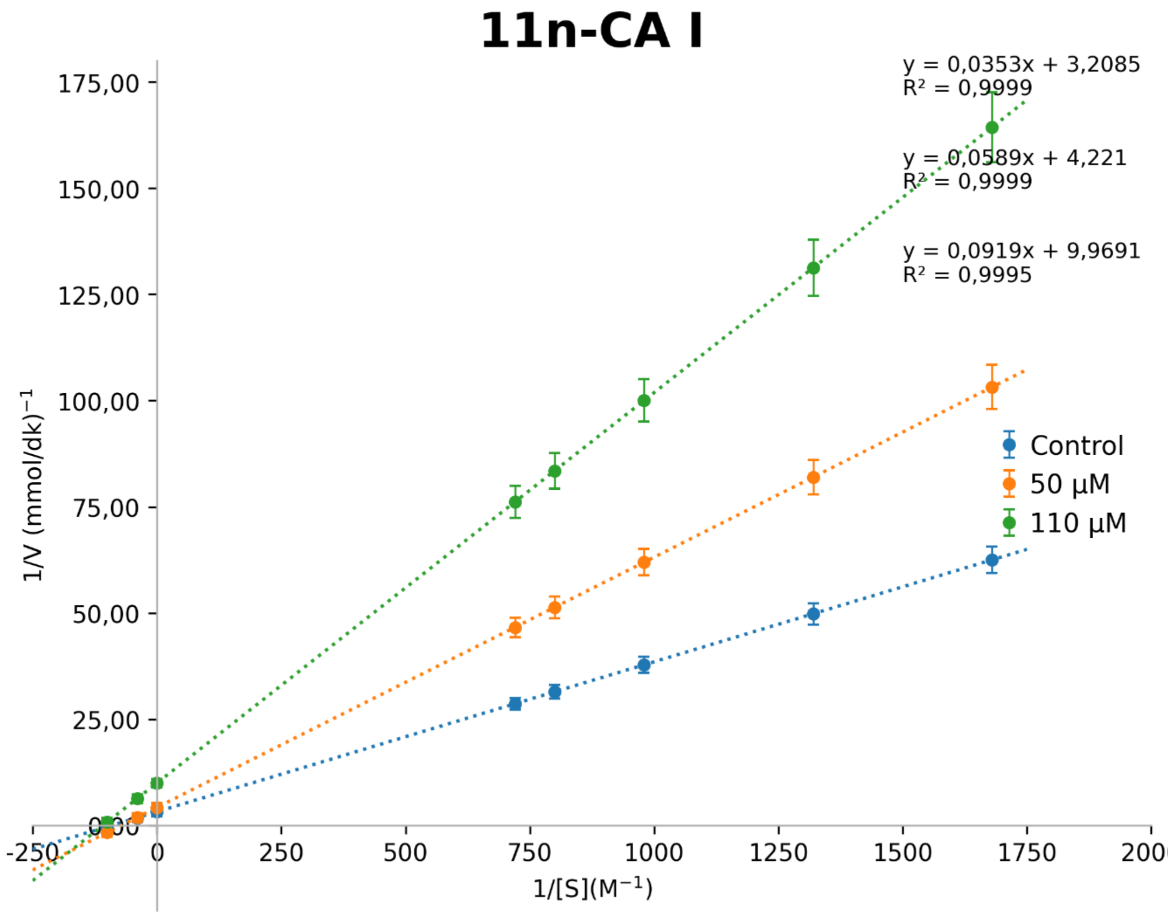

11n-CA I
y = 0,0353x + 3,2085
R² = 0,9999
y = 0,0589x + 4,221
R² = 0,9999
y = 0,0919x + 9,9691
R² = 0,9995
175,00
150,00
125,00
100,00
75,00
50,00
25,00
0,00
1/V (mmol/dk)⁻¹
-250
0
250
500
750
1000
1250
1500
1750
2000
1/[S](M⁻¹)
Control
50 µM
110 µM

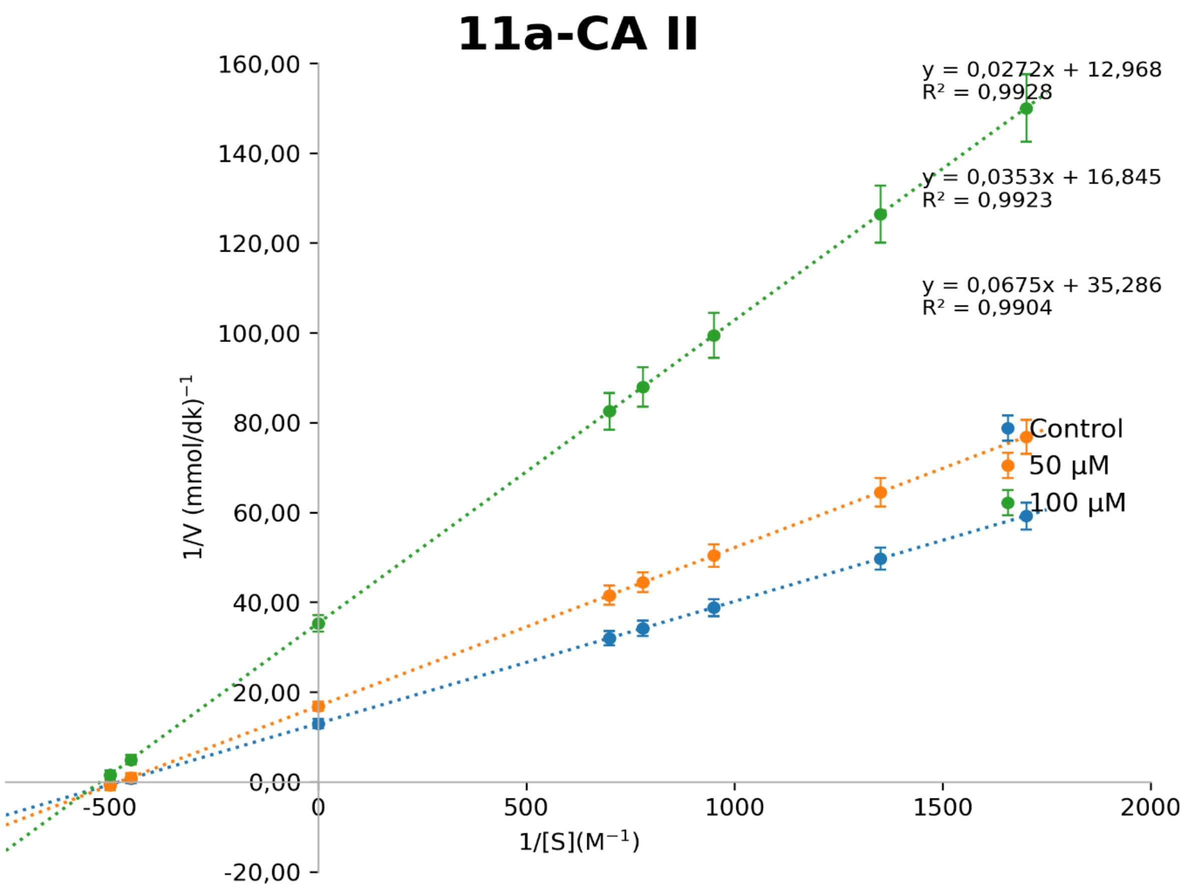
11a-CA II
y = 0,0272x + 12,968
R² = 0,9928
y = 0,0353x + 16,845
R² = 0,9923
y = 0,0675x + 35,286
R² = 0,9904
Control
50 µM
100 µM
1/V (mmol/dk)$^{-1}$
1/[S](M^{-1})
160,00
140,00
120,00
100,00
80,00
60,00
40,00
20,00
0,00
-20,00
-500
0
500
1000
1500
2000

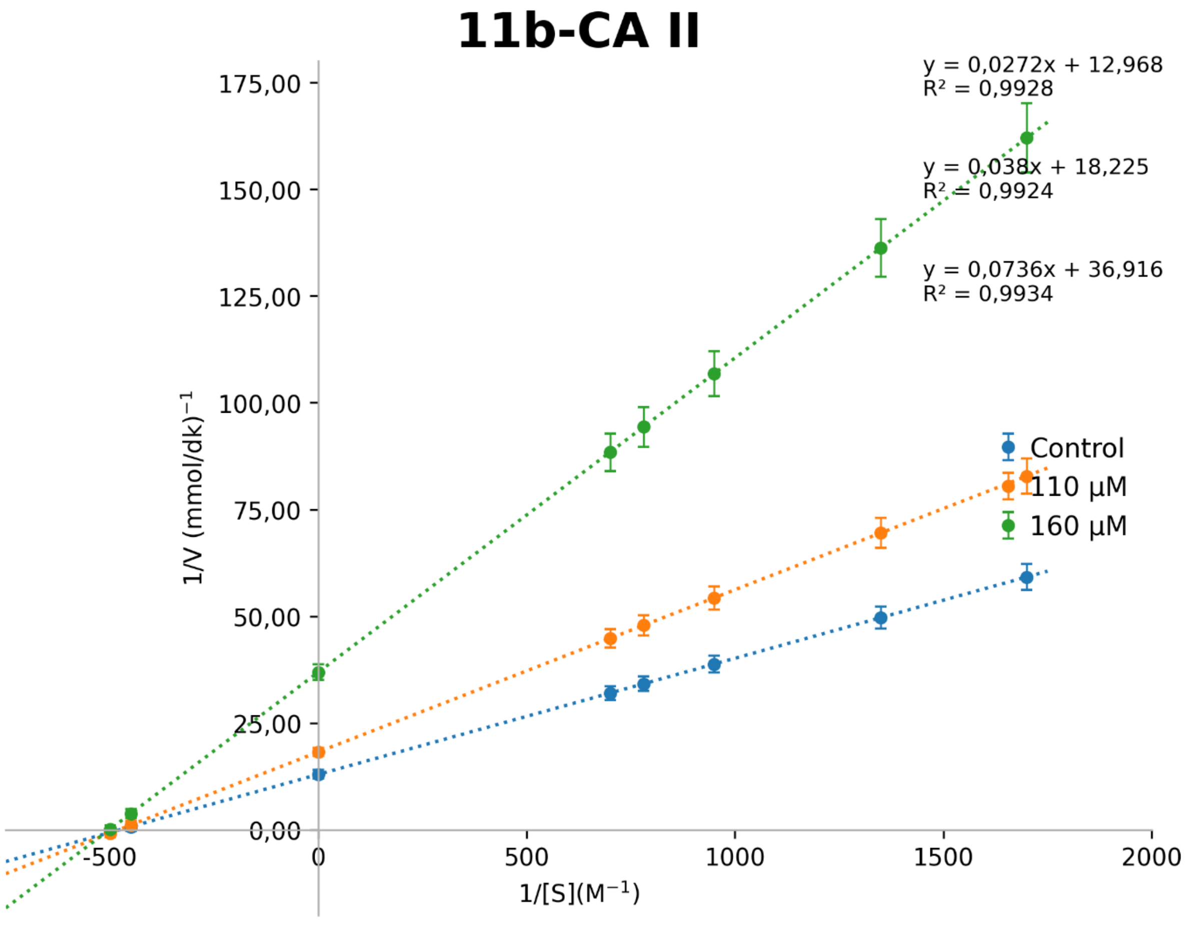
11b-CA II
y = 0,0272x + 12,968
R² = 0,9928
y = 0,038x + 18,225
R² = 0,9924
y = 0,0736x + 36,916
R² = 0,9934
Control
110 µM
160 µM
1/V (mmol/dk)$^{-1}$
1/[S](M^{-1})
175,00
150,00
125,00
100,00
75,00
50,00
25,00
0,00
-500
0
500
1000
1500
2000

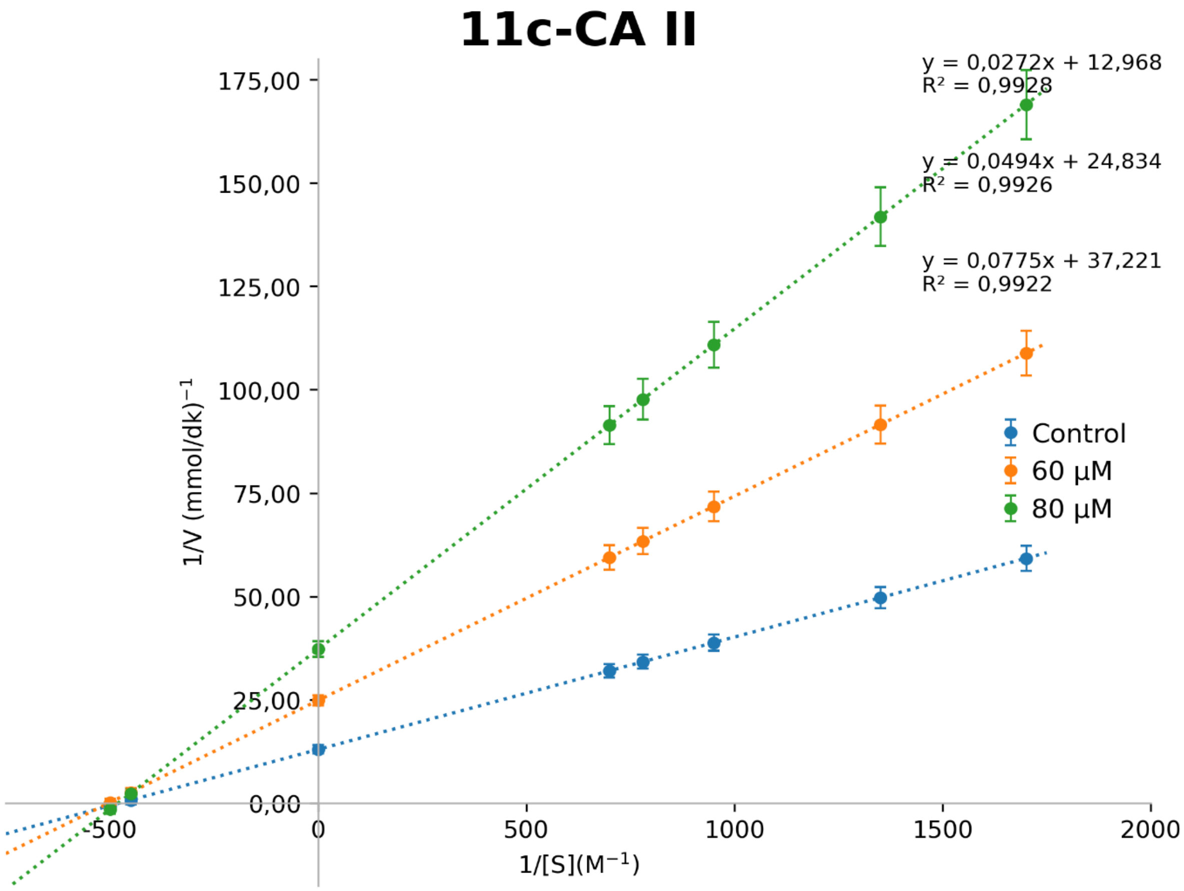
11c-CA II
y = 0,0272x + 12,968
R² = 0,9928
y = 0,0494x + 24,834
R² = 0,9926
y = 0,0775x + 37,221
R² = 0,9922
Control
60 µM
80 µM
1/V (mmol/dk)⁻¹
1/[S](M⁻¹)
175,00
150,00
125,00
100,00
75,00
50,00
25,00
0,00
-500
0
500
1000
1500
2000

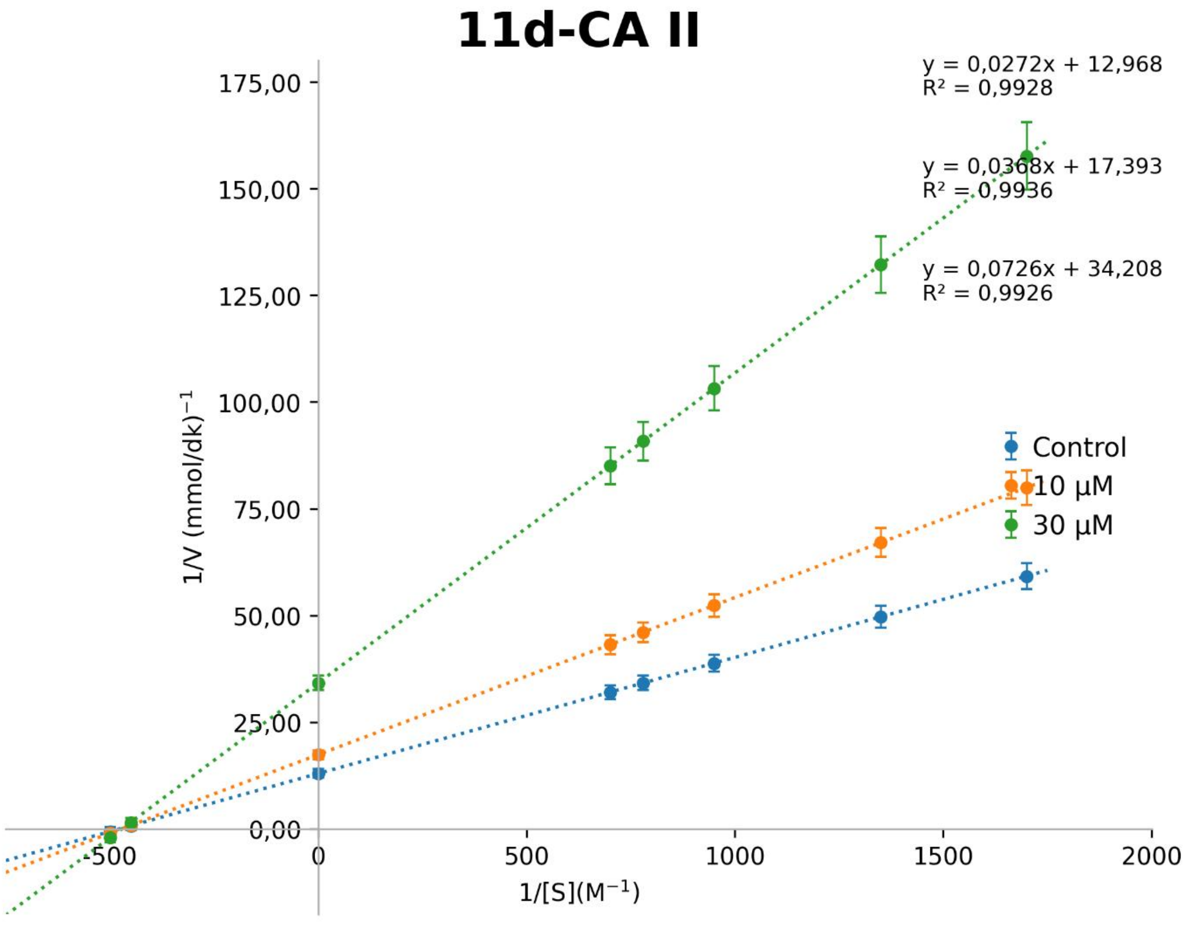
11d-CA II
y = 0,0272x + 12,968
R² = 0,9928
y = 0,0368x + 17,393
R² = 0,9936
y = 0,0726x + 34,208
R² = 0,9926
Control
10 µM
30 µM
1/V (mmol/dk)⁻¹
1/[S](M⁻¹)
175,00
150,00
125,00
100,00
75,00
50,00
25,00
0,00
-500
0
500
1000
1500
2000

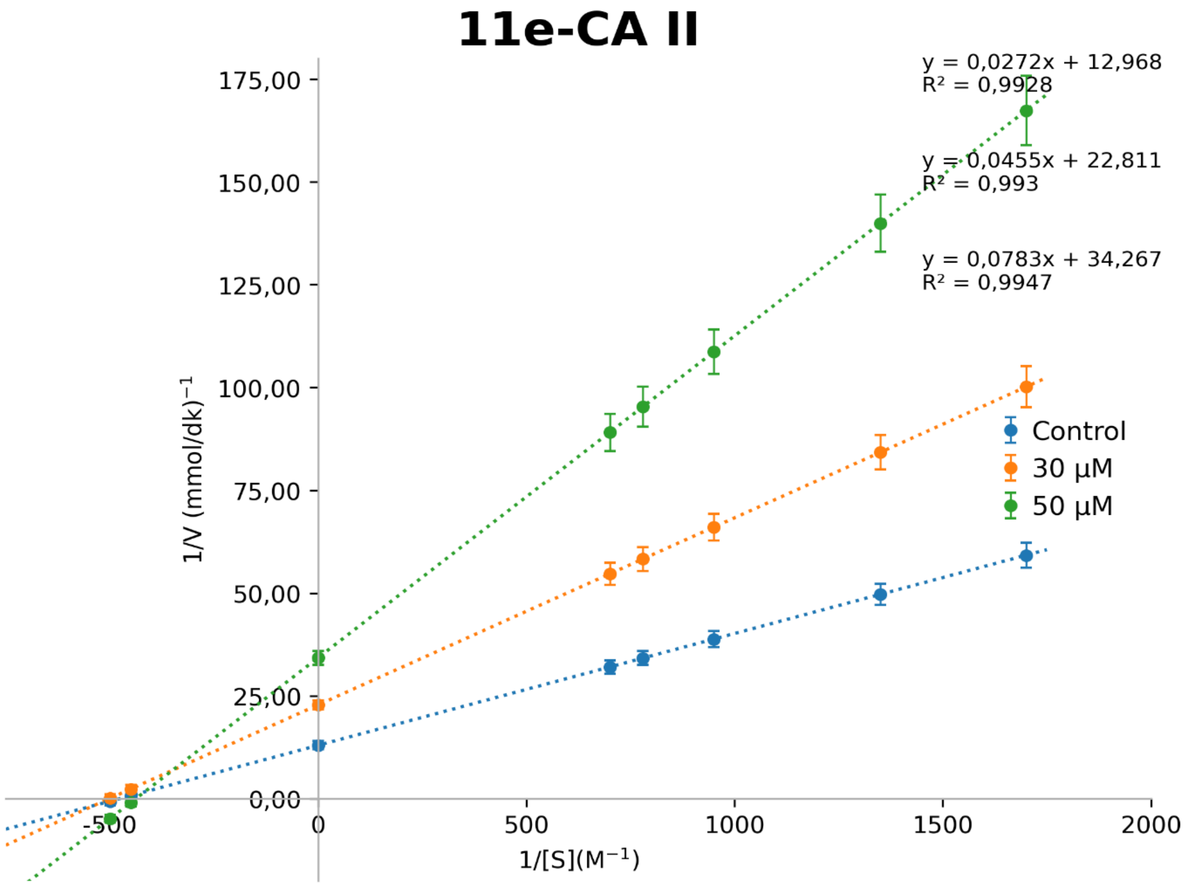
11e-CA II
y = 0,0272x + 12,968
R² = 0,9928
y = 0,0455x + 22,811
R² = 0,993
y = 0,0783x + 34,267
R² = 0,9947
Control
30 μM
50 μM
1/V (mmol/dk)⁻¹
1/[S](M⁻¹)

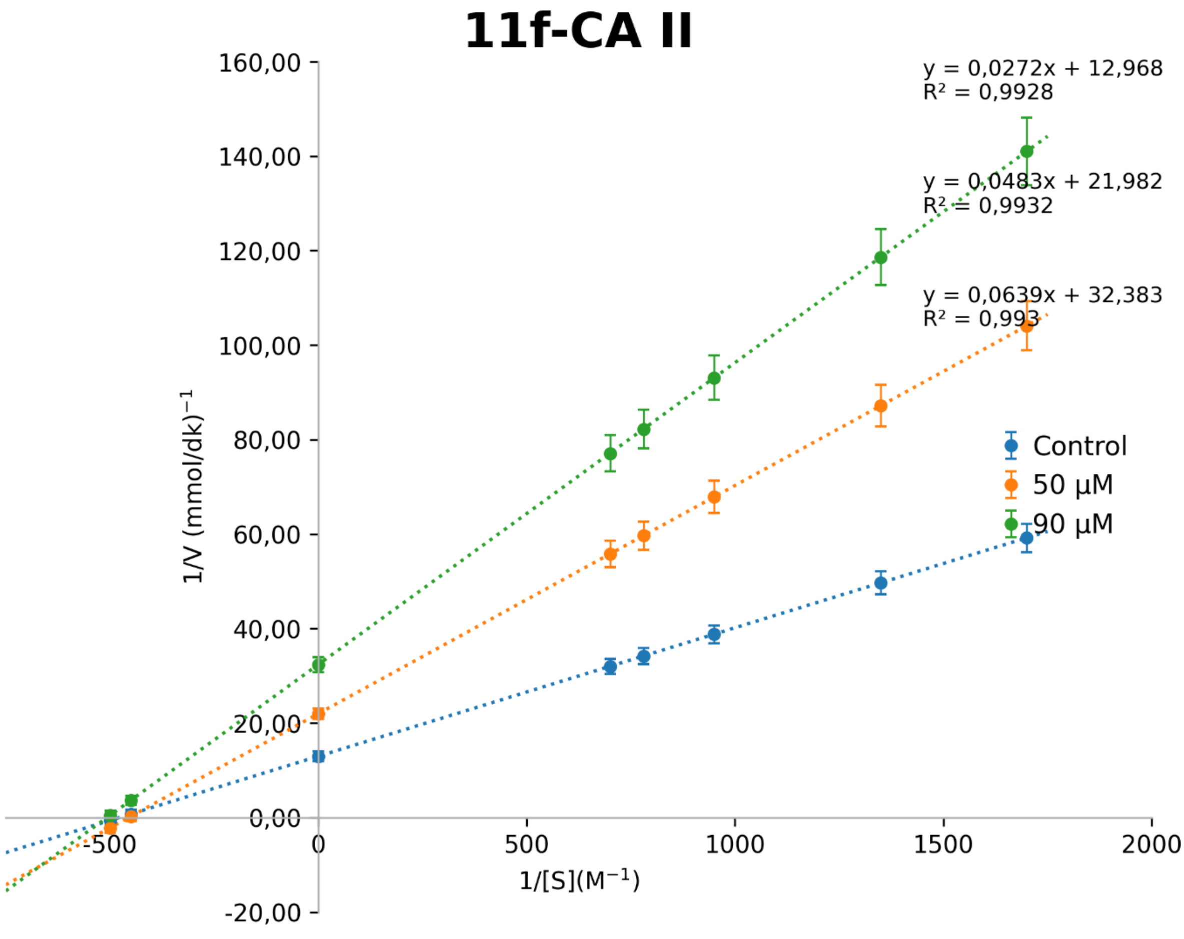
11f-CA II
y = 0,0272x + 12,968
R² = 0,9928
y = 0,0483x + 21,982
R² = 0,9932
y = 0,0639x + 32,383
R² = 0,993
Control
50 μM
90 μM
1/V (mmol/dk)⁻¹
1/[S](M⁻¹)

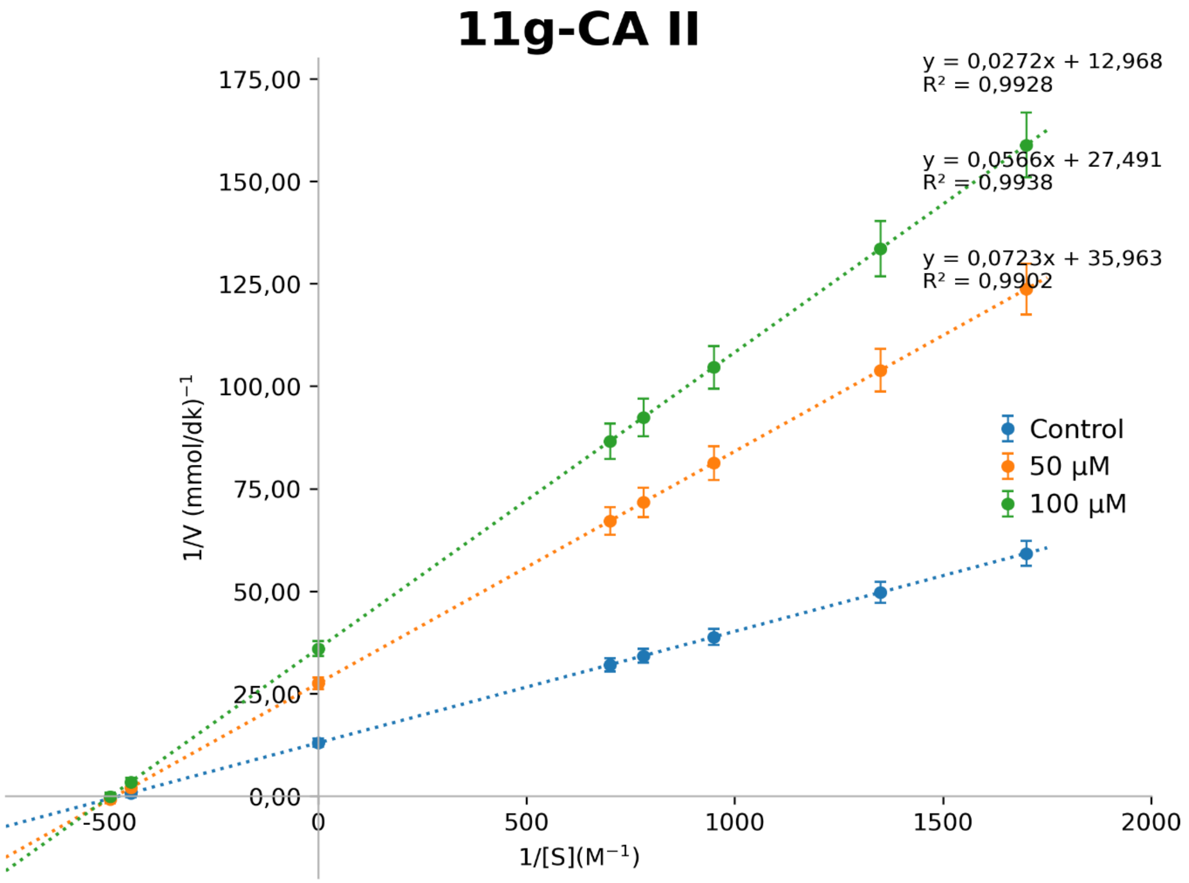
11g-CA II
y = 0,0272x + 12,968
R² = 0,9928
y = 0,0566x + 27,491
R² = 0,9938
y = 0,0723x + 35,963
R² = 0,9902
Control
50 µM
100 µM
1/V (mmol/dk)$^{-1}$
1/[S](M^{-1})
175,00
150,00
125,00
100,00
75,00
50,00
25,00
0,00
-500
0
500
1000
1500
2000

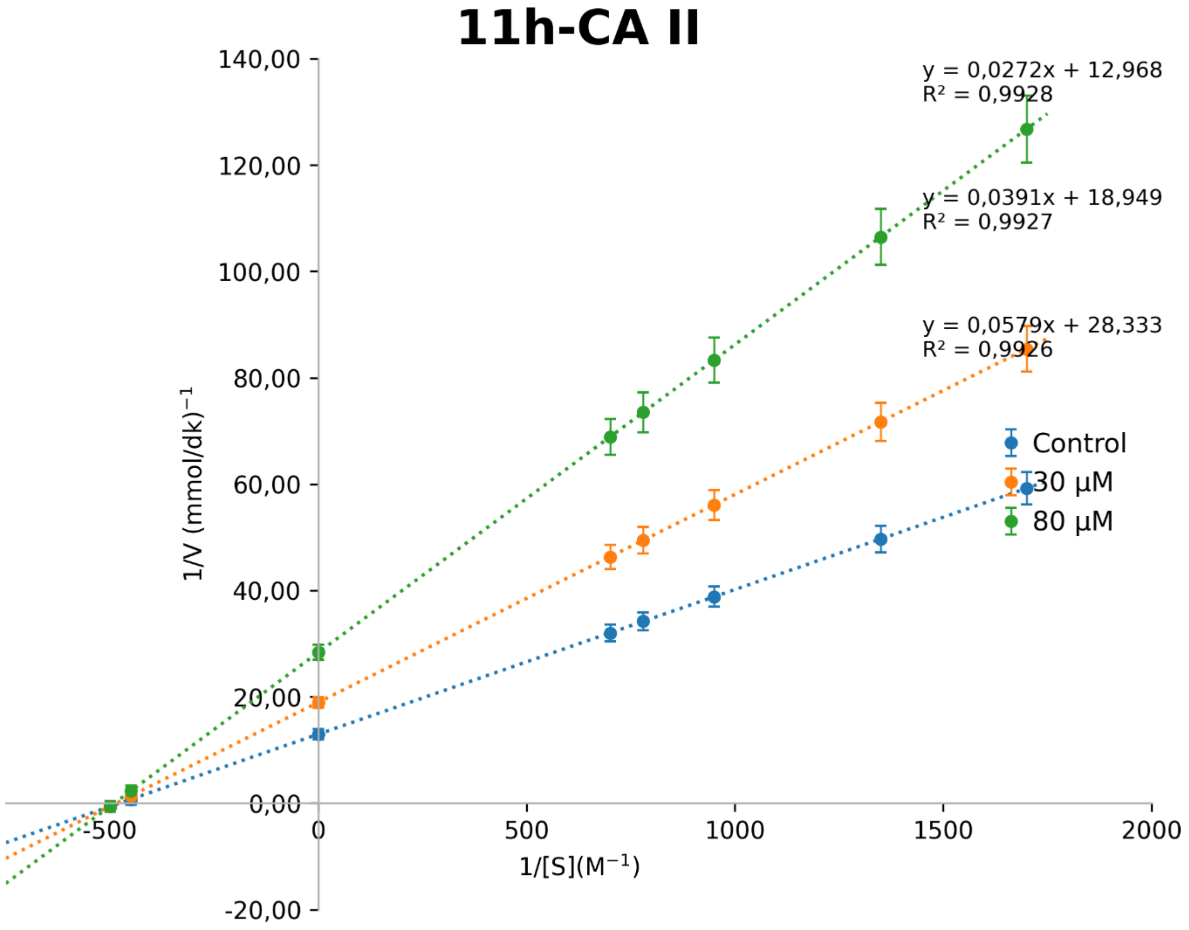
11h-CA II
y = 0,0272x + 12,968
R² = 0,9928
y = 0,0391x + 18,949
R² = 0,9927
y = 0,0579x + 28,333
R² = 0,9926
Control
30 µM
80 µM
1/V (mmol/dk)$^{-1}$
1/[S](M^{-1})
140,00
120,00
100,00
80,00
60,00
40,00
20,00
0,00
-20,00
-500
0
500
1000
1500
2000

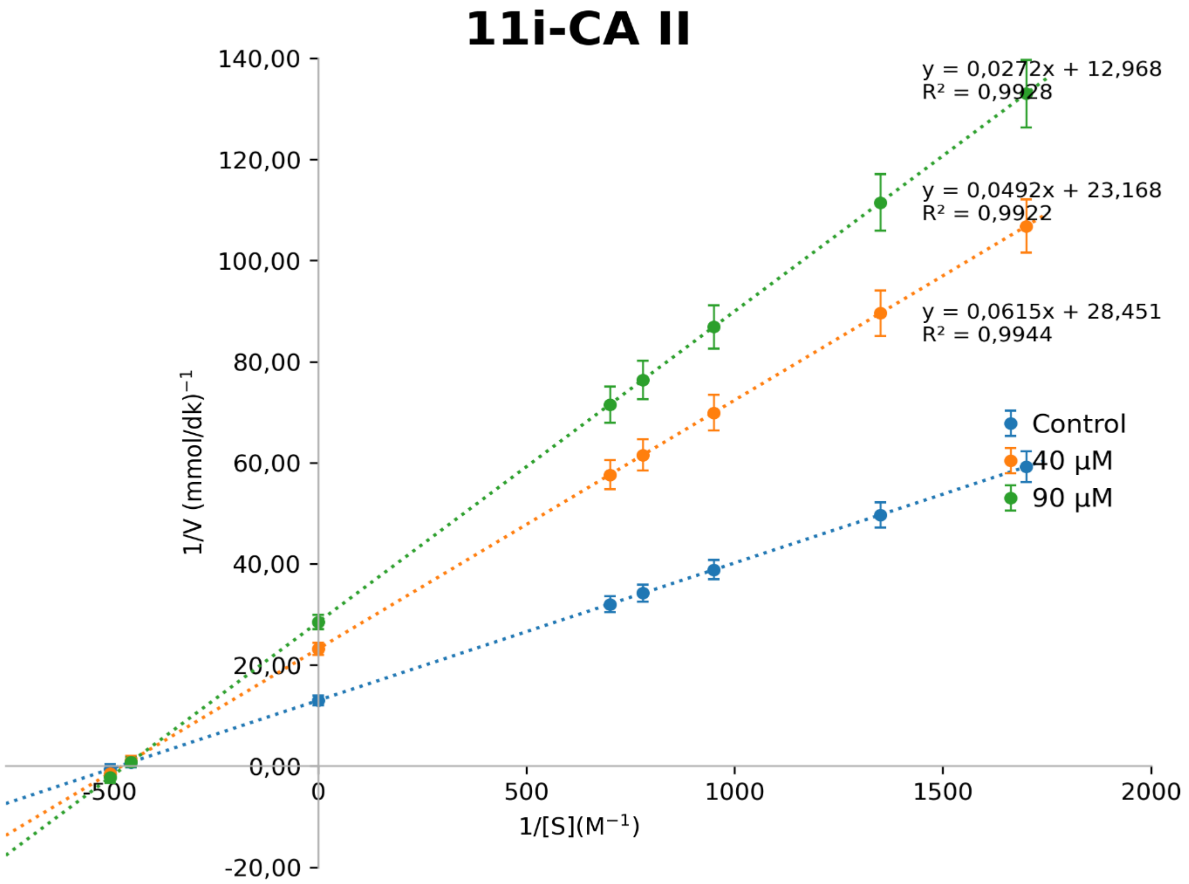
11i-CA II
y = 0,0272x + 12,968
R² = 0,9928
y = 0,0492x + 23,168
R² = 0,9922
y = 0,0615x + 28,451
R² = 0,9944
Control
40 μM
90 μM
1/V (mmol/dk)⁻¹
1/[S](M⁻¹)
140,00
120,00
100,00
80,00
60,00
40,00
20,00
0,00
-20,00
-500
0
500
1000
1500
2000

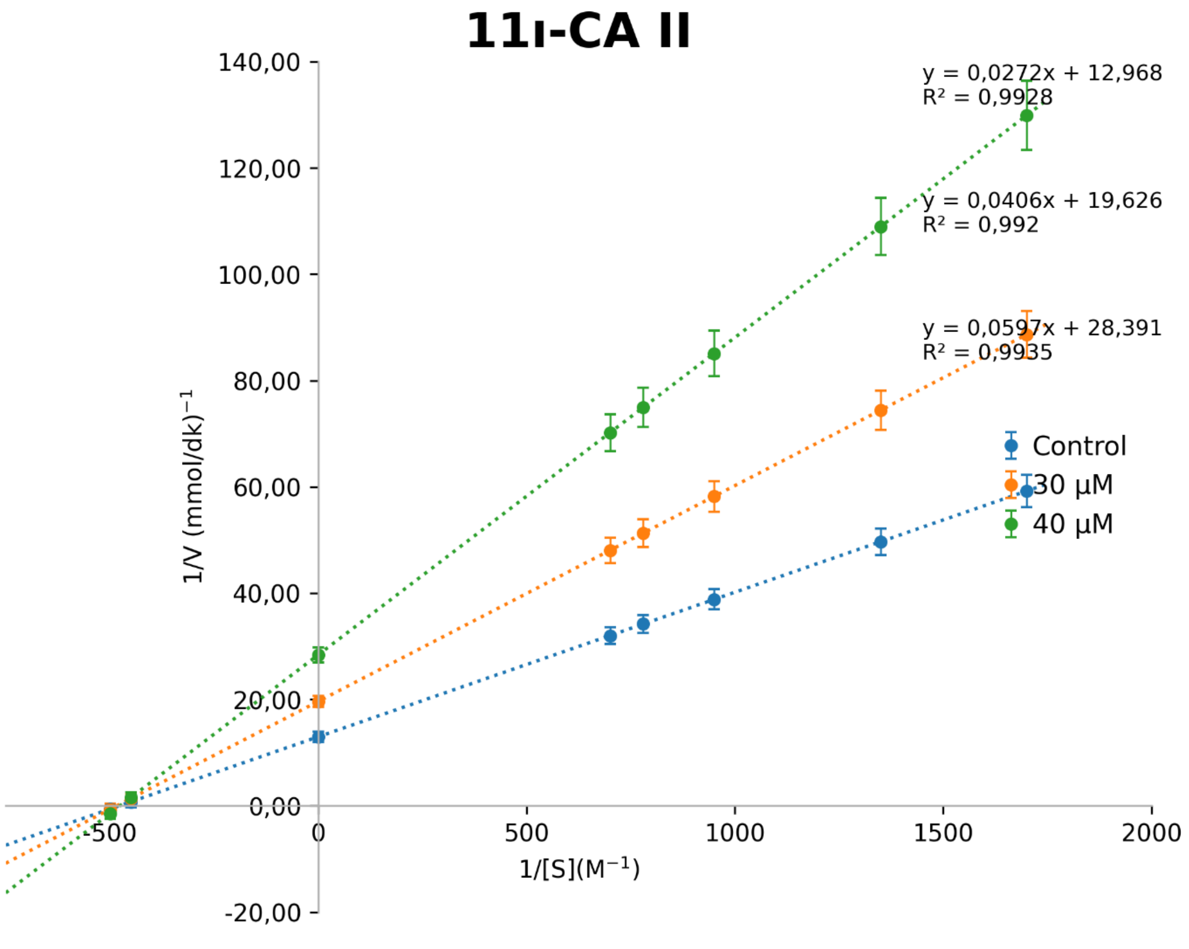
11ı-CA II
y = 0,0272x + 12,968
R² = 0,9928
y = 0,0406x + 19,626
R² = 0,992
y = 0,0597x + 28,391
R² = 0,9935
Control
30 μM
40 μM
1/V (mmol/dk)⁻¹
1/[S](M⁻¹)
140,00
120,00
100,00
80,00
60,00
40,00
20,00
0,00
-20,00
-500
0
500
1000
1500
2000

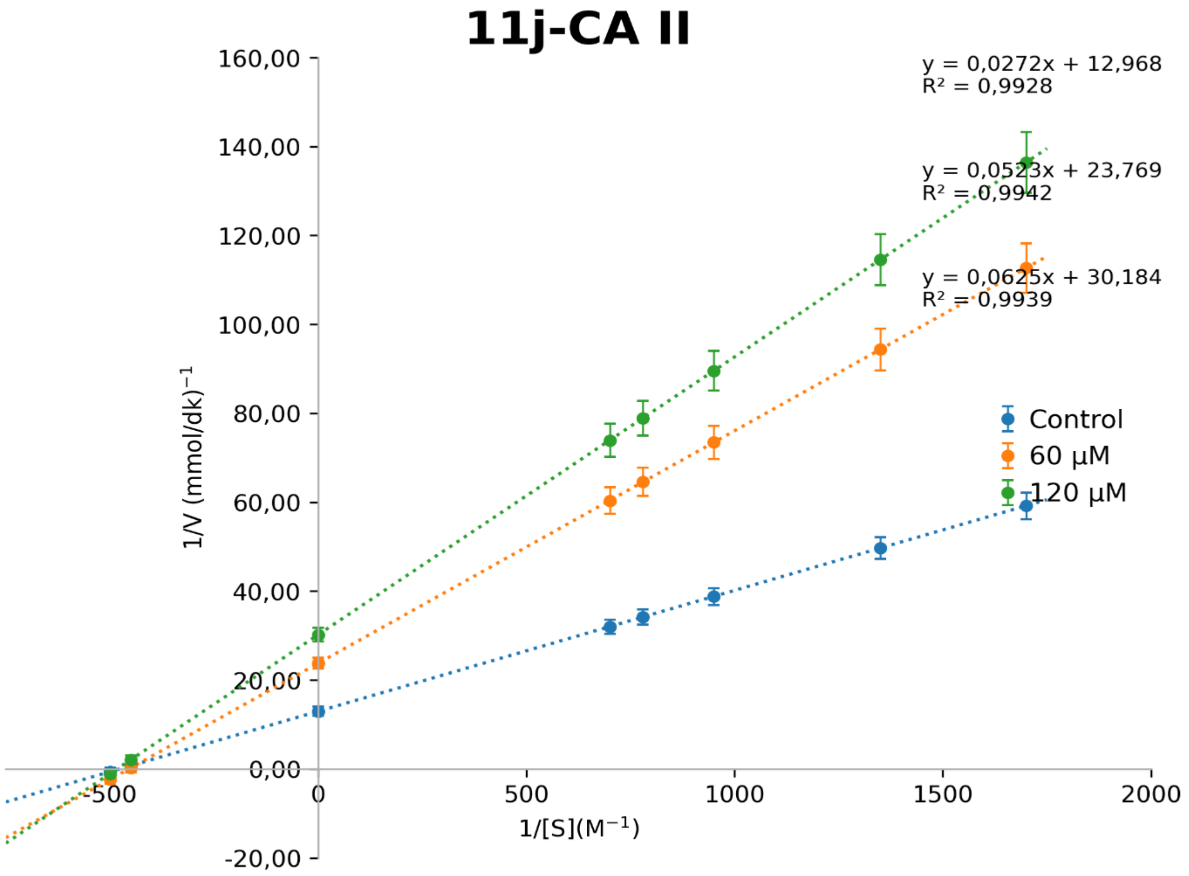
11j-CA II
y = 0,0272x + 12,968
R² = 0,9928
y = 0,0523x + 23,769
R² = 0,9942
y = 0,0625x + 30,184
R² = 0,9939
Control
60 µM
120 µM
1/V (mmol/dk)⁻¹
1/[S](M⁻¹)

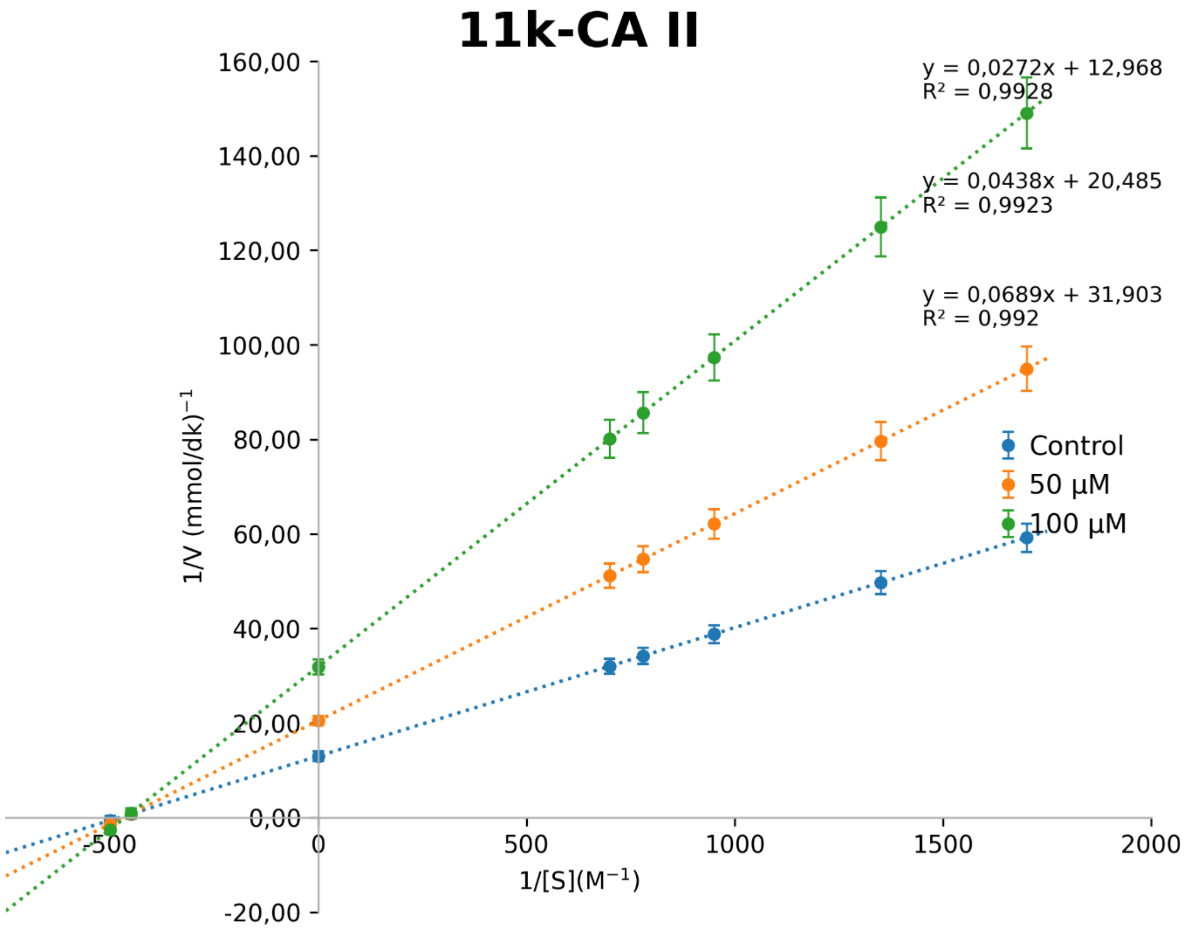
11k-CA II
y = 0,0272x + 12,968
R² = 0,9928
y = 0,0438x + 20,485
R² = 0,9923
y = 0,0689x + 31,903
R² = 0,992
Control
50 µM
100 µM
1/V (mmol/dk)⁻¹
1/[S](M⁻¹)

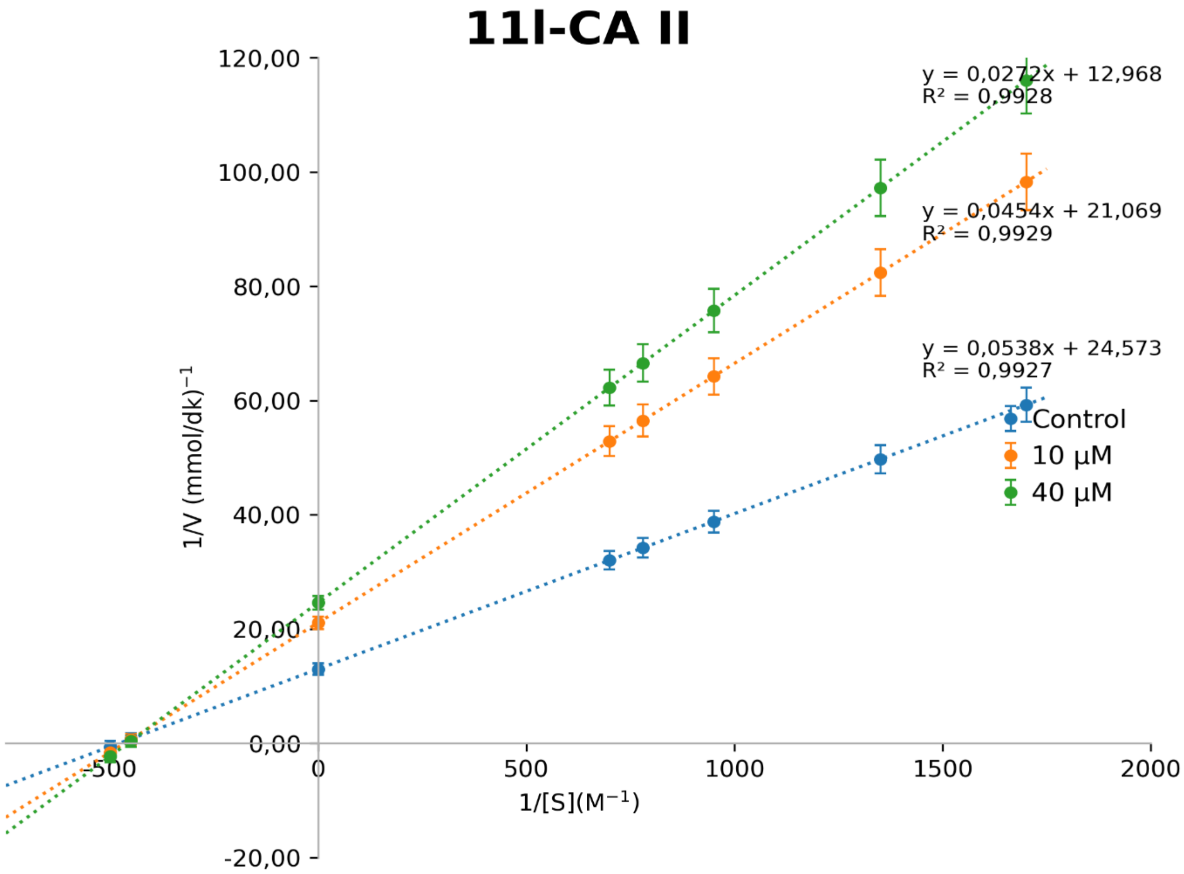
11l-CA II
y = 0,0272x + 12,968
R² = 0,9928
y = 0,0454x + 21,069
R² = 0,9929
y = 0,0538x + 24,573
R² = 0,9927
Control
10 µM
40 µM
1/V (mmol/dk)⁻¹
1/[S](M⁻¹)
120,00
100,00
80,00
60,00
40,00
20,00
0,00
-20,00
-500
0
500
1000
1500
2000

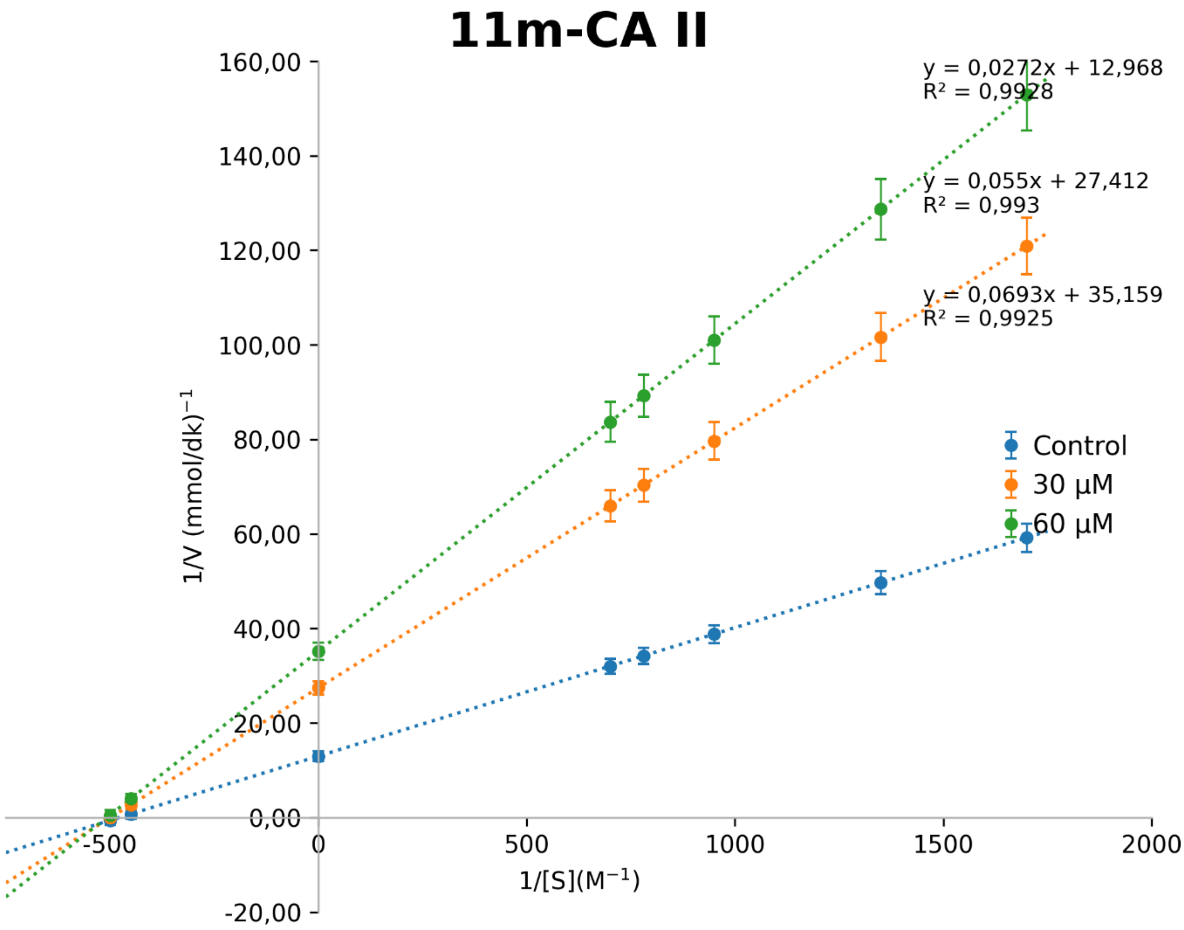
11m-CA II
y = 0,0272x + 12,968
R² = 0,9928
y = 0,055x + 27,412
R² = 0,993
y = 0,0693x + 35,159
R² = 0,9925
Control
30 µM
60 µM
1/V (mmol/dk)⁻¹
1/[S](M⁻¹)
160,00
140,00
120,00
100,00
80,00
60,00
40,00
20,00
0,00
-20,00
-500
0
500
1000
1500
2000

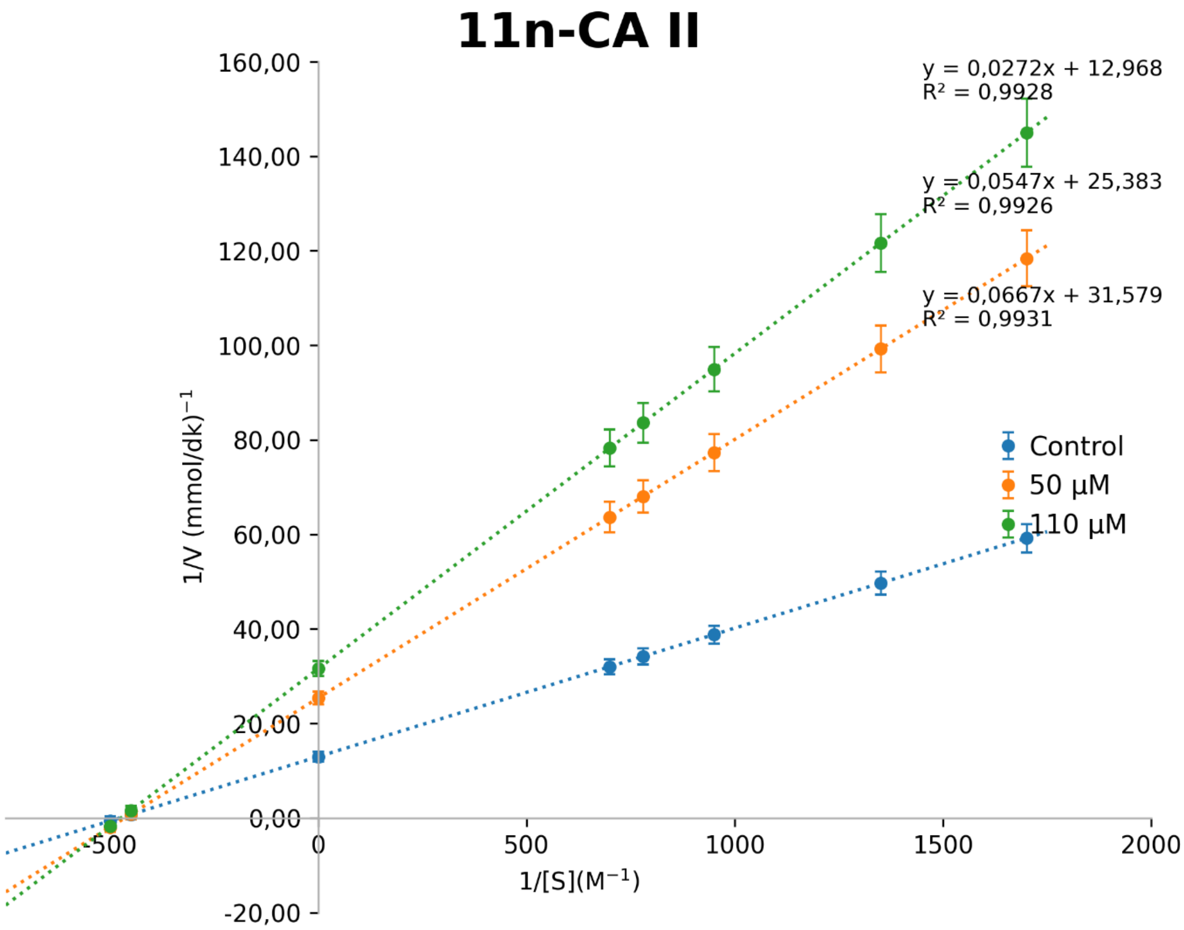

11n-CA II
y = 0,0272x + 12,968
R² = 0,9928
y = 0,0547x + 25,383
R² = 0,9926
y = 0,0667x + 31,579
R² = 0,9931
Control
50 µM
110 µM
1/V (mmol/dk)⁻¹
160,00
140,00
120,00
100,00
80,00
60,00
40,00
20,00
0,00
-20,00
-500
0
500
1000
1500
2000
1/[S](M⁻¹)
